# A first-in-human Phase I dose-escalation trial of the novel therapeutic peptide, ALM201, demonstrates a favourable safety profile in unselected patients with ovarian cancer and other advanced solid tumours

**DOI:** 10.1038/s41416-022-01780-z

**Published:** 2022-05-14

**Authors:** Aya El Helali, Ruth Plummer, Gordon C. Jayson, Vicky M. Coyle, Yvette Drew, Nerissa Mescallado, Noor Harris, Andrew R. Clamp, Janine McCann, Helen Swaisland, Richard D. Kennedy, Aaron N. Cranston, Richard H. Wilson

**Affiliations:** 1 Patrick G. Johnston Centre for Cancer Research, Queen’s University Belfast, Belfast, UK; 2 Newcastle University Translational and Clinical Research Institute, Newcastle University, Newcastle upon Tyne, UK; 3 Institute of Cancer Sciences and Christie Hospital, University of Manchester, Manchester, UK; 4 Northern Ireland Cancer Research Consumers’ Forum, Belfast, UK; 5 Therakin Consulting Ltd., Sandbach, UK; 6 Almac Diagnostic Services, Craigavon, UK; 7 Almac Discovery Ltd., Belfast, UK; 8 Present Address: University of Hong Kong, 21 Sassoon Road, Pok Fu Lam, Hong Kong; 9 Present Address: Wolfson Wohl Cancer Research Centre, Institute of Cancer Sciences, University of Glasgow, Garscube Estate, Switchback Road, Glasgow, G61 1BD UK

**Keywords:** Drug development, Drug safety

## Abstract

**Background:**

We aimed to assess the safety, tolerability and pharmacokinetics of a novel anti-angiogenic peptide.

**Methods:**

We used an open-label, multicentre, dose-escalation Phase I trial design in patients with solid tumours. ALM201 was administered subcutaneously once daily for 5 days every week in unselected patients with solid tumours.

**Results:**

Twenty (8 male, 12 female) patients with various solid tumours were treated (18 evaluable for toxicity) over eight planned dose levels (10–300 mg). ALM201 was well-tolerated at all dose levels without CTCAE grade 4 toxicities. Adverse events were predominantly grades 1–2, most commonly, localised injection-site reactions (44.4%), vomiting (11%), fatigue (16.7%), arthralgia (5.6%) and headache (11%). Thrombosis occurred in two patients at the 100 mg and 10 mg dose levels. The MTD was not reached, and a recommended Phase II dose (RP2D) based on feasibility was declared. Plasma exposure increased with dose (less than dose-proportional at the two highest dose levels). No peptide accumulation was evident. The median treatment duration was 11.1 (range 3–18) weeks. Four of 18 evaluable patients (22%) had stable disease.

**Conclusions:**

Doses up to 300 mg of ALM201 subcutaneously are feasible and well-tolerated. Further investigation of this agent in selected tumour types/settings would benefit from patient-selection biomarkers.

## Introduction

Angiogenesis is a critical mechanism in tumour development and growth of tumours [1, 2] and is considered a Hallmark of Cancer [1]. Understanding the mechanisms underpinning angiogenesis has continued to improve and has led to many biological and small-molecule anti-angiogenic agents being approved as cancer therapies for various solid tumours [2–5]. To date, the majority of approved anti-angiogenic therapies have targeted signalling through receptor tyrosine kinases and growth factor expression, particularly VEGF, and used inhibition of these pathways as their primary mechanism of action. However, limited efficacy and unwanted side effects can be significant problems, and patients receiving tyrosine kinase inhibitors have a high rate of resistance after several cycles of therapy [6–8]. Nevertheless, there is growing uptake of anti-angiogenic therapy as a standard therapeutic approach, particularly for treating ovarian cancer [9–12].

ALM201 is a novel 23-amino acid synthetic peptide under development by Almac Discovery to treat solid cancers and identified from the endogenous human protein, FKBPL (FK506 binding protein-like). Initially, the full-length protein was investigated and found to have anti-angiogenic activity using in vitro and ex vivo angiogenesis assays [13]. The sequence responsible for this anti-angiogenic activity was identified using a series of truncated peptides from the protein and found to be a 24-amino acid sequence near the N-terminus. A 24-mer peptide was generated by chemical synthesis matching the identified sequence and assessed in several in vitro, ex vivo, and in vivo experiments. This peptide (termed AD-01) had anti-angiogenic efficacy equivalent to that of the entire protein [14]. However, AD-01 became unstable due to the formation of the inactive pyro-glutamate. A second-generation peptide, ALM201, was manufactured by removing the N-terminal glutamine generating a peptide of similar potency and efficacy to AD-01 in a range of pre-clinical studies, including in vivo tumour xenograft models. The ALM201 peptide was nominated as the clinical candidate.

ALM201 showed promising low picomolar activity in a range of standard in vitro and ex vivo assays that measure angiogenesis and cell migration and invasion [15]. Early investigations into the mechanism of action for ALM201 reported that the peptide is internalised into CD44-expressing cells and targets microtubules inhibiting migration and invasion in cancer and endothelial cells, similar to vascular disrupting agents [16, 17]. However, it was unclear if this was the principal mechanism of inhibiting angiogenesis or if other factors, such as inhibiting endothelial cell migration and invasion following stimulation with multiple growth factors, played a significant role.

ALM201 was not immunogenic and did not cause cytotoxicity or effects on the cell cycle or cell proliferation, suggesting that it would have limited or no off-target effects and that its mode of action was truly anti-angiogenic. Furthermore, ALM201 had significant anti-tumour activity in vivo, as a single agent or in combination with chemotherapeutic agents, in tumour xenograft models (Almac Discovery, manuscript in preparation), making it an attractive agent to take into the clinic.

ALM201 was subsequently granted orphan drug designation for ovarian cancer by the FDA in 2017 [18]. When considered together, the pre-clinical efficacy, systemic PK exposure, tolerability and safety profile of ALM201 in pre-clinical models indicated sufficient clinical need and merit to advance this novel anti-angiogenic peptide into first-in-patient clinical trials.

Here, we report the first-in-human trial with ALM201. The primary objectives of this study were to characterise the safety and tolerability of ALM201 in patients with solid tumours.

## Methods

### Patient selection

Patients with advanced solid tumours, for whom treatment with an anti-angiogenic agent was considered appropriate, were enrolled. Specifically, the trial included patients with histologically and/or cytologically confirmed advanced solid tumours for whom no standard effective therapy was available or likely to be of limited efficacy and in whom a rationale for using an anti-angiogenic treatment approach exists. In addition, previous use of anti-angiogenic therapy was allowed if previously well-tolerated. It was anticipated that up to 30 patients with advanced cancer would be enrolled in this study.

### Patient eligibility

Patients with histologically confirmed locally advanced or metastatic solid tumours, refractory to conventional therapy or for which no standard treatment exists, were eligible provided they met the following criteria: evaluable disease as assessed by RECIST 1.1 [19]; age ⩾18 years; Eastern Co-operative Oncology Group (ECOG) performance status (PS) 0–1; adequate haematopoietic (absolute neutrophil count [ANC] ⩾1.5 × 10^9^/L, platelet count ⩾100 × 10^9^/L, haemoglobin ⩾9 g/dL (not transfusion dependent)); hepatic (AST ≤2.5 times upper limit of normal (ULN), ALT ≤2.5 times the ULN; ≤5 times the ULN for patients with advanced solid tumours with liver metastases, bilirubin <1.5 ULN); patients with confirmed bone metastases were permitted on the study with isolated elevations in ALP <5 times the ULN; renal function (serum creatinine ≤1.5 times the ULN or estimated glomerular filtration rate (GFR) of >50 mL/min calculated by the Cockcroft–Gault Formula [20], urine protein ≤2 + as measured by dipstick); coagulation (prothrombin time or APTT ≤1.3 times the ULN); a negative pregnancy test for females of child-bearing potential; and the ability to give written, informed consent prior to any study-specific screening procedures, with the understanding that consent could be withdrawn by the patient at any time without prejudice.

Exclusion criteria included the following: inability to tolerate anti-angiogenic therapies e.g. proteinuria; prior thromboembolic events; previous history of bowel obstruction, clinical evidence of gastrointestinal obstruction, large burden of peritoneal disease or evidence of bowel involvement on computed tomography; symptomatic or uncontrolled intracranial metastases or primary intracerebral tumours or leptomeningeal involvement; history of clinically significant cardiac condition, including uncontrolled hypertension (BP > 140/90 mmHg, despite medical therapy), left ventricular systolic dysfunction (ejection fraction <55% on echocardiography) with or without heart failure symptoms, history of an ischaemic cardiac event within 3 months of study entry (myocardial infarction, acute coronary syndrome), QT interval prolongation (QTcF, Fridericia’s Correction of >450 ms on screening 12-lead ECG), clinically significant cardiac arrhythmia within 3 months of study entry; known history of human immunodeficiency virus; active hepatitis B or C; and patients on therapeutic anti-coagulants.

### Study design

 Eligible participants were enrolled in sequential cohorts treated with a subcutaneous (SC) injection of ALM201, with close monitoring for safety and dose-limiting toxicities (DLTs). Dose levels were not weight-adjusted, and the starting dose for the study was 10 mg ALM201 given on days 1–5, 8–12 and 15–19 every 21 days, i.e., weekday dosing to be given continuously. Inter-cohort dose-escalation increments did not exceed 100% and were guided by safety data observed during cycle one and ongoing safety assessment beyond cycle one in earlier cohorts. Every new dose cohort was evaluated for the occurrence of a DLT during treatment cycle 1.

This study commenced with an accelerated dose-escalation design [21], then switched to a conventional algorithm (3 + 3 patients per dose level) to identify the maximum tolerated dose (MTD), escalating on observation of 0/3 or 1/6 DLTs, and de-escalating if two or more patients with DLTs were encountered. Dose escalation of three patient cohorts proceeded according to the scheme presented in Table 1.

**Table 1:** Study design showing dose escalation and cohort identity.

Cohort	Escalation step	Dose level (mg)	Number of patients
1	Starting dose	10	1
2	2× starting dose	20	1
3	4× starting dose	40	1
4	8× starting dose	80	3
5	16× starting dose	160	3
6	20× starting dose	200	4
7 (MFD)	30× starting dose	300	3
8	CRC recommended dose	100	4

*MFD* maximum feasible dose, *CRC* cohort review committee.

Dose levels (in milligrams) for each dose cohort, fold-escalation from starting dose, and the number of patients in each cohort are shown.

### Drug formulation and administration

For clinical dosing, the ALM201 drug substance was formulated as an aqueous solution containing 80 mM sodium carbonate, 20 mM Tris, 25 mM sodium chloride, pH 6.5 for subcutaneous administration and supplied as a 100 mg/mL sterilised drug product solution for subcutaneous (SC) injection.

ALM201 was administered as an SC injection with a maximum administration volume of 1.0 mL per injection. Each 1 mL of injection contained up to 100 mg of ALM201. A maximum of 3 × 1.0 mL injections were given for any single dose. Therefore, the maximal feasible dose (MFD) was 300 mg. The starting dose level of ALM201 was 10 mg. Patients with advanced ovarian cancer or other solid tumours received ALM201 by subcutaneous administration at dose levels of 10, 20, 40, 80, 100, 160, 200 or 300 mg given once daily on days 1–5, 8–12 and 15–19 every 21 days. Between 1 and 4 patients were treated at each dose level.

The SC injection was administered either in the abdomen, leg or arm and in adherence with local administration guidelines, including premedication if required for local injection-site reactions.

The study permitted a maximum of eight three-weekly treatment cycles. Patients whose disease had not progressed and had not been withdrawn due to toxicity were eligible to receive additional cycles of ALM201. Intra-patient dose escalation was not permitted.

### Study assessment

Patients had scheduled clinic visits on every dosing day of the first cycle, then on days 1, 8 and 15 of cycles 2 to 4. Patients were only required to visit the clinic on day 1 of each subsequent cycle from cycle five. On all other days, ALM201 was administered at home by nurses.

### Safety evaluation

Safety assessments included physical examination, vital signs, biochemistry and haematology laboratory screens, plus immunogenicity testing. Safety evaluations were conducted weekly during each treatment cycle, with DLT assessed during cycle one only. All events and suspected DLTs were graded according to the Common Toxicity Criteria for Adverse Events (CTCAE), version 4.03.

### Pharmacokinetic sampling

Peripheral blood samples were taken before and after dose administration on day 1, day 3, and day 18 of cycle 1 of treatment, pre-dose on day 1 of cycles 2, 3, 4, 5, and 6 of treatment, and pre-and post dose on day 18 of cycles 2, 4 and 6 of treatment. On day 1 of cycle 1, blood samples were taken pre-dose (0 h) and at 15 and 45 min, 1.5, 2, 3, 4, 5, 6 and 22 h post dose, with additional samples taken at 7 and 8 h post dose from patients dosed at the 300 mg dose level. On subsequent sampling occasions, blood samples were taken pre-dose (0 h) and at 30 min, 1, 1.5, 2, 3.5, and 5 h post dose, except for patients who received the 300 mg dose whose samples were taken pre-dose (0 h) and at 30 min, 1, 1.5, 2, 4.5 and 7 h post dose (Table 2). In addition, on day 1 of cycle 1, urine was collected from patients over 6 h after dosing to determine potential renal excretion of the parent ALM201 peptide.

**Table 2:** Patient demographics.

Characteristics		Number of patients
Total		20
Women		12
Men		8
Age (years)		
Median		61
Range		31–74
ECOG PS		
0		8
1		12
Tumour sites		
**Primary tumour type**	**Histologic diagnosis**	**Number of patients**
Cervical	Squamous cell carcinoma	1
Colorectal	Adenocarcinoma	4
Endometrial	Adenocarcinoma	2
Gall bladder	Adenocarcinoma	2
Mesothelioma	Epithelioid	1
NSCLC	Adenocarcinoma	1
	Mucinous adenocarcinoma	1
Ovarian	Serous carcinoma	5
Pancreatic	Adenocarcinoma	1
Renal	Clear cell carcinoma	1
Urachus	Adenocarcinoma	1
Prior lines of systemic anticancer therapy		
Median number of lines of prior systemic anticancer therapy		3
Range		1–9
Prior anti-angiogenic agents		
Yes		4
No		16
Number of Line(s) of prior anti-angiogenic agents	1-Line(s)	2
	2-Line(s)	1
	3-Line(s)	1

*ECOG PS* Eastern Cooperative Oncology Group Performance Status.

Patient sex, age at recruitment, ECOG performance status, primary tumour site and histological type are shown, along with information on prior anticancer treatments.

Plasma and urine concentrations of ALM201 were measured by validated liquid chromatographic tandem mass spectrometric (LC-MS/MS) methods. Pharmacokinetic parameters from the plasma concentration data were calculated using the computer programme Phoenix WinNonlin version 6.3 (Pharsight Corporation, USA); pharmacokinetic parameters from the urine concentration data were calculated using Microsoft Excel 2016. Summary statistics and plots of the data were generated in Microsoft Excel 2016. The amount (mg) of ALM201 excreted unchanged in the urine was calculated from the product of the urine concentration and the volume of urine produced during the collection period. This was converted into a % dose excreted by dividing by the administered daily dose.

The extent of accumulation with multiple dosing was assessed by comparing the C_max_ and AUC_0-t_ values of day 1 and day 18 of cycle 1.

A preliminary assessment of dose proportionality across the dose range used in the study was performed on day 1 cycle 1 C_max_ and AUC_0-t_ using a linear regression power model:

$$\begin{array}{l}{{{{{{{\mathrm{parameter}}}}}}}} = {{{{{{{\mathrm{exp}}}}}}}}\left( {{{{{{{\mathrm{a}}}}}}}} \right) \ast \left( {{{{{{{{\mathrm{dose}}}}}}}}} \right){{{{{{{\mathrm{b}}}}}}}}\\ {{{{{{{\mathrm{i}}}}}}}}{{{{{{{\mathrm{.e}}}}}}}}{{{{{{{\mathrm{.}}}}}}}}\,{{{{{{{\mathrm{log}}}}}}}}_{{{{{{{\mathrm{e}}}}}}}}\left( {{{{{{{{\mathrm{parameter}}}}}}}}} \right) = {{{{{{{\mathrm{a}}}}}}}} + \left( {{{{{{{{\mathrm{b}}}}}}}} \ast {{{{{{{\mathrm{log}}}}}}}}_{{{{{{{\mathrm{e}}}}}}}}\left( {{{{{{{{\mathrm{dose}}}}}}}}} \right)} \right)\end{array}$$

where a is the intercept and b is the slope measuring the extent of dose proportionality. Following the fitting of the model to the data, if the slope parameter value was <1.0, it was concluded that the PK parameter increase was less than dose-proportional.

### Radiological assessment

Tumour assessment by imaging (CT scan or MRI scan as appropriate for tumour type) was assessed in all patients at initial screening and after every two cycles of treatment (i.e., every 6 weeks) during cycles 1–8 (i.e., first 24 weeks), and then after every four cycles of treatment (i.e., every 12 weeks) from cycle nine onwards. Scans were reported according to RECIST version 1.1.

### Statistical analysis

Descriptive statistics were used to summarise the safety data and the systemic pharmacokinetics of ALM201. In addition, summary statistics for the concentration data and PK parameters and plots of geometric mean concentration data and PK parameters were generated to assess the extent of accumulation on multiple dosing, a preliminary assessment of dose proportionality, and determination of the percentage dose excreted in the urine as parent molecule.

## Results

### Patient demographics and clinical characteristics

Between July 2015 and January 2017, 20 patients (8 male, 12 female) were enrolled across three participating Experimental Cancer Medicine Centres (ECMC) in the UK. Eighteen patients were evaluated for safety. Two patients (one in the 200 mg dose level (cohort 6) and one in the 100 mg dose level (cohort 8)) were not evaluable due to rapidly progressive disease. The median performance status (PS) was 1 (60%, range: 0–1), and the median age was 61 years (range: 31–74 years). Diagnostic and demographic data for the patient cohort are summarised in Table 2.

Patients were treated in separate cohorts over seven dose levels, ranging from 10 to 300 mg. On reaching the MFD of 300 mg, we noted that there were no DLTs in dose level 6 and dose level 7 which were at 200 mg and 300 mg and had a total of 7 patients. Thus, an additional eighth cohort (at the lower and previously unexplored dose of 100 mg) was agreed between the investigators and added. Based on data from pre-clinical studies, 100 mg was expected to result in sufficient plasma exposure for efficacy. In addition, 100 mg could be administered in a single 1.0 mL injection, avoiding the need for multiple injections. The median duration of treatment was 11.1 weeks (range: 3–18 weeks) (Supplementary Fig. 1).

### Safety profile

**Fig. 1:**
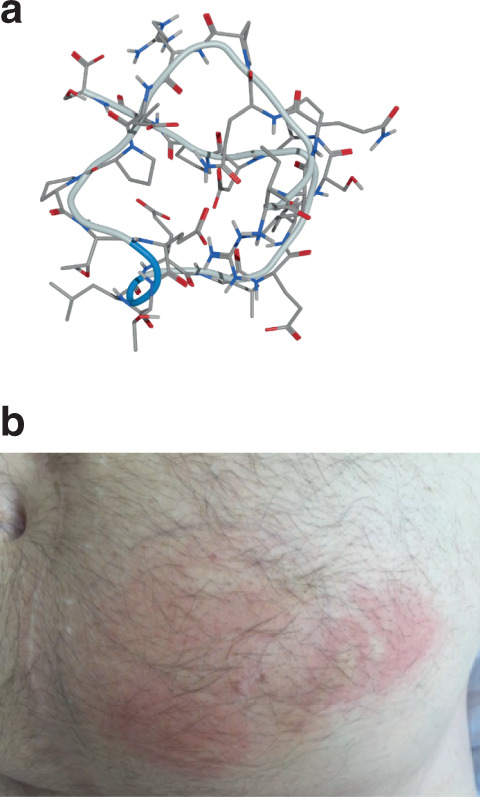
Molecular structure and safety of ALM201. **a** Ball-and-stick illustration of the predicted molecular structure of ALM201 (2575 Da) in a low-energy state (Image courtesy of Dr. Oliver Barker, Almac Discovery). **b** Image taken of a localised abdominal wall skin reaction post-ALM201 SC injection. The umbilicus gives an idea of the size of the reaction (patient consent was given for imaging and use for publication).

In general, ALM201 (Fig. 1a) was very well-tolerated at all dose levels studied. There were no CTCAE grade 4-related toxicities. Adverse events throughout the entire study were predominantly of grade 1 or 2 in severity and similar (Table 3). The most common adverse events were localised injection-site reactions (44.4%, see Fig. 1b), vomiting (11%), fatigue (16.7%), arthralgia (5.6%) and headache (11%). In addition, thrombotic events occurred in two patients, cohort 8 (100 mg dose level) and cohort 1 (10 mg dose level) (Table 3). The two thrombotic events recorded in this study were a renal vein thrombosis in a patient with cervical cancer and a pulmonary embolus in a patient with mesothelioma. Both events were not regarded as DLTs as they occurred at an intermediate dose level investigated in our final cohort with earlier dosing up to three times higher than this. No other drug-associated safety issues were reported. In addition, no clinically significant changes were observed in biochemical, coagulation, or haematologic parameters. No DLTs were observed. The MTD was not reached even with subcutaneous weekday doses of up to 300 mg. Furthermore, there were no DLTs reported in the four patients recruited to cohort 8 and in the seven patients in cohorts 6 and 7. Therefore, given this safety profile, the 100 mg dose was deemed tolerable, feasible and safe RP2D. Therefore, ALM201 has been deemed a safe anti-angiogenic agent with toxicities predominantly ≤grade 2 CTCAE.

**Table 3:** Summary of adverse events and causality.

Adverse events	Number of patients					
	Total	Grade 1	Grade 2	Grade 3	Grade 4	
Injection-site mediated toxicities						**Causality**
Injection-site immune reaction	1 (5.6%)	0	1 (5.6%)	0	0	Definite
Injection-site erythema	7 (38.9%)	7 (38.9%)	0	0	0	Definite
Abdominal wall bruising	3 (16.7%)	3 (16.7%)	0	0	0	Definite
Injection-site pain	1 (5.6%)	1 (5.6%)	0	0	0	Definite
Pruritus	1 (5.6%)	1 (5.6%)	0	0	0	Definite
Fatigue	3 (16.7%)	1 (5.6%)	2 (11%)	0	0	Possible
Pedal oedema	1 (5.6%)	1 (5.6%)	0	0	0	Possible
Arthralgia	1 (5.6%)	1 (5.6%)	0	0	0	Possible
Hot flushes	1 (5.6%)	1 (5.6%)	0	0	0	Possible
Thrombosis						
Pulmonary embolus	1 (5.6%)	0	0	1 (5.6%)	0	Possible
Venous thrombus	1 (5.6%)	0	0	1 (5.6%)	0	Possible
Gastrointestinal toxicities						
Diarrhoea	2 (11%)	2 (11%)	0	0	0	Possible
Nausea	1 (5.6%)	0	1 (5.6%)	0	0	Possible
Vomiting	2 (11%)	2 (11%)	0	0	0	Possible
Anorexia	1 (5.6%)	1 (5.6%)	0	0	0	Possible
Infections						
Pyrexia	1 (5.6%)	1 (5.6%)	0	0	0	Possible
Oral candidiasis	1 (5.6%)	1 (5.6%)	0	0	0	Possible
Urinary tract infection	1 (5.6%)	0	1 (5.6%)	0	0	Possible
Neurological toxicities						
Blurred vision	1 (5.6%)	1 (5.6%)	0	0	0	Probable
Headache	2 (11%)	2 (11%)	0	0	0	Possible
Depression	1 (5.6%)	0	1 (5.6%)	0	0	Possible

The number (percentage), type, and grade of adverse events are shown, along with an assessment of the likelihood of causality.

### Pharmacokinetic profile of ALM201 following a single SC dose

Following administration of the first subcutaneous dose of ALM201 (day 1 of cycle 1), absorption was relatively rapid, with maximum plasma concentrations observed between 0.75 and 4.0 h after dosing (summarised in Table 4). The median *T*_max_ across the dose groups ranged from 1.5 to 2.5 h. Higher *T*_max_ values tended to be more prevalent in the 160, 200 and 300 mg cohorts, possibly reflecting the multiple, sequential injections needed to administer these higher doses. Following the peak, plasma concentrations declined in a mono-exponential manner falling below the limit of quantification of the assay (100 ng/mL) by 4–5 h after dosing in the 10, 20 and 40 mg cohorts and by 22 h in the 80, 100, 160, 200 and 300 mg cohorts. In most cases, estimations of half-life could not be reliably determined because plasma levels were often below the lower limit of quantification (LLOQ) of the assay. In the three profiles where half-life could be determined (patients 02.002 (80 mg cohort), 02.003, and 03.005 (both 160 mg cohort)), it was short, at ~1.5 h (1.80, 1.50 and 0.90 h, respectively). Other PK parameters such as AUC_0-∞_, apparent plasma clearance and apparent volume of distribution could not be derived from the data because reliable values for the terminal elimination rate constant λ_z,_ could not be determined. Overall, exposure parameters typically showed low to moderate inter-patient variability (1.2- to threefold range of values; CVs of 10 to 50%). However, the observed range of values in the 100 mg cohort was much higher (approximately ninefold). This was due predominantly to the very low exposure seen in patient 03.012; excluding that patient reduced the range to approximately twofold. The derived pharmacokinetic parameters are presented in Table 4. The supporting geometric mean plasma concentrations are summarised in Supplementary Table S1.

**Table 4:** Pharmacokinetic parameters and summary statistics for ALM201 following subcutaneous administration on days 1, 3, and 18 of cycle 1 at each of the discrete dose levels of 10, 20, 40, 80, 100, 160, 200 and 300 mg.

Parameter	Summary	Dose level (mg)							
	Statistic	10 mg	20 mg	40 mg	80 mg	100 mg	160 mg	200 mg	300 mg
Day 1		(*n* = 1)	(*n* = 1)	(*n* = 1)	(*n* = 3)	(*n* = 4)	(*n* = 3)	(*n* = 4)	(*n* = 3)
Cycle 1									
C_max_ (ng/mL)	G_mean_ (CV)	200	542	592	835 (10)	1810 (124)	1990 (12)	1490 (53)	2550 (32)
	Range				749–892	465–4600	1730–2190	890–2690	2100–3650
*T*_max_ (h)	Median	1.45	1.5	1.63	1.53	1.5	1.52	2.5	2
	Range				0.75–2.0	1.30–1.50	0.80–3.07	1.50–3.08	0.75–4.00
AUC_0-t_ (ng h/mL)	G_mean_ (CV)	485	1040	1920	3380 (10)	6280 (167)	6510 (15)	5860 (48)	11900 (24)
	Range				3100–3790	1140–14,800	5600–7560	3460–10,200	9470–15,100
**Day 3** **Cycle 1**		**(*n*** = **1)**	**(*n*** = **1)**	**(*n*** = **1)**	**(*n*** = **3)**	**(*n*** = **4)**	**(*n*** = **3)**	**(*n*** = **4)**	**(*n*** = **3)**
C_max_ (ng/mL)	G_mean_ (CV)	406	614	759	861 (21)	2750 (50)	1490 (10)	1620 (47)	2690 (18)
	Range				762–1090	1450–4280	1390–1660	1160–3100	2300–3260
*T*_max_ (h)	Median	0.5	1	1.5	1.5	1.61	1.52	2	1.02
	Range				1.05–2.0	0.50–2.02	1.52–2.05	1.48–2.00	1.00–2.00
AUC_0-t_ (ng.h/mL)	G_mean_ (CV)	868	1160	898	2970 (13)	8870 (38)	5100 (3)	6630 (55)	12,500 (17)
	Range				2660–3430	5380–12,800	4970–5280	4460–11900	10,500–14,700
**Day 18** **Cycle 1**		**(*n*** = **1)**	**(*n*** = **1)**	**(*n*** = **1)**	**(*n*** = **2)**	**(*n*** = **2)**	**(*n*** = **3)**	**(*n*** = **3)**	**(*n*** = **3)**
C_max_ (ng/mL)	G_mean_ (CV)	352	319	405	1090 (NC)	2330 (NC)	1350 (7)	1670 (50)	2880 (18)
	Range				1090–1100	2140–2530	1240–1420	1230–2880	2420–3450
*T*_max_ (h)	Median	1.58	1.02	1	1.23	1.9	1.5	2.03	1.5
	Range				0.47–2.0	1.45–2.35	0.50–2.00	1.00–3.50	1.00–2.07
AUC_0-t_ (ng.h/mL)	G_mean_ (CV)	817	718	1840	3500 (NC)	8100 (NC)	4930 (10)	5570 (55)	12,100 (6)
	Range				3440–3570	7000–9370	4400–5400	3900–10,000	11,400–12,900

*NC* not calculated (fewer than *n* = 3 values).

Plasma concentrations of ALM201 for each dose level were measured by validated liquid chromatographic tandem mass spectrometric (LC-MS/MS) methods. Pharmacokinetic parameters were calculated using Phoenix WinNonlin version 6.3 (Pharsight Corporation, USA). Summary statistics and plots of the data were generated in Microsoft Excel 2016. The 100 mg dose level is included in ascending dose order for ease of comparison but was dosed at the end of the study as cohort 8 on the recommendation of the cohort review committee after the maximum feasible dose was successfully reached. The number of patients (*n*) on each day of cycle 1 is shown. Supporting geometric mean plasma concentrations are summarised in Supplementary Table S1.

**Fig. 2:**
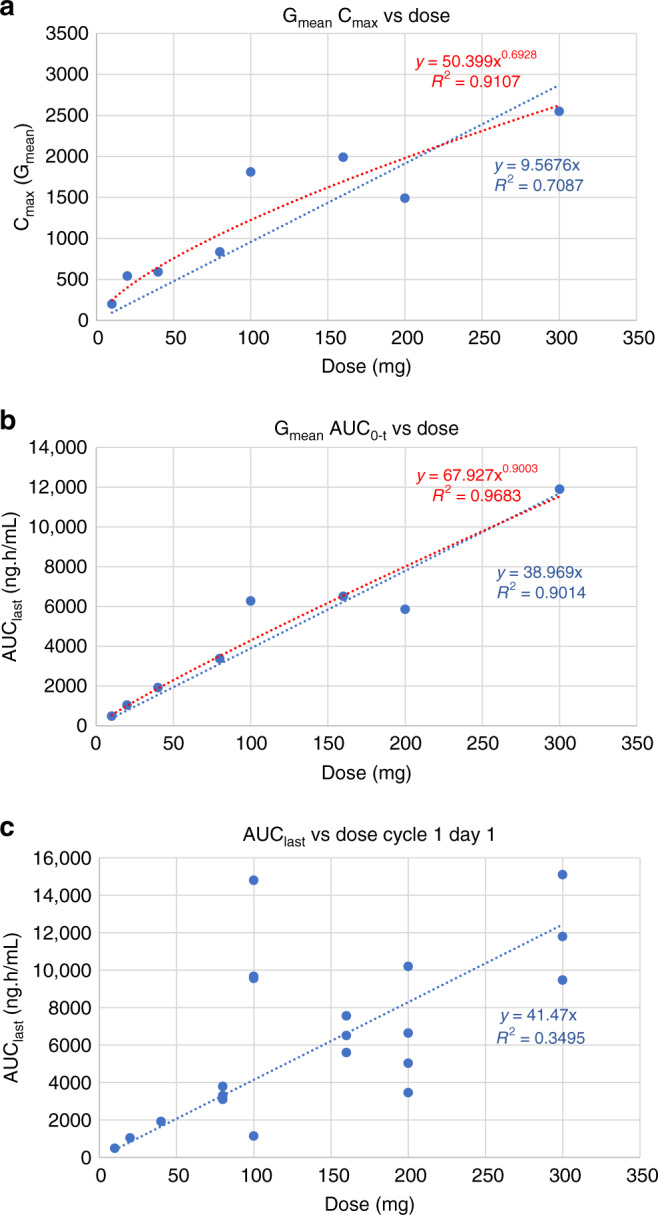
Pharmacokinetic profile of ALM201. **a** Dose proportionality plot to demonstrate pharmacokinetic data represented as dose versus mean maximal plasma concentration max (C_max_). **b** Dose proportionality plot to demonstrate pharmacokinetic data represented as dose versus mean area under the time–concentration curve (AUC). **c** Dose plot to demonstrate dose proportionality of pharmacokinetic data when represented as area under the time–concentration curve (AUC) versus dosing; data from individual patients are presented.

Following the first dose of ALM201, both measures of plasma exposure (i.e., geometric mean C_max_ and AUC_0-t_) tended to increase with dose (Fig. 2). However, based on linear regression analysis of the logarithmically transformed parameter and dose data, dose proportionality could not be formally concluded for either parameter. This was more marked for C_max_ than AUC_0-t,_ where slope parameter values were 0.67 and 0.86, respectively. Visual examination of the geometric mean C_max_ (Fig. 2a) and AUC (Fig. 2b) data showed no significant evidence of non-proportionality between doses of 10 and 160 mg. Less than proportional increases in systemic plasma exposure were observed at doses above 160 mg. There was less deviation from linearity when AUC was plotted against the dose (Fig. 2b).

### Assessment of accumulation following multiple doses

To assess the potential for drug accumulation over multiple weekday doses and multiple cycles, exposure parameters on different days and cycles were compared to the cycle 1 day 1 data. The derived pharmacokinetic parameters from multi-day data in cycle one are summarised in Table 4. Multi-cycle data (day 18 of cycles 2, 4, and 6) are summarised in Table 5. The geometric mean plasma concentration data are provided in Supplementary Tables S2 and S3. As observed following single dosing, absorption of the peptide was relatively rapid, with median *T*_max_ values across the dose groups ranging from 0.5 to 3.5 h and declining mono-exponentially over 5 h (or 7 h in the case of the 300 mg cohort). Exposure parameters typically showed low to moderate inter-patient variability (CVs of 10–50%). The average extent of accumulation over multiple dosing events was observed to be low (25–27%) when cycle 1, day 18 exposure data was compared to cycle 1, day 1 data. However, inter-patient variability in the data was high with the day 18:day 1 C_max_ ratios ranging from 0.59 to 4.60 (mean ± SD = 1.25 ± 0.97) and the AUC_0-t_ ratios ranging from 0.67 to 6.14 (mean ± SD = 1.27 ± 1.32); summary day 18:day-1 ratios derived from cycle 1 data in Table 4. This variability was greatly influenced by patient 03.012 (100 mg, cohort 8), where the day 1 data appear to be much lower than other patients in that cohort. Excluding patient 03.012 data reduced the mean ratios to 1.03 ± 0.39 (range: 0.59–1.76) for C_max_ and 0.95 ± 0.26 (range: 0.67–1.68) for AUC_0-t_, which are consistent with no accumulation. Moreover, C_max_ and AUC_0-t_ were relatively stable over multiple treatment cycles within patients (Table 5), suggesting little to no accumulation over multiple daily doses and treatment cycles. Additional evidence of a lack of accumulation over multiple dosing occasions was provided by pre-dose samples (collected on days 3 and 18 of cycle one and day 18 of cycles 2–6), all of which had non-quantifiable (<100 ng/mL) concentrations of ALM201.

**Table 5:** Pharmacokinetic parameters and summary statistics for ALM201 following subcutaneous administration on day 18 of cycles 2, 4 and 6 at each of the discrete dose levels of 10, 20, 40, 80, 100, 160, 200 and 300 mg.

Parameter	Summary	Dose level (mg)							
	Statistic	10 mg	20 mg	40 mg	80 mg	100 mg	160 mg	200 mg	300 mg
Day 18		(*n* = 1)	(*n* = 1)	(*n* = 1)	(*n* = 1)	(*n* = 1)	(*n* = 1)	(*n* = 2)	(*n* = 3)
Cycle 2									
C_max_ (ng/mL)	G_mean_ (CV)	288	394	583	849	1790	1870	1890 (NC)	2780 (40)
	Range							1280–2790	2110–4310
*T*_max_ (h)	Median	2	1	1.03	3.5	1.02	2	1.54	2
	Range							1.50–1.58	1.48–2.13
AUC_0-t_ (ng h/mL)	G_mean_ (CV)	702	1020	1950	3230	5930	5710	5680 (NC)	10,400 (45)
	Range							3810–8480	7290–16,700
**Day 18** **Cycle 4**		**(*n*** = **1)**						**(*n*** = **2)**	**(*n*** = **1)**
C_max_ (ng/mL)	G_mean_ (CV)	193	NS	NS	NS	NS	NS	1680 (NC)	2140
	Range							1100–2580	
*T*_max_ (h)	Median	1.53	NS	NS	NS	NS	NS	1.57	1.12
	Range							1.00–2.13	
AUC_0-t_ (ng h/mL)	G_mean_ (CV)	476	NS	NS	NS	NS	NS	6110 (NC)	8600
	Range							4150–9010	
**Day 18** **Cycle 6**		**(*n*** = **1)**							**(*n*** = **1)**
C_max_ (ng/mL)	G_mean_ (CV)	470	NS	NS	NS	NS	NS	NS	6830
	Range								
*T*_max_ (h)	Median	1.85	NS	NS	NS	NS	NS	NS	2.02
	Range								
AUC_0-t_ (ng h/mL)	G_mean_ (CV)	1040	NS	NS	NS	NS	NS	NS	26,700
	Range								

*NC* not calculated (fewer than *n* = 3 values).

Plasma concentrations of ALM201 for each dose level were measured by validated liquid chromatographic tandem mass spectrometric (LC-MS/MS) methods. Pharmacokinetic parameters were calculated using Phoenix WinNonlin version 6.3 (Pharsight Corporation, USA). Summary statistics and plots of the data were generated in Microsoft Excel 2016. The 100 mg dose level is included in ascending dose order for ease of comparison but was dosed at the end of the study as cohort 8 on the recommendation of the cohort review committee after the maximum feasible dose was successfully reached. The number of patients (*n*) on day 18 of each dose cycle is shown. Supporting geometric mean plasma concentrations are summarised in Supplementary Tables S2 and S3.

### Assessment of elimination of ALM201 in urine

In the vast majority of patients, the recovery of ALM201 in urine was lower than the assay limit of quantification (LLOQ = 100 ng/mL). Where positive concentrations were detected, the amount of ALM201 excreted in the urine was very low and represented 0.01% to 0.18% of the administered dose (mean (±SD) = 0.074 (0.069)). The urine recovery data are summarised in Supplementary Table S4.

### Assessment of anti-tumour efficacy outcomes

**Fig. 3:**
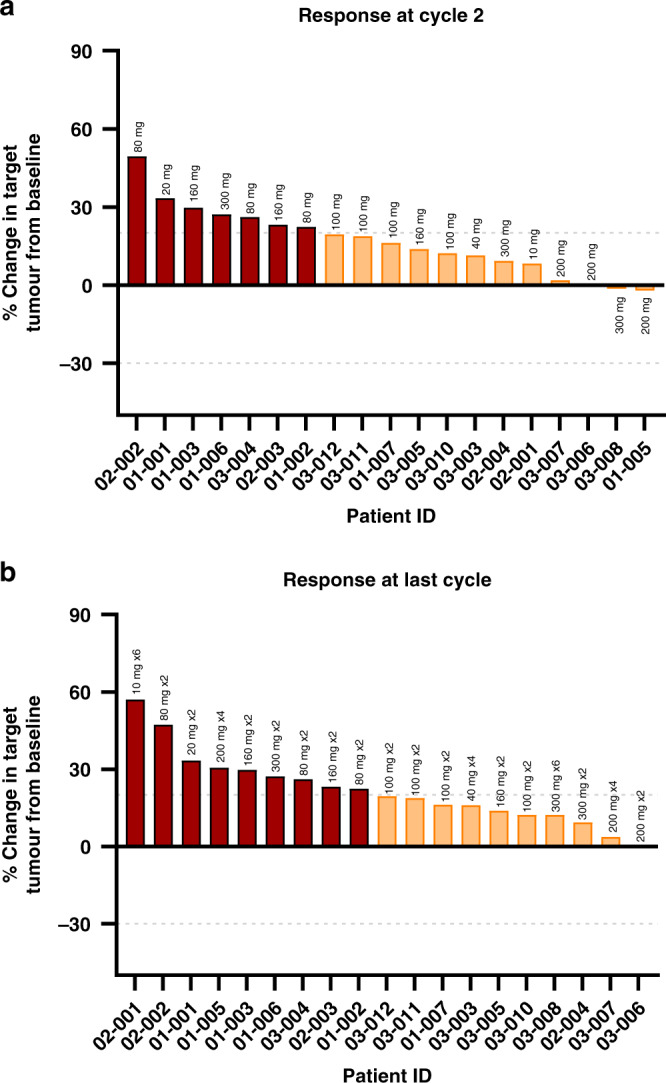
Tumour responses plotted as a percentage change in the sum of target tumour lesions from baseline and presented as waterfall plots. Patient IDs are plotted along the *x* axis and ranked (left to right) according to the anti-tumour response. Tumour measurements of evaluable target lesions were estimated from CT scans, and the longest diameter of each target lesion was summed. Data for patient 01-004 (Cohort 4, 200 mg) was only obtained at baseline and is not shown. Horizontal grey dashed lines represent thresholds of 20 and −30. Above 20 = progressive disease (red bars), between which = stable disease (orange bars) and below −30 = partial or complete responses (green bars). **a** specifically shows tumour responses in individual patients after two cycles of ALM201 treatment, and **b** shows responses after the last cycle. The dose level and the number of cycles achieved (xN) are shown above each column. In both graphs, the data for patient 03-006 (Cohort 6, 200 mg) is a fraction of an integer and is too small to visualise next to the graph axis.

When the disease control rate was used to measure efficacy, 35% (7/20) of the patients had stable disease for up to four cycles before progression (see Table 6). A summary of clinical benefit by RECIST Version 1.1 from patients with the evaluable disease was generated (Fig. 3). At the final study tumour assessment, target lesions increased in size in all 20 patients compared to the baseline or nadir value assessment; in 9 of these patients, this change was sufficient to constitute disease progression. Some target lesions were noted as unchanged or improved in nine patients during the study compared to the baseline assessment, although these subsequently worsened. For non-target lesions, new lesions were observed in five patients. No partial or complete responses were reported in any of the patients during the study. Overall, 12 patients (60%) had disease progression, and 7 patients each (35% [95% CI 15.4, 59.2]) had the best response of stable disease (SD) and achieved disease control. Three patients had SD until the end of cycle 4.

**Table 6:** Summary of the number of treatment cycles and responses for each patient in each dose cohort.

	Cohort 1, 10 mg (*n* = 1)	Cohort 2, 20 mg (*n* = 1)	Cohort 3, 40 mg (*n* = 1)	Cohort 4, 80 mg (*n* = 3)	Cohort 5, 160 mg (*n* = 3)	Cohort 6, 200 mg (*n* = 4)	Cohort 7, 300 mg (*n* = 3)	Cohort 8, 100 mg (*n* = 4)	Overall (*n* = 20)
Complete response (CR)									
* n* (%)	0	0	0	0	0	0	0	0	0
95% CI	(0.0, 97.5)	(0.0, 97.5)	(0.0, 97.5)	(0.0, 70.8)	(0.0, 70.8)	(0.0, 60.2)	(0.0, 70.8)	(0.0, 60.2)	(0.0, 16.8)
Partial response (PR)									
* n* (%)	0	0	0	0	0	0	0	0	0
95% CI	(0.0, 97.5)	(0.0, 97.5)	(0.0, 97.5)	(0.0, 70.8)	(0.0, 70.8)	(0.0, 60.2)	(0.0, 70.8)	(0.0, 60.2)	(0.0, 16.8)
Overall response rate (CR + PR)									
* n* (%)	0	0	0	0	0	0	0	0	0
95% CI	(0.0, 97.5)	(0.0, 97.5)	(0.0, 97.5)	(0.0, 70.8)	(0.0, 70.8)	(0.0, 60.2)	(0.0, 70.8)	(0.0, 60.2)	(0.0, 16.8)
Stable disease (SD)									
* n* (%)	1 (100%)	0	1 (100%)	0	0	2 (50.0)	2 (66.7)	1 (25.0)	7 (35.0)
95% CI	(2.5, 100)	(0.0, 97.5)	(2.5, 100)	(0.0, 70.8)	(0.0, 70.8)	(6.8, 93.2)	(9.4, 99.2)	(0.6, 80.6)	(15.4, 59.2)
Disease control rate (CR + PR + SD)									
* n* (%)	1 (100%)	0	1 (100%)	0	0	2 (50.0)	2 (66.7)	1 (25.0)	7 (35.0)
95% CI	(2.5, 100)	(0.0, 97.5)	(2.5, 100)	(0.0, 70.8)	(0.0, 70.8)	(6.8, 93.2)	(9.4, 99.2)	(0.6, 80.6)	(15.4, 59.2)
Progressive disease (PD)									
* n* (%)	0	1 (100)	0	3 (100)	3 (100)	1 (25)	1 (33.3)	3 (75.0)	1 2 (60.0)
95% CI	(0.0, 97.5)	(2.5, 100)	(0.0, 97.5)	(29.2, 100)	(29.2, 100)	(0.6, 80.6)	(0.8, 90.6)	(19.4, 99.4)	(36.1, 80.9)
Not evaluable (NE + NA)									
* n* (%)	0	0	0	0	0	1 (25.0)	0	0	1 (5.0)
95% CI	(0.0, 97.5)	(0.0, 97.5)	(0.0, 97.5)	(0.0, 70.8)	(0.0, 70.8)	(0.6, 80.6)	(0.0, 70.8)	(0.0, 60.2)	(0.1, 24.9)

*CR* complete response, *PR* partial response, *SD* stable disease, *NE* not evaluable, *NA* not applicable.

The disease control rate was used to assess anti-tumour efficacy outcomes.

## Discussion

Here, we report the results of an open-label, Phase I first-in-human study of ALM201 in unselected patients with advanced solid cancers. ALM201, when administered systemically by subcutaneous injection, was very well-tolerated. Furthermore, no DLTs were documented, and the MTD was not reached, but an MFD was based on a pre-planned limit of 3 × 1.0 mL SC injections daily. This lack of toxicity aligned with our expectations of a non-immunogenic, small 23-amino acid peptide derived from an endogenous natural human protein.

In addition, the pharmacokinetic profile of ALM201 was better than suggested by our allometric scaling and computer modelling predictions. Although dose proportionality could not be formally concluded, there was no substantial evidence of non-proportionality across the lower dose ranges (10–160 mg), with slightly less than proportional increases in systemic plasma exposure observed above 160 mg. When the excretion of the parent peptide in the urine was considered a preliminary picture of drug disposition, barely any parent peptide was excreted in the urine of patients over the collection period. Altogether, this Phase I patient dataset provides an important, albeit initial assessment, of drug disposition and elimination in the clinical setting for this novel therapeutic peptide.

Furthermore, ALM201, demonstrated good (>60% tumour growth inhibition) anti-tumour responses in ovarian patient-derived xenograft models. Computer modelling demonstrated that this dose was predicted to align with the PK obtained from patients dosed with 100 mg ALM201. The plasma AUC exposure observed in patients dosed at 100 mg or above was higher than the efficacious dose (3 mg/kg) in pre-clinical models, suggesting that efficacious plasma exposures were achievable in patients at doses of 100 mg and above.

Anti-tumour activity was only stipulated as a secondary study objective in this trial. Although no partial or complete responses were observed in this initial safety study, we identified possible signs of anti-tumour activity in three patients. It is important to highlight that this was an all-comer Phase I study, and the lack of efficacy may be due to (1) the fact that patients were heavily pre-treated, prior to recruitment, and (2) there was no biomarker stratified expansion cohorts. Further clinical development in larger cohorts of patients would benefit from discovering and applying appropriate and robust patient-selection biomarkers.

Indeed, one of the major limitations that have been encountered in deploying anti-angiogenic agents to treat ovarian and other cancer types has been the lack of validated biomarkers that can guide therapeutic use and improve clinical outcomes of these drugs [22]. This may also expose patients who would not benefit from this class of agents to unnecessary toxicities, not to mention significant increases in healthcare costs and resources. Therefore, an additional exploratory objective of this study was to define a biologically active dose of ALM201 by using scientifically rational biomarkers. FKBPL itself, the endogenous protein from which ALM201 was derived, has been proposed as a biomarker for response in breast cancer [23], suggesting that certain functional aspects of FKBPL may provide an overall survival benefit to certain patients. However, it is not entirely clear if ALM201 would be sufficient to restore a survival benefit in these patients. While it retains the anti-angiogenic activity of FKBPL, it does not necessarily mimic its other anticancer roles [13].

To better understand the underlying biological effects of the peptide, recent work using a 3D co-culture model of endothelial tubule growth and cancer cell migration suggests that ALM201, like its sister peptide AD-01, could have a direct impact on the attachment and migration of clusters of cancer cells along branched endothelial tubule networks [24]. This implies that ALM201 might also potentially interfere with the migration of cancer cells along blood vessels, thereby disrupting the complex microenvironment of solid tumours. Whether this translates to a reduction in metastatic potential or local invasion remains to be evaluated. Interestingly, the inhibitory effect of the peptide was observed to be most striking in spheroids that over-expressed *ERBB2* (*HER2/neu*), which suggests that tumours overexpressing *HER2* might be more amenable to therapeutic intervention. In addition, in these experiments, transcripts of CD44 were increased when the cells were cultured as spheroids, but this did not appear to translate to increased levels of CD44 protein [24]. Whether or not these findings have any bearing on patients remains to be investigated.

The influence of CD44, the putative target for ALM201, on biological processes appears to be less than clear-cut. For example, the migration and survival of glioblastoma cells are dependent on CD44 but follow a biphasic response profile in both mice and humans [25]. However, whether this biphasic response is limited to glioblastoma or occurs across multiple cancer types is unknown.

A recent publication by Annett and colleagues has highlighted, alongside its anti-angiogenic activity, a potential role for ALM201 in targeting cancer stem cells (CSCs) in ovarian cancer cell lines [26]. It remains to be seen if this anti-CSC activity would translate to the clinical setting. However, it aligns with the properties of a novel therapeutic agent capable of targeting pleiotropic biological processes within the complex tumour microenvironment of advanced solid tumours.

Despite a lack of clarity around the biological mechanism of action and patient biomarkers, when taken together, the excellent safety profile, and good systemic exposure in this initial study suggest that ALM201 could be of therapeutic benefit to patients. ALM201 could either be provided as a single agent in the maintenance setting or combined with other drugs to target angiogenesis in solid tumours. Alternatively, ALM201 could be used in combination with radiotherapy, where the targeted radiotherapy could conceivably be used to induce death of the resilient, well-perfused viable rim of tumour cells following intra-tumoural vasculature ablation [27]. Such an approach may have therapeutic merit and align with the clinical development need for additional new drug-radiotherapy combinations [28]. However, further translational biology and clinical development activities to support improved patient-selection strategies will be required before this new therapeutic peptide can be progressed further in clinical trials.

## Supplementary information

Geometric mean plasma concentrations of ALM201 following subcutaneous administration on Day 1 of treatment cycle 1 at doses of 10, 20, 40, 80, 100, 160, 200 or 300 mg

Geometric mean plasma concentrations of ALM201 following subcutaneous administration on Day 3 and Day 18 of treatment cycle 1 at doses of 10, 20, 40, 80, 100, 160, 200 or 300 mg

Geometric mean plasma concentrations of ALM201 following subcutaneous administration on Day 18 of treatment cycle 2, 4 and 6 at doses of 10, 20, 40, 80, 100, 160, 200 or 300 mg

Urinary recovery data for ALM201 following subcutaneous administration on Day 1 of cycle 1 of treatment at doses of 10, 20, 40, 80, 100, 160, 200 or 300 mg

Overview of the ALM201 Clinical Dose Escalation Scheme

Reproducibility checklist

ALM201 protocol

## Data Availability

The data generated and/or analysed in this study are available from the corresponding author upon request.
