# Supplementary material for: A first-in-human Phase I dose-escalation trial of the novel therapeutic peptide, ALM201, demonstrates a favourable safety profile in unselected patients with ovarian cancer and other advanced solid tumours
Source: Br J Cancer. 2022 May 14;127(1):92–101. doi: 10.1038/s41416-022-01780-z (PMC9276671; doi:10.1038/s41416-022-01780-z)
Supplement: Supplementary file 1 — Geometric mean plasma concentrations of ALM201 following subcutaneous administration on Day 1 of treatment cycle 1 at doses of 10, 20, 40, 80, 100, 160, 200 or 300 mg [file 41416_2022_1780_MOESM1_ESM.pdf]

Table S1. **Geometric mean plasma concentrations of ALM201 following subcutaneous administration on Day 1 of treatment cycle 1 at doses of 10, 20, 40, 80, 100, 160, 200 or 300 mg**

**Time after Dosing (h)**	**ALM201 Plasma Concentration (ng/mL) (Geometric mean (CV%) and range)**							
	**10 mg**	**20 mg**	**40 mg**	**80 mg**	**100 mg**	**160 mg**	**200 mg**	**300 mg**
**Day 1 Cycle 1**	(n = 1)	(n = 1)	(n = 1)	(n = 3)	(n = 4)	(n = 3)	(n = 4)	(n = 3)
Pre-dose	NQ	NQ	NQ	NQ	NQ	NQ	NQ	NQ
0.25	106	136	316	292 (73)<br>192 – 620	492 (87)<br>160 – 801	833 (47)<br>507 – 1190	479 (84)<br>250 – 1360	1050 (130)<br>491 – 3240
0.75	150	368	556	700 (16)<br>585 – 784	1220 (99)<br>377 – 2650	1570 (22)<br>1220 – 1840	1040 (61)<br>492 – 1900	1970 (33)<br>1380 – 2550
1.5	200	542	592	800 (14)<br>678 – 872	1810 (124)<br>465 – 4600	1670 (19)<br>1490 – 2080	1240 (41)<br>802 - 2090	2250 (38)<br>1710 – 3420
2	199	417	540	727 (32)<br>507 – 892	1590 (126)<br>398 – 3910	1480 (17)<br>1290 – 1790	1370 (52)<br>802 – 2270	2310 (41)<br>1820 – 3650
3	153	224	368	689 (18)<br>572 – 824	1150 (272)<br>131 – 2930	1280 (50)<br>903 – 2190	1360 (53)<br>890 – 2690	2120 (19)<br>1840 – 2630
4	NQ	NQ	223	556 (16)<br>486 - 664	922 (282)<br>101 – 2300	866 (30)<br>708 - 1210	1050 (29)<br>789 – 1380	1600 (32)<br>1130 – 2100
5	NQ	NQ	158	359 (40)<br>233 – 480	664 (198)*<br>NQ – 1330	510 (20)<br>463 – 640	620 (56)<br>306 – 1070	1280 (26)<br>980 – 1630
6	NQ	NQ	NQ	248 (32)<br>194 – 350	560 (166)*<br>NQ - 1030	283 (10)<br>252 – 304	518 (55)<br>274 – 957	901 (28)<br>658 -1090
7	NS	NS	NS	NS	NS	NS	NS	703 (45)<br>502 – 1140
8	NS	NS	NS	NS	NS	NS	NS	420 (55)<br>282 – 753
22	NQ	NQ	NQ	NQ	NQ	NQ	NQ	NQ

NS = no sample received; NQ = not quantifiable (less than assay limit of quantification of 100 ng/mL); * NQ = value substituted with assay limit of quantification (100 ng/mL) in calculation of geometric mean
