# Supplementary material for: A first-in-human Phase I dose-escalation trial of the novel therapeutic peptide, ALM201, demonstrates a favourable safety profile in unselected patients with ovarian cancer and other advanced solid tumours
Source: Br J Cancer. 2022 May 14;127(1):92–101. doi: 10.1038/s41416-022-01780-z (PMC9276671; doi:10.1038/s41416-022-01780-z)
Supplement: Supplementary file 2 — Geometric mean plasma concentrations of ALM201 following subcutaneous administration on Day 3 and Day 18 of treatment cycle 1 at doses of 10, 20, 40, 80, 100, 160, 200 or 300 mg [file 41416_2022_1780_MOESM2_ESM.pdf]

Table S2. **Geometric mean plasma concentrations of ALM201 following subcutaneous administration on Day 3 and Day 18 of treatment cycle 1 at doses of 10, 20, 40, 80, 100, 160, 200 or 300 mg**

Time after Dosing (h)	ALM201 Plasma Concentration (ng/ml) (Geometric mean (CV%) and range)							
	**10 mg**	**20 mg**	**40 mg**	**80 mg**	**100 mg**	**160 mg**	**200 mg**	**300 mg**
**Day 3 Cycle 1**	(n = 1)	(n = 1)	(n = 1)	(n = 3)	(n = 4)	(n = 3)	(n = 4)	(n = 3)
Pre-dose	NQ	NQ	NQ	NQ	NQ	NQ	NQ	NQ
0.5	406	210	264	578 (58)<br>321 – 920	1730 (38)<br>1180 – 2560	956 (8)<br>875 – 1030	1090 (81)<br>637 – 3000	1970 (30)<br>1620- 2770
1	325	614	444	684 (40)<br>445 – 935	2100 (48)<br>1250 – 3670	1270 (8)<br>1160 – 1350	1410 (70)*<br>818 - 2830	2590 (24)<br>2050 – 3260
1.5	294	532	759	827 (25)<br>679 – 1090	2530 (56)<br>1430 - 4280	1460 (11)<br>1350 – 1660	1320 (69)<br>703 – 3100	1950 (NC)<br>1650 – 2300
2	242	377	656	826 (16)<br>742 – 996	2400 (50)<br>1450 – 3660	1350 (10)<br>1200 – 1430	1540 (37)<br>1160 – 2590	2450 (NC)<br>2300 – 2620
3.5	127	134	NS	524 (28)<br>399 – 697	1690 (32)<br>1080 – 2120	926 (2)<br>909 – 935	1250 (49)<br>904 - 2480	NS
4.5	NS	NS	NS	NS	NS	NS	NS	1740 (29)<br>1260 – 2130
5	NQ	NQ	NS	325 (36)<br>241 – 477	928 (26)<br>699 – 1210	613 (10)<br>544 – 651	958 (43)<br>681 – 1650	NS
7	NS	NS	NS	NS	NS	NS	NS	852 (48)<br>526 – 1290
**Day 18 Cycle 1**	(n = 1)	(n = 1)	(n = 1)	(n = 2)	(n = 2)	(n = 3)	(n = 3)	(n = 3)
Pre-dose	NQ	NQ	NQ	NQ	NQ	NQ	NQ	NQ
0.5	188	194	348	769 (NC)<br>538 - 1100	1890 (NC)<br>1450 – 2460	940 (41)<br>631 – 1400	811 (105)<br>353 – 1970	2290 (12)<br>2120 – 2640
1	283	319	405	946 (NC)<br>814 – 1100	1840 (NC)<br>1830 – 1850	1150 (16)<br>964 – 1290	1260 (82)<br>625 – 2630	2410 (3)<br>2330 – 2490
1.5	352	314	397	884 (NC)<br>834 – 937	2210 (NC)<br>2140 – 2280	1280 (4)<br>1240 - 1330	1670 (NC)<br>1050 – 2670	2600 (26)<br>2090 – 3450
2	325	224	368	1065 (NC)<br>1040 – 1090	2130 (NC)<br>1790 – 2530	1200 (16)<br>1050 – 1420	1580 (NC)<br>868 – 2880	2520 (21)<br>1980 – 2870
3.5	113	113	377	545 (NC)<br>460 – 646	1490 (NC)<br>1310 – 1700	995 (9)<br>902 – 1080	1130 (45)<br>693 – 1580	NS
4.5	NS	NS	NS	NS	NS	NS	NS	1370 (25)<br>1170 – 1820
5	NQ	NQ	113	407 (NC)<br>328 – 504	961 (NC)<br>776 - 1190	577 (16)<br>488 – 674	890 (34)<br>619 -1170	NS
7	NS	NS	NS	NS	NS	NS	NS	767 (80)<br>418 – 1650

NS = no sample received; NC = not calculated (n = 2 values); NQ = not quantifiable (less than assay limit of quantification of 100 ng/mL); *n = 3
