# Supplementary material for: A first-in-human Phase I dose-escalation trial of the novel therapeutic peptide, ALM201, demonstrates a favourable safety profile in unselected patients with ovarian cancer and other advanced solid tumours
Source: Br J Cancer. 2022 May 14;127(1):92–101. doi: 10.1038/s41416-022-01780-z (PMC9276671; doi:10.1038/s41416-022-01780-z)
Supplement: Supplementary file 3 — Geometric mean plasma concentrations of ALM201 following subcutaneous administration on Day 18 of treatment cycle 2, 4 and 6 at doses of 10, 20, 40, 80, 100, 160, 200 or 300 mg [file 41416_2022_1780_MOESM3_ESM.pdf]

Table S3. **Geometric mean plasma concentrations of ALM201 following subcutaneous administration on Day 18 of treatment cycle 2, 4 and 6 at doses of 10, 20, 40, 80, 100, 160, 200 or 300 mg**

Time after Dosing (h)	ALM201 Plasma Concentration (ng/mL) (Geometric mean (CV%) and range)							
	**10 mg**	**20 mg**	**40 mg**	**80 mg**	**100 mg**	**160 mg**	**200 mg**	**300 mg**
**Day 18 Cycle 2**	(n = 1)	(n = 1)	(n = 1)	(n = 1)	(n = 1)	(n = 1	(n = 2)	(n = 3)
Pre-dose	NQ	NQ	NQ	NQ	NQ	NQ	NQ	NQ
0.5	158	279	384	394	1060	1380	1210 (NC)<br>908 – 1620	1990 (42)<br>1460 – 3150
1	193	394	583	808	1790	1380	1470 (NC)<br>1010 – 2150	2280 (39)<br>1810 – 3510
1.5	242	352	565	659	1710	1650	1890 (NC)<br>1280 – 2790	2380 (55)<br>1750 – 4130
2	288	386	571	639	1570	1870	1360 (NC)<br>1240 - 1500	2540 (23)<br>2110 – 3290
3.5	161	162	347	849	1180	870	911 (NC)<br>486 – 1710	720 (206)*<br>169 – 1970
5	NQ	NQ	228	505	535	436	690 (NC)<br>353 – 1350	485 (58) **<br>282 - 825
**Day 18 Cycle 4**	(n = 1)						(n = 2)	(n = 1)
Pre-dose	NQ	NS	NS	NS	NS	NS	NQ	NQ
0.5	NQ	NS	NS	NS	NS	NS	1080 (NC)<br>479 – 2430	1670
1	176	NS	NS	NS	NS	NS	1410 (NC)<br>1100 - 1800	2140
1.5	193	NS	NS	NS	NS	NS	1150 (NC)<br>734 – 1810	1990
2	171	NS	NS	NS	NS	NS	1500 (NC)<br>876 – 2580	1990
3.5	157	NS	NS	NS	NS	NS	1310 (NC)<br>970 -1760	854*
5	NQ	NQ	228	505	535	436	975 (NC)<br>889 – 1070	387**
**Day 18 Cycle 6**	(n = 1)							(n = 1)
Pre-dose	NQ	NS	NS	NS	NS	NS	NS	NQ
0.5	171	NS	NS	NS	NS	NS	NS	3150
1	NS	NS	NS	NS	NS	NS	NS	4430
1.5	470	NS	NS	NS	NS	NS	NS	5240
2	403	NS	NS	NS	NS	NS	NS	6830
3.5	179	NS	NS	NS	NS	NS	NS	3600*
5	NS	NS	NS	NS	NS	NS	NS	693**

NS = no sample received; NC = not calculated (n = 2 values); NQ = not quantifiable (less than assay limit of quantification of 100 ng/mL); *Samples collected at 4.5 hours; **Samples collected at 7.0 hours
