# Supplementary material for: A first-in-human Phase I dose-escalation trial of the novel therapeutic peptide, ALM201, demonstrates a favourable safety profile in unselected patients with ovarian cancer and other advanced solid tumours
Source: Br J Cancer. 2022 May 14;127(1):92–101. doi: 10.1038/s41416-022-01780-z (PMC9276671; doi:10.1038/s41416-022-01780-z)
Supplement: Supplementary file 4 — Urinary recovery data for ALM201 following subcutaneous administration on Day 1 of cycle 1 of treatment at doses of 10, 20, 40, 80, 100, 160, 200 or 300 mg [file 41416_2022_1780_MOESM4_ESM.pdf]

Table 4. Derived pharmacokinetic parameters for ALM201 following subcutaneous administration on Days 1, 3 and 18 of treatment cycle 1 at doses of 10, 20, 40, 80, 100, 160, 200 or 300 mg

Parameter	Summary Statistic	Dose Level (mg)							
		10 mg	20 mg	40 mg	80 mg	100 mg	160 mg	200 mg	300 mg
**Day 1 Cycle 1**		(n = 1)	(n = 1)	(n = 1)	(n = 3)	(n = 4)	(n = 3)	(n = 4)	(n = 3)
C_{max} (ng/mL)	Gmean (CV)<br>Range	200	542	592	835 (10)<br>749 – 892	1810 (124)<br>465 – 4600	1990 (12)<br>1730 – 2190	1490 (53)<br>890 -2690	2550 (32)<br>2100 - 3650
t_{max} (h)	Median<br>Range	1.45	1.5	1.63	1.53<br>0.75 – 2.0	1.5<br>1.30 – 1.50	1.52<br>0.80 – 3.07	2.5<br>1.50 – 3.08	2<br>0.75 – 4.00
AUC_{0-t} (ng.h/mL)	Gmean (CV)<br>Range	485	1040	1920	3380 (10)<br>3100 - 3790	6280 (167)<br>1140 – 14800	6510 (15)<br>5600 - 7560	5860 (48)<br>3460 – 10200	11900 (24)<br>9470 – 15100
**Day 3 Cycle 1**		(n = 1)	(n = 1)	(n = 1)	(n = 3)	(n = 4)	(n = 3)	(n = 4)	(n = 3)
C_{max} (ng/mL)	Gmean (CV)<br>Range	406	614	759	861 (21)<br>762 – 1090	2750 (50)<br>1450 - 4280	1490 (10)<br>1390 – 1660	1620 (47)<br>1160 – 3100	2690 (18)<br>2300 – 3260
t_{max} (h)	Median<br>Range	0.5	1	1.5	1.5<br>1.05 – 2.0	1.61<br>0.50 – 2.02	1.52<br>1.52 – 2.05	2<br>1.48 – 2.00	1.02<br>1.00 – 2.00
AUC_{0-t} (ng.h/mL)	Gmean (CV)<br>Range	868	1160	898	2970 (13)<br>2660 - 3430	8870 (38)<br>5380 - 12800	5100 (3)<br>4970 – 5280	6630 (55)<br>4460 – 11900	12500 (17)<br>10500 – 14700
**Day 18 Cycle 1**		(n = 1)	(n = 1)	(n = 1)	(n = 2)	(n = 2)	(n = 3)	(n = 3)	(n = 3)
C_{max} (ng/mL)	Gmean (CV)<br>Range	352	319	405	1090 (NC)<br>1090 - 1100	2330 (NC)<br>2140 – 2530	1350 (7)<br>1240 – 1420	1670 (50)<br>1230 – 2880	2880 (18)<br>2420 – 3450
t_{max} (h)	Median<br>Range	1.58	1.02	1	1.23<br>0.47 – 2.0	1.9<br>1.45 – 2.35	1.5<br>0.50 – 2.00	2.03<br>1.00 – 3.50	1.5<br>1.00 – 2.07
AUC_{0-t} (ng.h/mL)	Gmean (CV)<br>Range	817	718	1840	3500 (NC)<br>3440 - 3570	8100 (NC)<br>7000 - 9370	4930 (10)<br>4400 - 5400	5570 (55)<br>3900 - 10000	12100 (6)<br>11400 – 12900

NC = not calculated (fewer than n = 3 values)
