# Supplementary material for: A first-in-human Phase I dose-escalation trial of the novel therapeutic peptide, ALM201, demonstrates a favourable safety profile in unselected patients with ovarian cancer and other advanced solid tumours
Source: Br J Cancer. 2022 May 14;127(1):92–101. doi: 10.1038/s41416-022-01780-z (PMC9276671; doi:10.1038/s41416-022-01780-z)
Supplement: Supplementary file 5 — Overview of the ALM201 Clinical Dose Escalation Scheme [file 41416_2022_1780_MOESM5_ESM.pptx]

## Slide 1
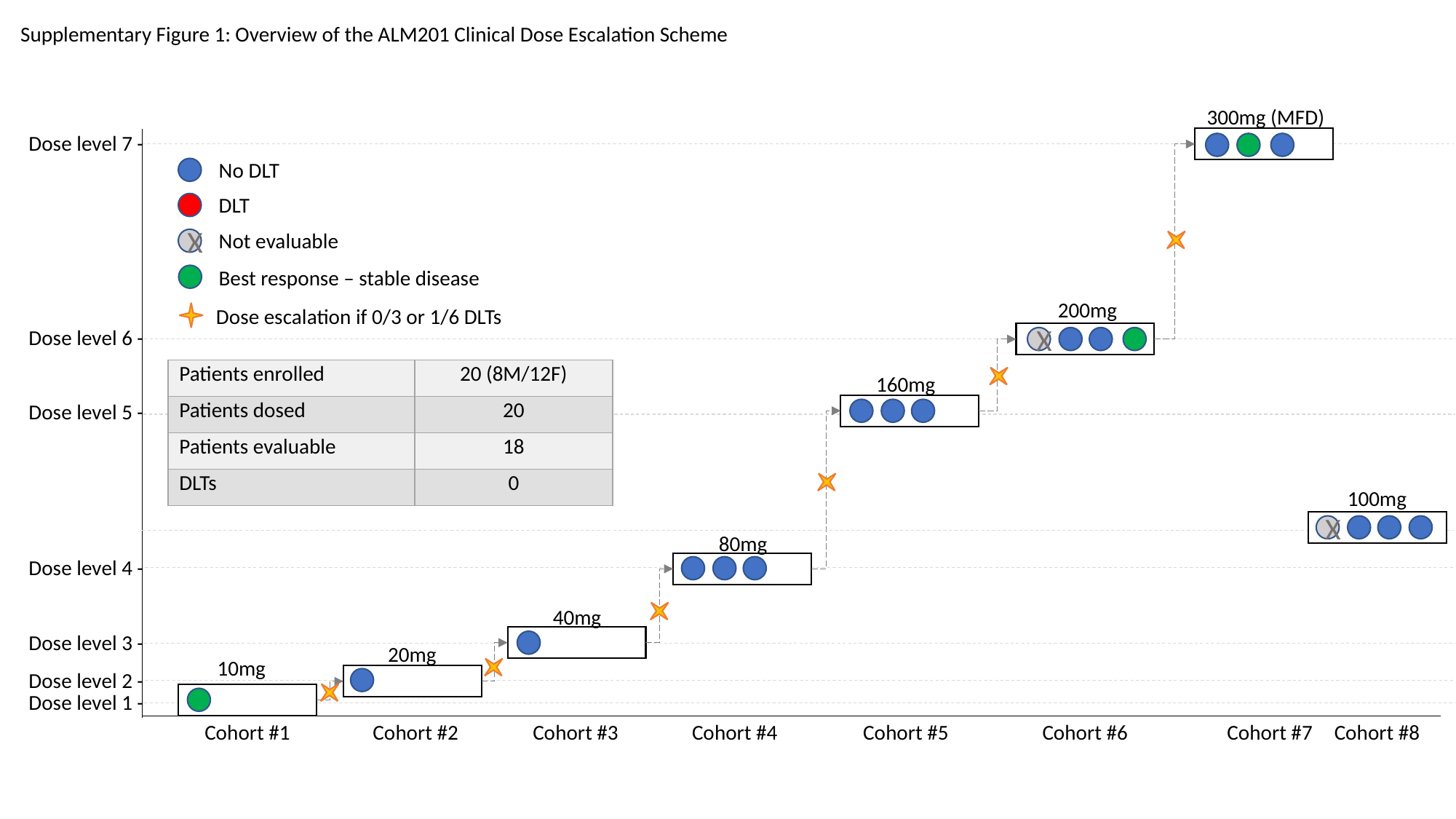

Supplementary Figure 1: Overview of the ALM201 Clinical Dose Escalation Scheme
300mg (MFD)
Dose level 7 -
No DLT
DLT
Not evaluable
X
Best response – stable disease
200mg
Dose escalation if 0/3 or 1/6 DLTs
Dose level 6 -
X
| Patients enrolled | 20 (8M/12F) |
| --- | --- |
| Patients dosed | 20 |
| Patients evaluable | 18 |
| DLTs | 0 |
160mg
Dose level 5 -
100mg
X
80mg
Dose level 4 -
40mg
Dose level 3 -
20mg
10mg
Dose level 2 -
Dose level 1 -
Cohort #1
Cohort #2
Cohort #3
Cohort #4
Cohort #5
Cohort #6
Cohort #7
Cohort #8
