# Supplementary material for: A first-in-human Phase I dose-escalation trial of the novel therapeutic peptide, ALM201, demonstrates a favourable safety profile in unselected patients with ovarian cancer and other advanced solid tumours
Source: Br J Cancer. 2022 May 14;127(1):92–101. doi: 10.1038/s41416-022-01780-z (PMC9276671; doi:10.1038/s41416-022-01780-z)
Supplement: Supplementary file 7 — ALM201 protocol [file 41416_2022_1780_MOESM7_ESM.pdf]

CLINICAL STUDY PROTOCOL

A phase I open-label multicentre dose-escalation study of subcutaneous ALM201 in patients with advanced ovarian cancer and other solid tumours

Study Protocol:	ALM201/0001
Study Drug(s):	ALM201
Version No:	F2.0
Version Date:	24 SEP 14
EudraCT No:	2014-001175-31
Sponsor:	Almac Discovery<br>Almac House<br>20 Seagoe Industrial Estate<br>Craigavon<br>BT63 5QD<br>UK<br><br>Phone: +44 2838332200<br>Fax: +44 2838332299

The information contained in this document, especially unpublished data, is confidential communication from Almac. Acceptance of this document constitutes the agreement of the recipient that this information will not be disclosed to others without written authorisation from Almac, except to the extent necessary for Ethics Committee/Institutional Review Board procedures and to obtain written informed consent from those persons to whom test drug may be administered.

Signatures/Protocol Approval and Release

We, the undersigned, have read this protocol and agree that they contain all the necessary information required for the conduct of the study.

For Almac Discovery (Almac):

Colin Hayburn, LLB
Executive Director, General Counsel & Company Secretary

Signature [illegible]

Date 25-September-2014

Stephen Barr, PhD
President and Managing Director

Signature [illegible]

Date 25 Sep 2014

For CRO:

Coordinating Author:
Irene Gow
Ockham Oncology

Signature ____________________

Date ____________________

Signatures/Protocol Approval and Release

We, the undersigned, have read this protocol and agree that they contain all the necessary information required for the conduct of the study.

For Almac Discovery (Almac):

Colin Hayburn, LLB
Executive Director, General Counsel
& Company Secretary

Signature ______________________________

Date ______________________________

Stephen Barr, PhD
President and Managing Director

Signature ______________________________

Date ______________________________

For CRO:

Coordinating Author:
Irene Gow
Ockham Oncology

Signature ______________________________

Date 26 SEP 14

Principal Investigator Declaration

I have read this protocol and agree that it contains all the necessary details for carrying out this study. I will conduct the study as described in this document and will make every reasonable effort to complete the study within the time designated. I verify that I am suitably qualified by education, scientific and medical training and experience to conduct the study. Documentation of my qualifications and professional affiliations are contained in my up-to-date curriculum vitae.

I will provide appropriate copies of the protocol, including future protocol amendments, and all information relating to non-clinical, and clinical experience when available (e.g. in updated editions of the Investigator's Brochure), to all staff in my unit involved in the conduct of this study. I will discuss this material with them to ensure that they are fully conversant with medical treatment and study design, and that they will handle the data and information generated in the study confidentially.

I will conduct the study in accordance with Good Clinical Practice, the Declaration of Helsinki, and the moral, ethical and scientific principles that justify medical research. The study will be conducted in accordance with the relevant laws and regulations relating to clinical studies and the protection of patients. All patients will be informed comprehensively about the nature of the study and will give their written consent to participate before entry into the study. They will be informed that they may withdraw from the study at any time. I will use only the information sheet and consent form approved by Almac and the Ethics Committee (EC) for this study. I will supply Almac with any written material prepared by myself (e.g. study summaries) which are given to the EC in support of the application.

Where applicable, the patient information contained in clinic records, reports and manuscripts will be transcribed to the case report forms (the case report form may be the original source document for specified items). Either I or an appointed person will attest to the authenticity of the data and accuracy and completeness of the transcription by signing the case report form. I agree to the audit and monitoring procedures that involve verification of such study records against original records. Should it be requested by government regulatory agencies, I will make available additional background data from my records, and where allowed, from the hospital or institution where the study was conducted.

I understand that the case report forms and other data pertinent to this study are the property of Almac and are confidential. I will supply Almac with the study data in such a way that the patient cannot be personally identified.

Investigator Signature:____________________________________ Date:________________

Print Name: ____

Address: ____

Telephone Number: ____

OTHER CONTACT INFORMATION

Full contact details for each Investigational site, the Sponsor, Medical Monitor and other key coordinating and operational personnel involved in this clinical trial (including laboratories and vendors), will be maintained and available for reference in the Trial Master File and in each Site Study File.

PROTOCOL SYNOPSIS

Protocol No:
ALM201/0001

Study Title:
A phase I open-label multicentre dose-escalation study of subcutaneous ALM201 in patients with advanced ovarian cancer and other solid tumours.

Investigational Product:
ALM201

Phase of Development:
1

No of Sites:
For dose escalation (Part 1): 3 UK sites

For further assessment of the Maximum Tolerated Dose (MTD), Maximum Feasible Dose (MFD) and/or Biologically Active Dose (BAD) in Part 2: up to 5 additional UK sites may be added in order to ensure there is an acceptable enrolment rate.

No of Patients:
The final sample size will depend on the number of Dose Limiting Toxicities (DLTs) observed at each dose level and any Cohort Review Committee (CRC) decision to assess an alternative treatment schedule in Part 1, plus the number of enrichment cohorts assessed at the MTD, MFD and/or BAD in Part 2.

Up to 36 evaluable patients would be anticipated for Part 1 based on a conservative estimate of enrolling a maximum of 36 patients across 6 dose escalation cohorts. Up to an additional 12 patients may also be enrolled to Part 1 for the assessment of alternative dose schedule(s).

No more than 36 evaluable patients will be enrolled for Part 2. This upper limit is based on an estimate of up to 12 patients being evaluated at 3 dose levels of interest.

Study Objectives and Endpoints:
The primary objectives of this study are to characterize the safety and tolerability of ALM201 and to identify a recommended phase 2 dose and schedule of administration of ALM201 in patients with advanced ovarian cancer.

Objective	Endpoint(s) (Assessment)
Primary:	
• To characterise the safety and tolerability of ALM201 (Parts 1 and 2)	• Ongoing evaluation of AEs during treatment and follow up; evaluation of DLT during Cycle 1 (Part 1 only)
• To identify a recommended phase 2 dose and schedule of ALM201 (Part 2 only)	• Safety, PK, PD and tumour response assessments
Secondary:	
• To establish the pharmacokinetic profile of ALM201	• Assessment of pharmacokinetic variables (including C_{max}, C_{min}, AUC)
• To assess anti-tumour activity	• Tumour response assessment by RECIST 1.1(Eisenhauer et al, 2009[i]) and/or other relevant response assessments for tumour types enrolled
• To assess anti-tumour activity in a biomarker-enriched group of patients with advanced ovarian cancer (Part 2 only)	• Tumour response assessment by RECIST 1.1(Eisenhauer et al, 2009[i])
Exploratory:	
• To assess relevant tumour biomarkers and the pharmacodynamic activity of ALM201	• Assessment of relevant tumour biomarkers and markers of ALM201 activity<br>o in archival and/or fresh tumour biopsy material e.g. CD44, FKBPL, CD31, pFAK, pHer2, ITGA5, CA-125, ER, PR, angiogenesis signature and other relevant or exploratory biomarkers as appropriate for tumour type<br>o in blood e.g. RASSF1 methylation, and other relevant or exploratory biomarkers appropriate for tumour type<br>o in ascites (Part 2 only) e.g. stem cell count, and other relevant or exploratory biomarkers appropriate for ovarian cancer

Study Design:

This is a Phase 1, open-label, dose escalation study of the safety, tolerability, and pharmacokinetics (PK) of ALM201. The study will commence by enrolling patients with advanced solid tumours in whom treatment with an anti-angiogenic agent is appropriate. Eligible participants will be enrolled in sequential cohorts treated with ALM201, given as a sub-cutaneous (SC) injection while being monitored for safety and DLTs.

Dose levels will not be weight-adjusted and the starting dose for the study will be 10 mg ALM201 given on Days 1-5, 8-12 and 15-19 every 21 days i.e. weekday dosing. Dose increments will not exceed 100% and will be guided by safety data observed during Cycle 1, as well as on-going assessment of safety beyond Cycle 1 in earlier cohorts, plus PK and PD data as available. Every new dose cohort will be evaluated for the occurrence of a DLT during Cycle 1 of treatment (Section 4.2.1).

Accelerated enrolment followed by a 3+3 design

Part 1 of the study will commence with an accelerated dose escalation schedule and enrol 1 patient into a cohort with follow up for adverse events (AEs) and DLT during Cycle 1. The CRC will meet as soon as possible once the final study visit in Cycle 1 has taken place in each cohort to review patient safety data to the end of Cycle 1. Where there are no safety concerns, a 100% dose escalation step will be permitted for the next cohort. Where the CRC suspects that drug-related events have occurred that could progress to DLT upon further dose escalation e.g. clinically significant NCI (National Cancer Institute) CTCAE (Common Terminology Criteria for Adverse Events) Grade 2 events considered to be related to ALM201, they will confirm that future cohorts must enrol at least 3 patients in order to more thoroughly evaluate potential drug-related adverse events. The CRC may also advise that the current dose level under evaluation be expanded to 3 patients prior to further dose escalation in order to help inform the next dose escalation step. Dose escalation of 3-patient cohorts will proceed according to the scheme presented in Section 4.2. Note that there will always be stagger of at least 1 week between dosing the first and subsequent patients in a new dose cohort. The CRC may request there be a further or prolonged stagger introduced during the trial, depending on the nature of adverse events observed to date.

Dose Limiting Toxicity

The CRC will agree on the next appropriate dose escalation step for each cohort primarily based on DLT evaluation during Cycle 1 of the current cohort. Only events occurring during the first cycle of treatment will be considered for DLT determination; however, there will be on-going evaluation of AEs in subsequent treatment cycles during the cohort review process. Clinically significant events thought to be potentially related to ALM201, or trends in adverse events seen in subsequent cycles will be taken into account when considering future dose escalation steps and dose administration schedules.

Upon occurrence of the first DLT in any cohort, additional patients will be added to that cohort so that up to a total of 6 can be evaluated. Once expansion to a 6-patient cohort has been recommended due to the identification of a DLT, escalation to the next 3-patient cohort will only occur when all patients in the expanded cohort have completed their first cycle of ALM201 and no more than 1 DLT has occurred. If 2 or more DLTs occur in an expanded cohort, DLT is established and the next lower dose level will be declared the MTD.

The highest dose where ≤ 1 DLT is seen in 3 or 6 patients will be termed the MTD. Note that intermediate dose levels may be explored below the dose level where ≥ 2 DLTs were seen, in order to identify the maximum dose which may be adequately tolerated. The CRC may also specify a recruitment stagger to be followed during cohort expansion for DLT evaluation depending on the nature of the DLT and the considered risk to patients.

In the case where an MTD is not established, the maximum feasible single dose which may be administered will be dictated by the formulation of ALM201 and the maximum volume for SC administration i.e. 3 x 1 mL injections, which will administer a dose of 300 mg ALM201 (Section 6). Should this dose be reached without the need to de-escalate due to DLT, it will be termed the MFD.

Based on the review of PD data in conjunction with on-going safety and PK data, the CRC may also identify a BAD for further exploration in Part 2 of the study.

Safety evaluations will be conducted weekly during each treatment cycle, with DLT assessed during Cycle 1 only. All events and suspected DLTs will be graded according to the CTCAE v4.03.

A DLT is defined as a Grade 3 or 4 AE that, in the opinion of the CRC, is likely to be related to ALM201 and represents a clinically significant hazard to the subject. Qualifying DLT events must be considered to be clinically relevant e.g. in duration, apparent reversibility, required management, and upon consideration of the patient's medical history and/or concomitant medications. DLT events must also be evaluated in terms of what is considered to be an appropriate next escalation step: in the case where the CRC agree that an escalation step of approximately 33% or lower is merited, the toxicity of concern should be declared a DLT.

Examples of exceptions that will be considered by the CRC are as follows:

- Grade 3 or 4 laboratory abnormalities, which resolve spontaneously or can be corrected with appropriate treatment (such as electrolytes) e.g. an event returns to baseline or to Grade 1 or less prior to the next administration);
- Symptomatic adverse events, such as nausea, vomiting and diarrhea, if they can be reduced to less than Grade 3 with standard supportive measures, such as anti-emetics and anti-diarrhoeals within 72 hours.

In order to be evaluable for DLT assessment, a patient must have received at least 80% of their scheduled doses (e.g. 12 of the 15), unless this lack of compliance is due to ALM201-related toxicity. DLT events must therefore be considered in terms of inability to administer the planned Cycle 1 dose administration schedule, and in such cases a dose delay of more than 14 days due to a toxicity event considered related to ALM201 will be considered to be a DLT (Section 4.4.1).

Part 1: Dose Escalation

In the absence of DLT or suggestion of ALM201-related adverse events which would lead to more cautious dose escalation, the following table presents a hypothesised dose escalation plan:

Cohort	Escalation Step	Dose level
**1 (Starting Dose)**	-	10 mg
**2**	(2X starting dose)	20 mg
**3**	(4X starting dose)	40 mg
**4**	(8X starting dose)	80 mg
**5**	(16X starting dose)	160 mg
**6 (MFD)**	(30X starting dose)	300 mg

The dose may be doubled in sequential cohorts where the CRC consider it appropriate to do so, based on on-going evaluation of DLTs and adverse event data. Dose escalation decisions will also take available PK and pharmacodynamic (PD) data into consideration.

In the case where a potentially significant toxicity occurs, or a trend in toxicities is seen, considered to be related to treatment with ALM201 and potentially a precursor to a clinically significant toxicity event, subsequent dose escalation steps will be more conservative and will not exceed 50% of the previous dose. This restriction may be reversed where there is no suggestion of potentially clinical significant toxicity in subsequent cohort(s). In the case where a single DLT event is confirmed in an expanded cohort, the next dose escalation step will not exceed 33% of the previous dose. However, where there are no further events seen in the next cohort; the CRC may allow future dose escalation steps of up to 50%.

Note that the CRC may also recommend that an MTD, MFD or BAD be assessed in a cohort of 6 patients in Part 1 where only 3 patients have received this dose level to date, prior to recommending the enrolment of an enrichment cohort to receive this dose level in Part 2.

Cohort Review Committee

All dose escalation decisions will be made by a CRC who shall convene to review all available AE, PK, PD and relevant patient data (Section 8.1). Patients in Part 1 will be eligible for DLT evaluation if they have received 80% e.g. 12 of their 15 scheduled doses, during Cycle 1. The CRC will be composed of the trial investigators, a patient representative, sponsor representative(s) and the study Medical Monitor. Additional experts may be invited to support the review of the data as required e.g. a pharmacokineticist. The Contract Research Organisation (CRO) responsible for managing and monitoring the trial will also support the preparation and conduct of the CRC meetings and may attend such meetings. All data reviewed, CRC discussions and agreed dose escalation recommendations will be minuted. The composition of the CRC and the data review processes to be followed by the committee will be fully described in a CRC Charter.

Based on on-going safety, PK and PD data evaluation, the CRC may also recommend dose de-escalation steps or adjustments in the dose administration schedule of ALM201. Upon review of available safety, PK and PD data, the CRC may recommend that an alternative dose administration schedule of ALM201 be explored. Less intense dose administration schedules will be permitted as long as the unit dose does not exceed the next permitted escalation step. Given the starting dose administration schedule is week-day dosing i.e. D1-5, D8-12, D15-19, on a 21 day cycle, a more dose intense dosing schedule is unlikely. However, should the CRC consider this reasonable to explore, the same dose escalation rules will apply and pharmacokinetic modelling, in conjunction with the safety data obtained to date, will be used to inform the appropriate dose level for a more dose intense dose schedule.

Note that the MTD or MFD need not be confirmed for the original dose administration schedule prior to the CRC recommending the investigation of an alternative dose administration schedule. Multiple dose escalation tracks may be followed if more than one dose schedule is considered relevant to explore i.e. the CRC may recommend that the alternative dose administration schedule

replaces a schedule or is explored in addition to another schedule, as long as dose escalation rules are not exceeded.

Duration of treatment

The main study will permit a maximum of 8 three-weekly treatment cycles (or approximately 6 months of treatment). Patients who are seen to potentially be benefiting from treatment i.e. patients whose disease has not progressed and who have not been withdrawn from therapy due to toxicity, will be eligible to continue to receive additional cycles of ALM201 where this is recommended by their study physician, subject to availability of ALM201. Such patients will continue to be followed up for compliance, toxicity and continued response (Schedule of Study Assessments).

Part 2: Enrichment at MTD, MFD and/or BAD

Once an MTD, MFD and/or BAD has been established for a given dose and schedule in Part 1, Part 2 may commence. Part 2 will involve up to an additional 36 patients with advanced ovarian cancer. During Part 2 of the study there will be on-going evaluation of safety and the opportunity to obtain preliminary anti-tumour activity at each dose level selected in the ovarian cancer population. The only dose adjustments permitted in Part 2 will be dose de-escalations (based on a de-escalation of the unit dose or a less frequent administration schedule of the maximum unit dose). Such decisions will be driven by each patient's tolerability of ALM201. Where possible, such dose reductions should be made by the CRC who can convene on an ad-hoc basis to advise on the appropriate dose adjustment; however, the investigator may implement a dose reduction prior to CRC review in the interest of patient safety.

Study Assessments:

The study will commence with a dosing schedule of ALM201 monotherapy given SC on Days 1-5, 8-12 and 15-19 in a 3 week cycle.

Patients will have scheduled site visits on every dosing day of the first cycle, then on Days 1, 8 and 15 of Cycles 2-4. From Cycle 5 onwards, they are only required to visit the clinic on Day 1 of each cycle. ALM201 administration can be given at home on all other days.

Safety assessments will include physical examination, vital signs, biochemistry and haematology laboratory screens, plus immunogenicity testing (see Schedule of Study Assessments). Adverse events will also be noted at every clinical visit and recorded at least every week. For all administrations in hospital, the patients must wait for at least 60 minutes from the time of the ALM201 injection for observation and repeat vital signs. These precautions are in case of emergent evidence of immunogenicity in Cycle 1, and at other times after the weekend break in dosing.

Tumour assessment by imaging (computed tomography (CT) scan or magnetic resonance imaging (MRI) scan as appropriate for tumour type) will be assessed in all patients at Screening and after every 2 cycles of treatment (i.e. every 6 weeks) during Cycles 1–8 (first 24 weeks), and then after every 4 cycles of treatment (i.e. every 12 weeks) from Cycle 9 onwards. Scans may be performed at other times as clinically indicated.

Tumour assessment by informative tumour markers where relevant for tumour type e.g. GCIG

criteria for CA125 (Rustin, et al. 2011[ii]), PSA, CEA or CA19-9, will be assessed in all patients at Screening and after every 2 cycles of treatment during treatment (i.e. every 6 weeks). Tumour markers may be performed at other times as clinically indicated.

A PK profile for ALM201 will be taken on Days 1, 3 and 18 of Cycle 1 and on Day 18 of Cycles 2, 4, 6 and 8. Pre-dose samples will also be taken on Cycles 2-8 on Day 1.

All patients will be asked to provide consent for access to archived tumour tissue where available, and where possible, fresh biopsies will be taken pre-dose and again during the study where the patient has a documented tumour response and/or at the point of disease progression to allow for potential biomarker and pharmacodynamic assessment. In Part 2, access to a tumour biopsy sample will be required to confirm each patient's eligibility for the study (see Inclusion Criterion 2). A fresh biopsy will be preferable for this purpose; however, where it is not possible to obtain this, confirmation of the angiogenesis signature may be performed on archived tumour tissue during Screening. In Part 2, there will also be additional biomarker/PD assessments in blood and ascites. In both parts of the study, any remaining samples obtained for biomarker/PD assessment will be retained for potential future analysis.

Patients will be withdrawn from the study at the point of receiving their last dose of ALM201. However, all patients will be asked to participate in a protocolled follow up assessment 4 weeks after their last dose for safety assessments. Furthermore, patients will continue to be followed up either in the study clinic or by telephone contact every 8 weeks (Schedule of Study Assessments) for up to approximately a 2 year period, to check their disease (survival) status and commencement of next anti-cancer treatment.

Inclusion/Exclusion Criteria:

Part 1 will enrol patients with advanced solid tumours in whom treatment with an anti-angiogenic agent is appropriate. Part 2 will enrol patients with ovarian cancer, screened using an angiogenesis gene signature, that have failed to respond to, or have relapsed following standard therapy.

Inclusion Criteria

(i) Part 1 Specific Inclusion Criterion

1. Patients with histologically and/or cytologically confirmed advanced solid tumour for whom no standard effective therapy is available or felt likely to be of limited efficacy and in whom a rationale for use of an anti-angiogenic treatment approach exists. Note: *Previous use of anti-angiogenic therapy is allowed if tolerated*

(ii) Part 2 Specific Inclusion Criterion

2. Patients with advanced ovarian cancer, who are intolerant of or whose tumour is resistant to platinums and who have failed to respond to, or have relapsed following, standard therapy and whose tumour has a proangiogenic profile as assessed by the angiogenesis gene signature test. Note: *Previous use of anti-angiogenic therapy is allowed if tolerated.*

(iii) General Inclusion Criteria for all Patients

3. Adult patients defined by age ≥16 years at time of consent.

4. Evaluable disease, either measurable on imaging, or with informative tumour marker(s), as assessed by RECIST 1.1 (Eisenhauer et al, 2009[i]) or other relevant response assessment criteria for tumour type.

5. Recovery from previous treatment to baseline or CTCAE ≤ Grade 1, as determined by CTCAE v4.03 criteria (Appendix B), of reversible toxicities related to prior treatment, with the exception of alopecia, lymphopenia, other non-clinically significant adverse events; recovery from previous radiotherapy other than residual cutaneous effects or stable < Grade 2 gastrointestinal toxicity; complete recovery from surgery other than stable < Grade 2 toxicity.

6. Eastern Collaborative Oncology Group (ECOG) Performance Status (PS) of 0 or 1 (Appendix A).

7. Laboratory values at Screening:
   - Absolute neutrophil count ≥1.5 x 10^9/L without colony stimulating factor support;
   - Platelets ≥100 x 10^9/L;
   - Haemoglobin ≥9 g/dL (not transfusion dependent);
   - Total bilirubin <1.5 times the upper limit of normal (ULN) (unless due to Gilbert's syndrome where it should be ≤ 2.5 x ULN);
   - AST (SGOT) ≤2.5 times the ULN; ALT (SGPT) ≤2.5 times the ULN; ≤5 x ULN for patients with advanced solid tumours with liver metastases; patients with confirmed bony metastases will be permitted on study with isolated elevations in ALP < 5 times the ULN;
   - Serum creatinine ≤ 1.5 x ULN or estimated glomerular filtration rate (GFR) of >50 mL/min based on the Cockcroft-Gault formula (Appendix D);
   - Normal coagulation (elevated INR, prothrombin time or APTT ≤ 1.3 x ULN range acceptable);
   - Urine protein ≤2+ (as measured by dipstick).

8. Negative urine or blood human chorionic gonadotropin (hCG) test during Screening and within 7 days of Cycle 1, Day 1 in women of childbearing potential (defined as women ≤ 50 years of age or history of amenorrhea for ≤ 12 months prior to study entry). Sexually active male and female patients of childbearing potential must agree to use an effective method of birth control e.g. barrier methods with spermicides, oral or parenteral contraceptives and/or intrauterine devices, during the entire duration of the study and for 6 months after final administration of ALM201. Note that sterility in female patients must be confirmed in the patients' medical records and can be defined as any of the following: surgical hysterectomy with bilateral oophorectomy, natural menopause with menses >1 year ago; radiation induced oophorectomy with last menses >1 year ago; chemotherapy induced menopause with 1 year interval since last menses.

9. Ability to give written, informed consent prior to any study-specific Screening procedures, with the understanding that the consent may be withdrawn by the patient at any time without prejudice.

10. Patient is capable of understanding the protocol requirements, is willing and able to comply with the study protocol procedures, and has signed the informed consent document.

Exclusion Criteria for all Patients

1. History of inability to tolerate anti-angiogenic therapies e.g. increased blood pressure (BP), proteinuria, prior thromboembolic events.

2. Previous history of bowel obstruction, clinical evidence of gastro-intestinal obstruction, large burden of peritoneal disease or evidence of bowel involvement on computed tomography.

3. Patents has received:

   a) any chemotherapy regimens (including investigational agents) with delayed toxicity within 4 weeks (6 weeks for prior nitrosourea or mitomycin C) of Cycle 1, Day 1, or received chemotherapy regimens given continuously or on a weekly basis which have limited potential for delayed toxicity within 2 weeks of Cycle 1, Day 1.

   b) radiotherapy, immunotherapy or biological agents (includes investigational agents) within 4 weeks of Cycle 1, Day 1. Localised palliative radiotherapy is permitted for symptom control.

4. Documented, symptomatic or uncontrolled intracranial metastases or primary intracerebral tumours.

5. Cancer with leptomeningeal involvement.

6. On therapeutic anti-coagulation (aspirin dosing ≤100 mg per oral (PO) daily allowed).

7. Previous malignancy, except for non-basal-cell carcinoma of skin or carcinoma-in-situ of the uterine cervix, unless the tumour was treated with curative intent more than 2 years prior to study entry.

8. History of clinically significant cardiac condition, including uncontrolled hypertension (BP >140/90 mmHg, despite medical therapy); left ventricular systolic dysfunction (ejection fraction (<55 %) on echocardiography) with or without heart failure symptoms; history of an ischaemic cardiac event within 3 months of study entry (myocardial infarction, acute coronary syndrome); QT interval prolongation (QTcF, Fridericia's Correction of >450 ms on screening 12-lead ECG); clinically significant cardiac arrhythmia within 3 months of study entry. Note: *ventricular tachycardia, ventricular fibrillation, supraventricular tachycardia, atrial fibrillation without adequate heart rate control, atrial fibrillation with adequate heart rate control with or without medication or other treatment, are not an exclusion.*

9. Known human immunodeficiency virus positivity.

10. Active hepatitis B or C or other active liver disease (other than malignancy).

11. Any active, clinically significant, viral, bacterial, or systemic fungal infection within 4 weeks prior to Cycle 1, Day 1.

12. Any evidence of severe or uncontrolled systemic conditions or any other issues which make it undesirable for the patient to participate in the study or which could jeopardize compliance with the protocol.

Route of Administration, Dose Schedule and Duration:

ALM201 administered as a SC injection with a maximum administration volume of 1.0 mL per injection. Each 1 ml injection will contain 100 mg ALM201. A maximum of 3 x 1.0 mL injections may be given for any single dose, therefore the MFD will be 300 mg. The starting dose level of ALM201 is 10 mg given on Days 1-5, 8-12 and 15-19 of a 21-day cycle. The SC injection may be given in the abdomen, leg or arm following local administration guidelines including the use of premedication as required for local injection site reactions. The dose levels assessed in this study i.e. the dose and dosing administration schedule, may be adjusted following the study dose escalation rules, based on review of on-going evaluation of safety, PK and PD data generated during the study.

Statistical Methods:

There will be no formal statistical analysis in the dose-escalation phase this study. Results will be listed and summarised using descriptive statistics. In Part 2, response rate will be assessed and summarised and an exact 95% confidence interval (CI) calculated. Analyses will be performed on the whole population plus those who have and have not been previously exposed to anti-angiogenics and those who have and not been exposed to tubulin-targeting agents before entry to this trial.

SCHEDULE OF STUDY ASSESSMENTS

(i) Schedule of Assessments for Starting Dose Administration Schedule: Day 1-5, 8-12 and 15-19 of a 21-day dosing schedule

	Screening	Cycle 1 (21 d cycle)							Cycle 2 – 4 (21 d cycle)						Cycle 5 – 8 (21 d cycle)							Final Study Visit[11]	Long-term Follow Up
		Day							Day						Day								
	-28 to 0	1	2	3-5	8	9-12	15	16-19	22/1[10]	2-5	8	9-12	15	16-19	22/1[10]	2-5	8	9-12	15	16-19	22/1[10]		
Informed consent	X																						
Demographics	X																						
Medical history	X																						
Inclusion/exclusion	X																						
ECOG PS	X								X						X						X	X	
Physical examination[1]	X	X			X		X		X		X		X		X		X		X		X	X	
Vital signs[2]	X	X	X	X	X	X	X	X	X		X		X		X		X		X		X	X	
ECG (resting 12-lead)[3]	X	X							X						X						X		
Clinical chemistry*	X	X			X		X		X		X		X		X		X		X		X	X	
Echocardiogram	X																						
Haematology	X	X			X		X		X		X		X		X		X		X		X	X	
Coagulation	X	X			X		X		X		X		X		X		X		X		X		
Urinalysis*	X	X							X						X						X		
Tumour assessment (radiological) [4]	X								X[4]						X[4]						X[4]	X[4]	
Tumour assessment (serum marker)[4]	X	X							X						X						X[4]	X[4]	
Immunogenicity assessment		X							X						X						X	X	
Biomarker/PD assessment (biopsy)[5]	X	X - up to 2 post-treatment samples																					
Biomarker/PD assessment (blood sample)[6]	X	X - up to 12 post-treatment samples																					
Biomarker/PD assessment (ascites)[7]	(X)	(X - up to 5 post-treatment samples)																					
Biomarker (germ-line DNA) testing[8]	(X)																						
Adverse events		X	X	X	X	X	X	X	X		X		X		X		X		X		X	X	
Concomitant medication	X	X	X	X	X	X	X	X	X		X		X		X		X		X		X	X	
ALM201 administration		X	X	X	X	X	X	X	X	X	X	X	X	X	X	X	X	X	X	X	X		
Pharmacokinetics[9]		X	X	X				X	X					X	X					X	(X)		
Long-term follow up[12]																							X

Footnotes - General

- Assessments made on Day 1 of each cycle are to be conducted prior to ALM201 administration, unless specified otherwise.
- Additional assessments may be conducted as clinically indicated.
- (X) denotes an assessment not applicable to all patients.
- A tolerance of +/-1 day will be permitted for all study visits and a tolerance of -1 day for all assessments relative to the study visit, unless specified otherwise.

* ***female patients, if fertile, will require a serum pregnancy test at Screening and urine pregnancy on Day 1 of each cycle***

Assessment Specific

1. Patient's height will be recorded at Screening. A full physical examination is required at Screening and prior to Day 1 of each cycle; Symptom-directed physical examination is acceptable at other time-points. Weight will be recorded at Screening and on Day 1 of each cycle.

2. On each hospital administration day, vital signs (heart rate, BP, temperature and respiration rate) will be assessed pre-dose and up to 1 hour after the ALM201 injection. Patient status will be monitored during ALM201 administration and repeat vital signs will be taken if needed.

3. On Cycle 1, Day 1 a resting 12-lead ECG will be conducted pre-dose and 30 mins (+/- 15 mins) after ALM201 injection. On Day 1 of all other cycles, a resting 12-lead ECG will be conducted pre-dose only.

4. CT or MRI performed at Screening and up to 7 days prior to start of Cycle 3, 5, 7 and at the end of Cycle 8. Note that where there is a rationale for assessment of bone lesions, these assessments will be performed as part of the CT or MRI assessment and will not require additional radiological bone scan assessment.

   Other informative markers e.g. CA-125, PSA, photographs of melanoma skin lesions, may be taken as appropriate on Day 1 of each cycle.

   Additional scans may be performed to confirm a Complete Response (CR) or Partial Response (PR) or disease progression (PD) as per appropriate response assessment guidelines. Any requirement for confirmatory scans will typically be performed at the next protocolled assessment time point. Other assessments e.g. whole body MRI or PET, are not protocol mandated, but may be performed as clinically indicated and at request of Investigator.

5. Patients with available archived biopsy samples will consent to provide these for biomarker/PD evaluation. The study will encourage taking fresh biopsies for biomarker/PD evaluation at Screening and post-treatment upon tumour response and/or at the point of disease progression. Although optional, every effort should be made to collect fresh pre and post-dose biopsy samples from patients – particularly in Part 2 - and imaging techniques may be used to facilitate this process.

6. Assessment of biomarker/PD activity from blood samples taken to obtain serum, plasma, PBMC or CTCs, may be conducted in all patients between Screening, Cycle 6 and Final Study Visit, with no more than 2 samples taken on any study day, 4 in any treatment cycle, and 13 in total during 6 cycles (including Screening and Final Study Visit). Time-points may vary depending on the assay and method of analysis.

7. Assessment of biomarker/PD activity in ascites may be conducted in relevant patients who are undergoing draining of ascites as part of their standard of care. This procedure would normally be performed under ultrasound marking. It is estimated that the study may obtain up to 6 samples over 8 cycles (including Screening and Final Study Visit). Actual time-points may vary within each cycle.

8. Consenting patients will have a 10 mL blood sample taken for preparation of a germ-line DNA sample at Screening (recommended time-point only).

9. Patients will have a 12-hour urine collection on Cycle 1, Day 1 for urine PK analysis.

   Patients will have PK blood sampling conducted at Cycle 1 at the following sample times. Three PK profiles may be taken: each will not exceed up to 12 samples taken out to 24 hours post ALM201 injection. The CRC may advise on adjusted time-points. The maximum number of PK samples to be collected during any Cycle 1 dose schedule will not exceed 40. The actual time for each blood draw must be accurately recorded. Initial sampling time points are:

   Cycle 1, Day 1:
   All doses (except 300mg): Predose, then 15 mins (+/- 5 mins), 45 mins (+/- 5 mins), 1.5 hr (+/- 10 mins), 2 hr (+/- 10 mins), 3 hr (+/- 10 mins), 4 hr (+/- 10 mins), 5 hr (+/- 10 mins) , 6 hr (+/- 10 mins), 22 hr (+/- 1hr).

   Doses of 300 mg: Predose, then 15 mins (+/- 5 mins), 45 mins (+/- 5 mins), 1.5 hr (+/- 10 mins), 2 hr (+/- 10 mins), 3 hr (+/- 10 mins), 4 hr (+/- 10 mins), 5 hr (+/- 10 mins) , 6 hr (+/- 10 mins), 7 hr (+/- 10 mins), 8 hr (+/- 10 mins), 22 hr (+/- 1hr).

   Cycle 1, Day 3 & 18:
   All doses (except 300mg): Predose, then 30mins (+/- 5 mins), 60 mins (+/- 5 mins), 1.5 hr (+/- 10 mins), 2 hr (+/- 10 mins), 3.5 hr (+/- 10 mins), 5 hr (+/- 10 mins).

   Doses of 300 mg: Predose, then 30 mins (+/- 5 mins), 60 mins (+/- 5 mins), 1.5 hr (+/- 10 mins), 2 hr (+/- 10 mins), 4.5 hr (+/- 10 mins), 7 hr (+/- 1 hr).

   Cycles 2, 4, 6 & 8, Day 18:
   All doses (except 300mg): Predose, then 30mins (+/- 5 mins), 60 mins (+/- 5 mins), 1.5 hr (+/- 10 mins), 2 hr (+/- 10 mins), 3.5 hr (+/- 10 mins), 5 hr (+/- 10 mins).

   Doses of 300 mg: Predose, then 30 mins (+/- 5 mins), 60 mins (+/- 5 mins), 1.5 hr (+/- 10 mins), 2 hr (+/- 10 mins), 4.5 hr (+/- 10 mins), 7 hr (+/- 1 hr).

   A single pre-dose sample will also be taken on Cycles 2-8 on Day 1.

10. Day 22 of Cycle 1, 2, 3, 4, 5, 6 and 7 is Day 1 of Cycle 2, 3, 4, 5, 6, 7 and 8.

11. The Final Study Visit should be performed 30 +/-3 days after the last dose of ALM201 to enable a final safety assessment.

12. Those patients who do not have disease progression at the Final Study Visit will be contacted every 8 weeks for up to 2 years (approximately) to check their status and commencement of their next anticancer treatment.

(ii) Schedule of Study Assessments for Alternative Dose Schedules

	Screening	Cycle 1				Cycle 2 – 4			Cycle 5 – 8				Final Study Visit[11]	Long-term Follow Up
		Day				Day			Day					
	-28 to 0	1	2	Other dosing days	Weekly clinic visits	22/1[10]	Other dosing days	Weekly contact	22/1[10]	Other dosing days	Weekly contact	22/1[10]		
Informed consent	X													
Demographics	X													
Medical history	X													
Inclusion/exclusion	X													
ECOG PS	X					X			X			X	X	
Physical examination[1]	X	X			X	X		X	X		X	X	X	
Vital signs[2]	X	X			X	X		X	X		X	X	X	
ECG (resting 12-lead)[3]	X	X				X			X			X		
Echocardiogram	X													
Clinical chemistry*	X	X			X	X		X	X		X	X	X	
Haematology	X	X			X	X		X	X		X	X	X	
Coagulation	X	X			X	X		X	X		X	X		
Urinalysis*	X	X				X			X			X		
Tumour assessment (radiological)[4]	X					X[4]			X[4]			X[4]	X[4]	
Tumour assessment (serum marker)[4]	X	X				X			X			X[4]	X[4]	
Immunogenicity assessment		X				X			X			X		
Biomarker/PD assessment (biopsy)[5]	X	X – up to 2 post-treatment samples												
Biomarker/PD assessment (blood sample)[6]	X	X - up to 12 post-treatment samples												
Biomarker/PD assessment (ascites)[7]	(X)	(X – up to 5 post-treatment samples)												
Biomarker (germ-line DNA) testing[8]	(X)													
Adverse events		X	X	X	X	X		X	X		X	X	X	
Concomitant medication	X	X	X	X	X	X		X	X		X	X	X	
ALM201 administration		X (D1 + additional doses following dosing Schedule)				X (as per dosing schedule)								
Pharmacokinetics[9]		X	X	X		X	X		X	X		(X)		
Long-term follow up[12]														X

Footnotes - General

- Assessments made on Day 1 of each cycle are to be conducted prior to ALM201 administration, unless specified otherwise.
- Additional assessments may be conducted as clinically indicated.
- (X) denotes an assessment not applicable to all patients.
- A tolerance of +/-1 day will be permitted for all study visits and a tolerance of -1 day for all assessments relative to the study visit, unless specified otherwise.

* ***female patients, if fertile, will require a serum pregnancy test at Screening and urine pregnancy on Day 1 of each cycle***

Assessment Specific

1. Patient's height will be recorded at Screening. A full physical examination is required at Screening and prior to Day 1 of each cycle; Symptom-directed physical examination is acceptable at other time-points. Weight will be recorded at Screening and on Day 1 of each cycle.

2. On each hospital administration day, vital signs (heart rate, BP, temperature and respiration rate) will be assessed pre-dose and up to 1 hour after the ALM201 injection. Patient status will be monitored during ALM201 administration and repeat vital signs will be taken if needed.

3. On Cycle 1, Day 1 a resting 12-lead ECG will be conducted pre-dose and 30 mins (+/- 15 mins) after ALM201 injection. On Day 1 of all other cycles, a resting 12-lead ECG will be conducted pre-dose only.

4. CT or MRI performed at Screening and up to 7 days prior to start of Cycle 3, 5, 7 and at the end of Cycle 8. Note that where there is a rationale for assessment of bone lesions, these assessments will be performed as part of the CT or MRI assessment and will not require additional radiological bone scan assessment.

   Other informative markers e.g. CA-125, PSA, photographs of melanoma skin lesions, may be taken as appropriate on Day 1 of each cycle.

   Additional scans may be performed to confirm a Complete Response (CR) or Partial Response (PR) or disease progression (PD) as per appropriate response assessment guidelines. Any requirement for confirmatory scans will typically be performed at the next protocolled assessment time point. Other assessments e.g. whole body MRI or PET, are not protocol mandated, but may be performed as clinically indicated and at request of Investigator.

5. Patients with available archived biopsy samples will consent to provide these for biomarker/PD evaluation. The study will encourage taking fresh biopsies for biomarker/PD evaluation at Screening and post-treatment upon tumour response and/or at the point of disease progression. Although optional, every effort should be made to collect fresh pre and post-dose biopsy samples from patients – particularly in Part 2 - and imaging techniques may be used to facilitate this process.

6. Assessment of biomarker/PD activity from blood samples taken to obtain serum, plasma, PBMC or CTCs, may be conducted in all patients between Screening, Cycle 6 and Final Study Visit, with no more than 2 samples taken on any study day, 4 in any treatment cycle, and 13 in total during 6 cycles (including Screening and Final Study Visit). Time-points may vary depending on the assay and method of analysis.

7. Assessment of biomarker/PD activity in ascites may be conducted in relevant patients who are undergoing draining of ascites as part of their standard of care. This procedure would normally be performed under ultrasound marking. It is estimated that the study may obtain up to 6 samples over 8 cycles (including Screening and Final Study Visit). Actual time-points may vary within each cycle.

8. Consenting patients will have a 10 mL blood sample taken for preparation of a germ-line DNA sample at Screening (recommended time-point only).

9. Patients will have a 12-hour urine collection on Cycle 1, Day 1 for urine PK analysis.

Patients will have PK sampling conducted at Cycle 1 at the following sample times. Three PK profiles may be taken: each will not exceed up to 12 samples taken out to 24 hours post ALM201 injection. The CRC may advise on adjusted time-points. The maximum number of PK samples to be collected during any Cycle 1 dose schedule will not exceed 40. The actual time for each blood draw must be accurately recorded. Initial sampling time points are:

Cycle 1, Day 1:
All doses (except 300mg): Predose, then 15 mins (+/- 5 mins), 45 mins (+/- 5 mins), 1.5 hr (+/- 10 mins), 2 hr (+/- 10 mins), 3 hr (+/- 10 mins), 4 hr (+/- 10 mins) , 5 hr (+/- 10 mins) , 6 hr (+/- 10 mins), 22 hr (+/- 1hr).

Doses of 300 mg: Predose, then 15 mins (+/- 5 mins), 45 mins (+/- 5 mins), 1.5 hr (+/- 10 mins), 2 hr (+/- 10 mins), 3 hr (+/- 10 mins), 4 hr (+/- 10 mins), 5 hr (+/- 10 mins), 6 hr (+/- 10 mins), 7 hr (+/- 10 mins), 8 hr (+/- 10 mins), 22 hr (+/- 1hr).

Cycle 1, Day 3 & 18:
All doses (except 300mg): Predose, then 30mins (+/- 5 mins), 60 mins (+/- 5 mins), 1.5 hr (+/- 10 mins), 2 hr (+/- 10 mins), 3.5 hr (+/- 10 mins), 5 hr (+/- 10 mins).

Doses of 300 mg: Predose, then 30 mins (+/- 5 mins), 60 mins (+/- 5 mins), 1.5 hr (+/- 10 mins), 2 hr (+/- 10 mins), 4.5 hr (+/- 10 mins), 7 hr (+/- 1 hr).

Cycles 2, 4, 6 & 8, Day 18:
All doses (except 300mg): Predose, then 30mins (+/- 5 mins), 60 mins (+/- 5 mins), 1.5 hr (+/- 10 mins), 2 hr (+/- 10 mins), 3.5 hr (+/- 10 mins), 5 hr (+/- 10 mins).

Doses of 300 mg: Predose, then 30 mins (+/- 5 mins), 60 mins (+/- 5 mins), 1.5 hr (+/- 10 mins), 2 hr (+/- 10 mins), 4.5 hr (+/- 10 mins), 7 hr (+/- 1 hr).

A single pre-dose sample will also be taken on Cycles 2-8 on Day 1.

10. Day 22 of Cycle 1, 2, 3, 4, 5, 6 and 7 is Day 1 of Cycle 2, 3, 4, 5, 6, 7 and 8.

11. The Final Study Visit should be performed 30 +/-3 days after the last dose of ALM201 to enable a final safety assessment.

12. Those patients who do not have disease progression at the Final Study Visit will be contacted every 8 weeks for up to 2 years (approximately) to check their status and commencement of their next anticancer treatment.

(iii) Schedule of Assessments for Cycle 9 onwards

	Cycle 9 onwards			Final Study Visit[6]	Long-term Follow Up
	Day 22/1[5]	Dosing days	Weekly contact		
ECOG PS	X			X	
Brief physical examination[1]	X			X	
Vital signs[2]	X			X	
ECG (resting 12-lead)	X				
Clinical chemistry*	X			X	
Haematology	X			X	
Coagulation	X				
Urinalysis*	X				
Tumour assessment (radiological)[3]	X[3]			X[3]	
Tumour assessment (serum marker)[3]	X			X	
Immunogenicity	X			X	
Biomarker/PD assessment (biopsy)[4]	X				
Adverse events	X		X	X	
Concomitant medication	X		X	X	
ALM201 administration		X			
Long-term follow up[7]					X

Footnotes - General

- Assessments made on Day 1 of each cycle are to be conducted prior to ALM201 administration, unless specified otherwise.
- Additional assessments may be conducted as clinically indicated.
- (X) denotes an assessment not applicable to all patients.
- A tolerance of +/-1 day will be permitted for all study visits and a tolerance of -1 day for all assessments relative to the study visit, unless specified otherwise.

* ***female patients, if fertile, will require a serum pregnancy test at Screening and urine pregnancy on Day 1 of each cycle***

Assessment Specific

1. Symptom-directed physical examination. Weight will be recorded on Day 1 of each cycle.

2. On each hospital administration day, vital signs (heart rate, BP, temperature and respiration rate) will be assessed pre-dose and up to 1 hour after the ALM201 injection. Patient status will be monitored during ALM201 administration and repeat vital signs will be taken if needed.

3. CT or MRI performed at Screening and up to 7 days prior to start of every 4 cycles. Note that where there is a rationale for assessment of bone, these assessments will be performed as part of the CT assessment and not require additional radiological bone scan assessment.

   Other informative markers e.g. CA-125, PSA, photographs of melanoma skin lesions, may be taken as appropriate on Day 1 of each cycle.

   Additional scans may be performed to confirm a Complete Response (CR) or Partial Response (PR) or disease progression (PD) as per appropriate response assessment guidelines. Any requirement for confirmatory scans will typically be performed at the next protocolled assessment time point. Other assessments e.g. whole body MRI or PET, are not protocol mandated, but may be performed as clinically indicated and at request of Investigator.

4. A post-treatment biopsy should be taken where possible if the patient has disease progression.

5. Day 22 of Cycle 9, 10, 11, 12, etc is Day 1 of Cycle 10, 11, 12, 13, etc.

6. The Final Study Visit should be performed 30 +/-3 days after the last dose of ALM201 to enable a final safety assessment.

7. Those patients who do not have disease progression at the Final Study Visit will be contacted every 8 weeks for up to 2 years (approximately) to check their status and commencement of their next anticancer treatment.

Contents

LIST OF ABBREVIATIONS 28

1 INTRODUCTION 31

1.1 Rationale for use 31

1.2 Justification for treatment in ovarian cancer 31

1.3 Summary of Non-Clinical Development 36

1.4 Summary of Clinical Exposure 39

1.5 Rationale for Dose Selection 40

1.6 Safety Guidance for Investigators 40

2 STUDY OBJECTIVES 41

3 SELECTION CRITERIA 42

3.1 Inclusion Criteria 42

3.2 Exclusion Criteria 43

3.3 Patient Withdrawal or Discontinuation 44

3.4 Replacement of Non-evaluable Patients 45

3.5 Procedures for Discontinuation 45

3.6 Study or Site Termination 45

4. INVESTIGATIONAL PLAN 47

4.1 Study Design 47

4.2 DLT Evaluation and Dose Escalation Scheme 48

4.2.1 Dose Limiting Toxicities 51

4.3 Expansion at MTD, MFD or BAD 52

4.4 Dose Adjustments 52

4.4.1 Dose Delay 52

4.4.2 Dose Reduction 53

4.4.3 Intra-Patient Dose Escalation 53

4.5 Duration of treatment and treatment beyond Cycle 8 53

5. STUDY SCHEDULE 54

5.1 Schedule of Study Assessments (i) 54

5.2 Schedule of Study Assessments (ii) 61

5.5 Volume of Blood Sampling 68

5.6 Description of Study Assessments 69

5.6.1 Adverse Events 69

5.6.2 Laboratory safety measurements 69

5.6.3 Resting 12-lead electrocardiogram (ECG) 70

5.6.4 Echocardiogram 70

5.6.5 Vital signs 70

5.6.6 Pharmacokinetic assessments (blood and urine) 70

5.6.7 Tumour assessment 73

5.6.8 Immunogenicity Assessment 74

5.6.9 Pharmacodynamic assessments 74

5.6.9.1. Archived biopsy 74

5.6.9.2. Fresh biopsy 74

5.6.9.3. Blood 75

5.6.9.4. Ascites 75

5.6.10 Germ-line DNA 75

6. STUDY MEDICATION AND ADMINISTRATION 76

6.1 Study Medication 76

6.2 Selection of Doses in the Study, Injection Duration and Duration of Treatment 76

6.3 Packaging and Labelling 76

6.4 Preparation of ALM201 for Administration 77

6.5 ALM201 Administration 77

6.6 Storage in Pharmacy 77

6.7 Replacement of Medication Vials 77

6.8 Accountability 77

6.9 Treatment Allocation 78

6.10 Blinding and Procedures for Un-Blinding the Study 78

6.11 Permitted and Restricted Concomitant Medications/Treatments 78

6.12 Pre-medication guidelines 79

6.13 Supportive care guidelines 79

7. ADVERSE EVENTS AND REPORTING REQUIREMENTS 80

7.1 Assessment of Safety 80

7.2 Adverse Event Definition 80

7.3 Importance of Adverse Event Reporting 80

7.4 Evaluating Adverse Events 81

7.4.1 Severity 81

7.4.2 Seriousness 81

7.4.3 Pregnancy 82

7.4.4 Misuse and Overdose 83

7.4.5 Investigational Product Complaints 83

7.4.6 Relationship 83

7.5 Evaluating Dose Limiting Toxicities (DLTs)/Serious Adverse Events (SAEs) 84

7.5.1 Unexpected adverse events 84

7.6 Reporting DLTs/SAEs 84

7.6.1 Reporting SAEs to the IRB or Ethics Committee and Regulatory Authorities 85

7.6.2 Follow-up information on an SAE 85

8 DATA EVALUATION: CRITERIA FOR EVALUATION OF OBJECTIVES 86

8.1 Cohort Review Committee (CRC) 86

8.2 End of Study and Study Completion 86

8.3 Statistical Considerations 87

8.4 Demographic and Other Baseline Characteristics 87

8.5 Statistical Methods for Safety Parameters 87

8.6 Statistical Methods for Pharmacokinetic Parameters 87

8.7 Evaluation of Tumour Response 88

8.8 Estimated Sample Size 88

9 QUALITY ASSURANCE 90

9.1 Data Recording 90

9.2 Study Monitoring 90

9.3 Clinical Study Audit 90

9.4 Clinical Study Report 90

9.5 Data Retention and Availability 90

10 ETHICS REVIEW/INFORMED CONSENT 92

10.1 Ethical Conduct of the Study 92

10.2 Informed Consent 92

10.3 Patient participation card 93

10.4 Insurance 93

11 PUBLICATION POLICY 94

APPENDIX A: ECOG PERFORMANCE STATUS 95

APPENDIX B: NCI CTCAE V4.03 96

APPENDIX C: LOCAL LABORATORY PARAMETERS 97

APPENDIX D: COCKCROFT-GAULT FORMULA 98

REFERENCES 99

LIST OF TABLES

Table 1: Anti-angiogenic and vascular disrupting agents in ovarian cancer trials (Sources: Citeline, Eskander and Tewari, 2014[x]) 33
Table 2: Summary of comparison of ALM201 with vascular disrupting agents 37
Table 3: Summary of comparison of ALM201 with anti-angiogenics 37
Table 4: PK properties of ALM201 in mouse, rat and dogs 38
Table 5: Starting dose (10 mg) against a range of patient weights: 40
Table 6: Maximum Dose Escalation Steps Permitted 50
Table 7: Volume of Blood Sampling 68
Table 8: Composition of ALM201 for Injection, 100 mg/mL 76
Table 9: Definition of PK Parameters 88
Table 10: Operating characteristics of a 3+3 cohort design 89

LIST OF FIGURES

Figure 1: Sequence of the FKBP-L protein with the identified 24 amino acid anti-angiogenic domain highlighted in red 36
Figure 2: ALM201 dose dependently inhibits three measurable parameters of aortic ring vessel outgrowth, namely maximum length, mean length and total vessel number. 38
Figure 3: Dose Escalation Plan 50

LIST OF ABBREVIATIONS

ADL	activity of daily living
AE	adverse event
ALT (SGPT)	alanine aminotransferase (serum glutamic pyruvic transaminase)
APTT	activated partial thromboplastin time
AST (SGOT)	aspartate aminotransferase (serum glutamic oxaloacetic transaminase)
AUC	area under the plasma concentration versus time curve
AUC_{0-t}	AUC from time zero to time t
$AUC_{0-\infty}$	the area under the concentration-time curve estimated from time zero to infinity as the sum of the two areas: AUC_{0-t} and AUCextrap, where AUC_{extrap} is calculated as C_t / λz
BAD	biologically active dose
BALB-c	albino laboratory-bred strain of house mouse
BP	blood pressure
CA	Competent Authority/ies
CA-125	cancer antigen-125
CEA	aarcinoembryonic antigen
CI	confidence interval
C_{inf}	the observed concentration at the end of the administration.
CIOMS	Council For International Organizations of Medical Sciences
CL	the systemic clearance calculated as: Dose/ $AUC_{0-\infty}$
C_{max}	maximum plasma concentration
C_{min}	minimum observed concentration
CR	complete response
CRC	Cohort Review Committee
CRF	case report form
CRO	contract research organisation
CSR	Clinical Study Report
CT	computed tomography
CTC	circulating tumour cells
CTCAE	Common Terminology Criteria for Adverse Events
DLT	dose limiting toxicity
DNA	deoxyribonucleic acid
ECG	electrocardiogram
ECOG	Eastern Cooperative Oncology Group
eCRF	Electronic Case Report Form
EDC	electronic data capture
EGF	epidermal growth factor
EU	European Union

FCS	fetal calf serum
FDA	Food & Drug Agency (US)
FFPE	formalin-fixed paraffin-embedded
FGF	fibroblast growth factor
GCP	Good Clinical Practice
GFR	glomerular filtration rate
GIST	gynecological Cancer Intergroup
GLP	Good Laboratory Practice
GMP	Good Manufacturing Practice
hCG	human chorionic gonadotropin
HED	human equivalent dose
IB	Investigator's Brochure
IC_{50}	half maximal inhibitory concentration
ICH	International Conference on Harmonisation of Technical Requirements for Medicinal Products for Human Use
IEC	Independent Ethics Committee
IMP	investigational medicinal product
IRB	Investigational Review Board
IV	Intravenous
MedDRA	Medical Dictionary for Regulatory Activities
MFD	maximum feasible dose
MRI	magnetic resonance imaging
MTD	maximum tolerated dose
NCI	National Cancer Institute
NOAEL	no observed adverse effect level
PBMC	peripheral blood mononuclear cells
PD	pharmacodynamic(s)
PD	progressive disease
PFGF	platelet-derived growth factor
PK	pharmacokinetic(s)
PO	per oral
PR	partial response
pRBC	packed red blood cells
PS	performance status
PSA	prostate-specific antigen
QTcF	QT interval corrected for Fridericia's formula
RECIST	Response Evaluation Criteria in Solid Tumours
SAE	serious adverse event
SAP	statistical analysis plan
SC	subcutaneous
SCID	severe combined immunodeficiency

SUSAR	Suspected Unexpected Serious Adverse Reaction
t½	half-life
t_{max}	time to reach maximum concentration
TMF	Trial Master File
UK	United Kingdom
ULN	upper limit of normal
VDA(s)	vascular disrupting agents
VEGF	vascular endothelial growth factor
Vss	the apparent volume of distribution at steady state calculated as: Dose/AUC x (AUMC/ $AUC_{0-\infty}$ - T/2) where T is the duration of intravenous injection
λ_z	the apparent terminal rate constant, estimated using the negative slope of the least square regression analysis of the log concentration versus time data for the terminal linear portion of the curve

1 INTRODUCTION

ALM201 is a 23-amino acid, linear peptide, under development by Almac Discovery for the treatment of cancers where there is a rationale for treatment with anti-angiogenic agents. ALM201 is derived from the human, endogenous protein, FKBP-L, which has inherent anti-angiogenic activity (Valentine AI, 2011[iii]). The sequence responsible for this activity was identified using truncated peptides from the protein and was found to be a 24 amino acid sequence near the N terminus. In order to improve stability of the peptide, the N-terminal glutamate has been removed, whilst retaining equivalent activity of the native peptide (termed AD-01). ALM201 is formulated as an aqueous solution containing 80mM sodium carbonate, 20mM Tris and 25mM sodium chloride, pH 6.5 for subcutaneous administration, and is supplied as a 100 mg/mL sterilised solution for SC injection.

1.1 Rationale for use

Angiogenesis is a key mechanism in the development and growth of tumours (Folkman, 2007[iv]; Oklu et al, 2010[v]). The understanding of the mechanism of angiogenesis has continued to improve and a number of biological and small molecule anti-angiogenic drugs have achieved approval as cancer therapies in colorectal cancer, glioblastoma, GIST (Gynaecological Cancer Intergroup), hepatocellular carcinoma, renal cell cancer, and neuroendocrine tumours; with anti-angiogenic agents being tested in clinical trials in many more cancer types. To date the majority of these approved therapies have targeted increased signalling through receptor tyrosine kinases and growth factor expression, particularly VEGF, and used inhibition of the pathways as their primary mechanism of action. Limited efficacy can be a significant problem and patients receiving tyrosine kinase inhibitors have a high rate of resistance after several cycles of therapy (Carmeliet and Jain, 2011[vi]).

Other agents identified as potential inhibitors of tumour development through targeting of existing blood vessel maturation and development are the vascular disrupting agents, including vinca alkaloids and combretastatins (Siemann et al, 2005[vii]). These agents have specific mechanisms of action that focus on the microtubule networks which drive migration and invasion of cancer and endothelial cells (Denekamp, 1982[viii]). Although these compounds can be highly potent, they have significant dose limiting toxicities (Hinnen et al, 2007[ix]).

ALM201 shows significant anti-tumour activity in a range of *in vitro* and *ex vivo* assays of angiogenesis and cell migration. Investigation of the mechanism of ALM201 has revealed that the peptide is internalised into CD44 expressing cells and targets microtubules. However, ALM201 has no effect on cell viability, cell cycle or cell proliferation making it an attractive agent to take into the clinic. Although this first-in-human study will explore safety, pharmacodynamics activity and possible anti-tumour activity of ALM201 in patients with advanced tumours, it is anticipated that ALM201 will most likely be used in a similar way to existing anti-angiogenic therapies, which are often given in combination with chemotherapy. Attacking the tumour cells via multiple mechanisms of action may increase the effectiveness of the therapy and lead to improved response rates and survival times.

1.2 Justification for treatment in ovarian cancer

The choice of ovarian cancer for the first clinical trial with ALM201 was made for three reasons:

1. the unmet medical need in advanced ovarian cancer;

2. the growing opinion that anti-angiogenic agents will make an impact on treatment of ovarian cancer, as evidenced by the approval of bevacizumab, successful trials with four other anti-angiogenic drugs and the number of other anti-angiogenic and vascular disrupting agents (VDAs) entering trials for ovarian cancer; and
3. the availability of a proprietary diagnostic that allows enrichment for ovarian cancer patients who may be likely to benefit from anti-angiogenic therapy.

According to the Cancer Research UK web site, ovarian cancer is the fifth most common cancer in women in the UK, comprising about 4% of all new cases of cancer in women, with a crude incidence rate of 22 new cases per 100,000 women. It is the fourth most common cause of cancer deaths in women. In 2011 there were 4,300 deaths in the UK; latest figures available for worldwide deaths are 140,000 in 2008. Although 5 year survival in the UK for a diagnosis of stage 1 ovarian cancer is around 90%, this drops to 20% for stage 3 and to 6% for stage 4. With around 55% of ovarian cancer patients being diagnosed at stage 4 or 5, there is a clear unmet medical need for a significant number of patients with late stage ovarian cancer.

To date there have been eight phase 3 trials where anti-angiogenic drugs met their primary end points. Four of these trials were of bevacizumab; the other four involved pazopanib, cediranib, trebananib and nintedanib (Eskander and Tewari, 2014[x], Table 1). A number of other anti-angiogenic and antivascular agents are in earlier stage trials (Table 1).

There is a growing consensus that anti-angiogenic therapy will become a major contributor to the treatment of ovarian cancer (Burger, 2011[xi], Hall et al, 2013[xii]). Although Table 1 lists a number of trials in progress, they cover a limited range of mechanisms. Seven out of 15 are related to VEGF signaling, two are specific for angiopoietin and three are tubulin binders with potential limitations from side effects. ALM201 is differentiated from these drugs by mechanism of action, breadth of effects against different growth factors (compared to targeting VEGF or angiopoietin systems specifically) and lack of cytotoxic side effects. The use of anti-angiogenic drugs in ovarian cancer either concurrently with chemotherapy, in maintenance after chemotherapy or as monotherapy is at an early stage and it is not yet clear which compounds or which mechanisms will be most effective. There is therefore justification to evaluate an anti-angiogenic agent with a novel mechanism of action and anticipated clean toxicology profile such as ALM201.

Table 1: Anti-angiogenic and vascular disrupting agents in ovarian cancer trials (Sources: Citeline, Eskander and Tewari, 2014[x])

Drug	Company	Mechanism	Development Stage for ovarian cancer
Antibodies and antibody-like therapies			
Bevacizumab	Roche	Anti-VEGF antibody	Approved in Europe<br>(Ph III trials: GOG 218, ICON7, OCEANS, AURELIA)
Trebananib	Amgen	Peptibody Fc fusion targeting Ang 1,2	Phase III (TRINOVA-1)
Ramucirumab	Lilly	Anti-VEGFR2 antibody	Phase II
TB-403	Thrombogenics/BioInvent	Anti-PIGF antibody	Phase II
DI-17E6	Merck	Anti-integrin antibody	Phase I
MED 3617	AstraZeneca	Anti-Ang2 antibody	Phase I
Tyrosine kinase inhibitors			
Pazopanib	GSK	B-raf, VEGFR1,2 PDGFR inhibitor	Pre-registration<br>Ph III trial (AGO-OVAR16)
Nintedanib	Boehringer Ingelheim	VEGFR2, PDGFR, FGFR inhibitor	Phase III (AGO-OVAR12/LUME-Ovar 1)
Cediranib	AstraZeneca	VEGFR, PDGFR, FGFR inhibitor	Phase III (ICON6)
Sunitinib	Pfizer	VEGFR, PDGFR, Raf inhibitor	Phase II

Drug	Company	Mechanism	Development Stage for ovarian cancer
Antibodies and antibody-like therapies			
Bevacizumab	Roche	Anti-VEGF antibody	Approved in Europe<br>(Ph III trials: GOG 218, ICON7, OCEANS, AURELIA)
Trebananib	Amgen	Peptibody Fc fusion targeting Ang 1,2	Phase III (TRINOVA-1)
Ramucirumab	Lilly	Anti-VEGFR2 antibody	Phase II
TB-403	Thrombogenics/BioInvent	Anti-PIGF antibody	Phase II
DI-17E6	Merck	Anti-integrin antibody	Phase I
MED 3617	AstraZeneca	Anti-Ang2 antibody	Phase I
Tyrosine kinase inhibitors			
Sorafenib	Bayer	VEGFR2,3, FLT3, PDGFR inhibitor	Phase II
Vascular disrupting agents			
Combretastatin A prodrug	Oxigene	Tubulin binding agent	Phase II
BNC105	Bionomics	Oral combretastatin A4, tubulin binder	Phase II

Drug	Company	Mechanism	Development Stage for ovarian cancer
Antibodies and antibody-like therapies			
Bevacizumab	Roche	Anti-VEGF antibody	Approved in Europe<br>(Ph III trials: GOG 218, ICON7, OCEANS, AURELIA)
Trebananib	Amgen	Peptibody Fc fusion targeting Ang 1,2	Phase III (TRINOVA-1)
Ramucirumab	Lilly	Anti-VEGFR2 antibody	Phase II
TB-403	Thrombogenics/BioInvent	Anti-PIGF antibody	Phase II
DI-17E6	Merck	Anti-integrin antibody	Phase I
MED 3617	AstraZeneca	Anti-Ang2 antibody	Phase I
Tyrosine kinase inhibitors			
Combretastatin A	Oxigene	Tubulin binding agent	Phase I
VB-111	VBL Therapeutics	Dual action anti-angiogenic and VDA	Phase II

There are five main histological subtypes of ovarian cancer differing in tissue of origin, stage of presentation, sensitivity to chemotherapy, survival and driver genetic mutations. The most aggressive of these subtypes are the high grade serous tumours. Gene expression profiling studies have suggested there are three major subgroups of high grade serous ovarian cancer, two of these have upregulation of angiogenic genes and one has angiogenic gene repression and immune gene upregulation (Gourley C et al, 2014[xiii]). A 63-gene classifier has been developed to identify the subgroup with immune gene upregulation and validated using samples from the ICON7 study (first line paclitaxel/carboplatin with/without bevacizumab). The validation study showed that when bevacizumab was added to chemotherapy, the immune subgroup had a worse PFS than with chemotherapy alone. In the angiogenic groups, there was a trend to improved PFS with the addition of bevacizumb but this did not reach significance.

In this clinical study the dose escalation will be conducted in eligible patients with any advanced solid tumour and the dose expansion in patients with ovarian cancer, selected using the gene signature test. The use of this gene signature test will ensure that those ovarian cancer patients who are less likely to benefit or who may have a worse outcome from anti-angiogenic therapy will be excluded.

This study of ALM201 in ovarian cancer is at the leading edge of clinical trials with anti-angiogenics. Results from eight phase III trials already completed with anti-angiogenics suggests support our hypothesis that there will be an effect in the general population of ovarian cancer patients and there is a path to patient selection within this tumour type, that could increase the chance of treatment benefit further.

1.3 Summary of Non-Clinical Development

ALM201 is a linear 23-amino acid peptide, which internalises into cells and binds to tubulin, preventing the formation of microtubules, which in turn results in the inability of the cell to migrate. The peptide was identified from the FKBP-L protein, when it was observed that the protein exhibited anti-angiogenic activity.

Figure 1: Sequence of the FKBP-L protein with the identified 24 amino acid anti-angiogenic domain highlighted in red

METPPVNTIGEKDTSQPQQEWEKNLRENLDSVI**QIRQQPRDPPTETLELEVSPDPAS**QILEHTQGAE
KLVAELEGDSHKSHGSTSQMPEALQASDLWYCPDGSFVKKIVIRGHGLDKPKLGS.C.CRVLALGFPF
GSGPPEGWTELTMGVGPWREETWGELIEKCLESMCQGEEAELQLPGHTGPPVGLTLASFTQGRDSW
ELETSEKEALAREERARGTELFRAGNPEGAARCYGRALRLLLTLPPPGPPER

Evaluation of the peptide suggests it does not behave as would be expected for either an antagonist or agonist of CD44. Migration studies using various agents which prevent cell internalisation have demonstrated that ALM201 requires to be internalised in order to be effective. Once inside cells it has been shown in-vitro that ALM201 binds to tubulin and inhibits microtubule formation as demonstrated by a tubulin activity assay, as well as cell staining and confocal microscopy.

Inhibition of migration at concentrations of 10^{-7} to 10^{-13} M has been shown in a wound scrape assay using HMEC-1, a human vascular endothelial cell-line, where a cell monolayer is grown, then wounded and the migration of cells into the wound space followed. The same cell line was used to show the inhibition of tubule formation (simulating new blood vessel growth) by ALM201 in a

Matrigel system across a concentration range 10^{-7} to 10^{-13} M. ALM201 at a concentration of 10^{-9}M was also found to inhibit invasion in a modified Boyden chamber assay when stimulated by VEGF, EGF, FGF, PFGF and FCS.

Despite marked inhibition of migration, invasion and tubule formation, ALM201 has been found to have no effect on cell proliferation or cell cycle in HMEC-1 cells across the same concentration range, stimulated with a range of growth factors, confirming that ALM201 does not have cytotoxic effects on cells. ALM201 also had no effect on cytokine release and T-cell proliferation in heparinised human blood alone or in combination with Staphylococcus Enterotoxin B at concentrations of 10^{-8} to 10^{-6}M.

ALM201 has been compared to several anti-angiogenic and vascular disrupting agents in the cell viability, tubulin polymerisation and cell-cycle assays. In addition, comparison of ALM201 with other anti-angiogenic agents was compared in the wound scrape assay. The results are shown in Tables 2 and Table 3 below.

Table 2: Summary of comparison of ALM201 with vascular disrupting agents

Assay	IC_{50} (nM)		
	**ALM201**	**Combretastatin**	**Vinblastine**
**Cell viability**	No effect	0.2	0.02
**Tubulin polymerisation**	0.1	360	502
**Cell cycle arrest**	No effect	Yes	Yes

Table 3: Summary of comparison of ALM201 with anti-angiogenics

Assay	IC_{50} (nM)		
	**ALM201**	**Bevacizumab**	**Sunitinib**
**Wound scrape**	0.009	59	8.9
**Proliferation**	No effect	158	1300
**Tube formation**	0.082	2400	4.4
**Tubulin polymerisation**	0.11	No effect	No effect

Across these assays ALM201 exhibited a profile which appears unique when compared to other anti-angiogenics. The peptide is effective at picomolar concentrations in all the *in vitro* assays, is not cytotoxic to cells, and inhibits the formation of microtubules in the biochemical assay. Taken together this suggests a potent, non-toxic effective anti-angiogenic activity *in vitro*.

The activity of ALM201 has been investigated using an *ex vivo* model of rat aorta, where sections are coated in a dome of Matrigel to support tubule out-growth over a 7-day period. ALM201 significantly inhibited the three main parameters of the assay; mean tubule out-growth, maximum tubule length and mean tubule length, with an IC_{50} in the range of 5-20 pM, depending on the parameter assessed.

Figure 2: ALM201 dose dependently inhibits three measurable parameters of aortic ring vessel outgrowth, namely maximum length, mean length and total vessel number.

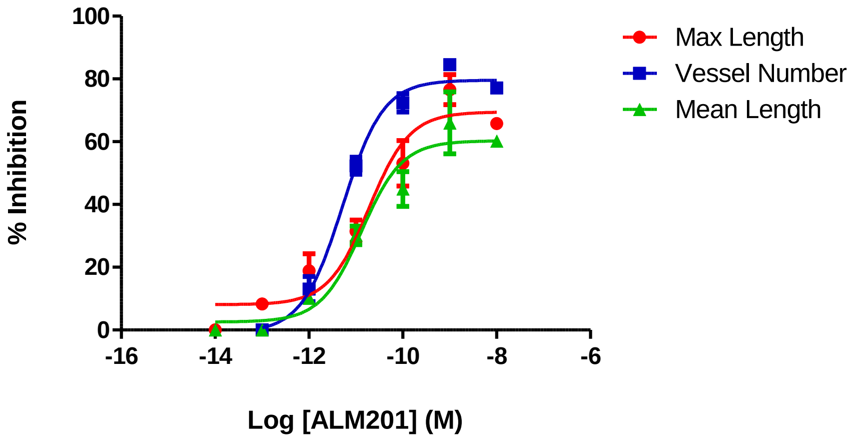

In vivo studies using a BALB-c SCID mouse xenograft model with DU145 and HT29 cell-lines, where the small tumours can be directly examined *in situ* using confocal and standard microscopy have shown that administration of ALM201 inhibits a range of markers of new blood vessel formation. Tumours were typically 1-2 mm at baseline and assessments taken over a 14-day period. Dose levels of 3.0 mg/kg/day and 0.3 mg/kg/day resulted in significant decreases in average vessel diameter, average segment length and vessel branch number.

Conventional xenograft growth delay studies in a number of xenograft cell lines (HT29, DU145 and MDA-231) confirm that administration of ALM201 at 0.3 mg/kg and 3.0 mg/kg given either daily or three times weekly resulted in significant inhibition of tumour size relative to vehicle controls.

The PK properties of ALM201 have been assessed in mouse (IV and SC), rat (IV and SC) and dogs (SC). In all species studied ALM201 has linear PK and a short half-life.

Table 4: PK properties of ALM201 in mouse, rat and dogs

	Half-life (hr)	Cmax (ng/mL)	Tmax (min)	AUC24 (ng.h/mL)
Mouse 3mg/kg SC	0.2	3477	10	2166*
Rat 5mg/kg SC	0.6	3400	30	4320
Dog 5mg/kg SC	2.4	2030	60	7300

*Figure is for AUC0-∞. All data is for male animals

There is a lack of correlation between the PK data for ALM201, with its relatively short plasma half-life and the activity in *in vitro* and *in vivo* assays. The dose regimen selected (dosing on Days 1-5 weekly) reflects this, and may be amended during the clinical study to optimise exposure or dose effect levels.

Five (5) toxicology studies have been completed:

(i) a non-GMP dose ranging study in rats, dose range 5-50 mg/kg/day;

(ii) a non-GLP dose ranging study in dogs, dose range 15-50 mg/kg/day;

(iii) a GLP 28-day study in rats, dose range 5-50 mg/kg/day;

(iv) a GLP 28-day study in dogs, dose range 5-50 mg/kg/day;

(v) a non-GLP wound healing study in rats, dose range 5-50 mg/kg/day.

In the rat dose range finding study, administration of doses of up to 50 mg/kg/day for 14 days were assessed. ALM201 was well tolerated at all doses and the No Observable Adverse Effect Level (NOAEL) was considered to be 50 mg/kg/day. No mortality, body weight loss or clinical signs or symptoms were reported for all dose levels. Histological analysis of a limited panel of tissues showed a low level of cell proliferation in the lymph nodes adjacent to the injection site, which was more prevalent in females, although changes generally remained within the normal range, there were no other histological findings.

The dose range finding study in the beagle dog used a staircase design, assessing doses of 15 mg/kg/day and 50 mg/kg/day for 14 days. ALM201 was well tolerated in both dose groups and the NOAEL was considered to be 50 mg/kg/day. Findings were limited to minor changes in clinical pathology, consisting of slightly lower red cell parameters for the 50 mg/kg/day group and one animal in the 15 mg/kg/day group, and were considered to be of minor toxicological importance in the absence of other treatment related changes. Histological analysis showed slight enlargement of germinal centres in mandibular lymph nodes for the 50 mg/kg/day animals, although changes were still generally within the normal range and not considered adverse.

GLP toxicology studies of 28 days duration plus recovery period have been completed in rat and dog, at doses of 5, 15 and 50 mg/kg/day. There were no treatment-related changes observed in bodyweight, food consumption, ophthalmoscopy, ECG, blood pressure, respiration rate, body temperature, haematology, urinalysis, organ weights and Irwin screen (rats only). In the rat study there was a minimal/slight increase in germinal centre incidence in some females, which was not considered adverse. In the dog study there were no microscopic changes noted that were considered to be treatment-related. Local tolerability was good in both the dog and rat studies. The NOAEL is 50 mg/kg/day in both studies.

A wound healing toxicology study has been completed in the rat, at doses of 5 and 50 mg/kg/day for up to 18 days. No effect on wound closure or tensile strength was observed in ALM201 treated groups compared to vehicle controls.

Please refer to the latest edition of the Investigator brochure (IB) for full details of non-clinical studies with ALM201.

1.4 Summary of Clinical Exposure

Study ALM201/0001 is a first-in-human study of ALM201.

1.5 Rationale for Dose Selection

Based on GLP, 28-day, repeat dose toxicology studies in the dog and rat, the NOAEL for ALM201 was not defined at the maximum dose evaluated of 50 mg/kg. Therefore, based on standard allometric scaling based on the FDA guidelines (FDA, 2005[xiv]), it is predicted that a human equivalent dose (HED) range of 8–28 mg/kg could be given without any drug-related adverse effects. Assuming a starting dose calculation based on a 10-fold safety margin in the most conservative model (the rat); the starting dose would be 0.8 mg/kg.

Due to the challenges of the ALM201 liquid formulation for SC administration (limited administration volume), a flat dose has been recommended at each dose level, based on a 70 kg patient. Based on this patient weight, a dose of 0.8 mg/kg would translate to a 56 mg dose. The starting dose selected for this study is 10 mg, a factor of 56 less than the highest HED in the rat.

Table 5: Starting dose (10 mg) against a range of patient weights:

Patient Weight	Resultant Dose	Adjustment from HED (based on rat)
50 kg	0.2 mg/kg	40X less
70 kg	0.14 mg/kg	56X less
120 kg	0.08 mg/kg	96X less

Looking at biological effect, *in vivo* data in the rat xenograft model describes significant anti-tumour activity seen at 3 mg/kg, with no activity seen at 0.3 mg/kg. The HED of the rat biologically effective dose of 3 mg/kg would therefore be 0.5 mg/kg. Assuming a 70 kg human, this gives an equivalent dose of 35 mg. Thus a starting dose of 10 mg would have a safety factor of 3.5.

The proposal to utilise an accelerated titration model following the principles discussed by Simon and colleagues (Simon, et al. 1997[xv]) is considered appropriate given the conservative dose range proposed for this trial with respect to the lack of significant toxicity and identification of a NOAEL, as well as the anticipated lack of accumulation of this peptide agent.

1.6 Safety Guidance for Investigators

Investigators should refer to the latest edition of the Investigator Brochure (IB) for a full review of the potential risks associated with treatment with ALM201 and details of expected adverse events.

2 STUDY OBJECTIVES

The primary objectives of this study are to characterize the safety and tolerability of ALM201 and to identify a recommended phase 2 dose and schedule of administration of ALM201 in patients with advanced ovarian cancer.

Objective	Endpoint(s) (Assessment)
Primary:	
• To characterise the safety and tolerability of ALM201 (Parts 1 and 2)	• Ongoing evaluation of AEs during treatment and follow up; evaluation of DLT during Cycle 1 (Part 1 only)
• To identify a recommended phase 2 dose and schedule of ALM201 (Part 2 only)	• Safety, PK, PD and tumour response assessments
Secondary	
• To establish the pharmacokinetic profile of ALM201	• Assessment of pharmacokinetic variables (including C_{max}, C_{min}, AUC)
• To assess anti-tumour activity	• Tumour response assessment by RECIST 1.1 (Eisenhauer et al, 2009[i]) and/or other relevant response assessments for tumour types enrolled
• To assess anti-tumour activity in a biomarker-enriched group of patients with advanced ovarian cancer (Part 2 only)	• Tumour response assessment by RECIST 1.1(Eisenhauer et al, 2009[i])
Exploratory	
• To assess relevant tumour biomarkers and the pharmacodynamic activity of ALM201	• Assessment of relevant tumour biomarkers and markers of ALM201 activity<br>○ in archival and/or fresh tumour biopsy material e.g. CD44, FKBPL, CD31, pFAK, pHer2, ITGA5, CA-125, ER, PR, angiogenesis signature and other relevant or exploratory biomarkers as appropriate for tumour type<br>○ in blood e.g. RASSF1 methylation, and other relevant or exploratory biomarkers appropriate for tumour type<br>○ in ascites (Part 2 only) e.g. stem cell count, and other relevant or exploratory biomarkers appropriate for ovarian cancer

3 SELECTION CRITERIA

Part 1 of the study will enrol patients with advanced solid tumours in whom treatment with an anti-angiogenic agent is appropriate. Part 2 will enrol patients with ovarian cancer, screened using an angiogenesis gene signature, that have failed to respond to, or have relapsed following standard therapy.

3.1 Inclusion Criteria

Part 1 will enrol patients with advanced solid tumours in whom treatment with an anti-angiogenic agent is appropriate. Part 2 will enrol patients with ovarian cancer, screened using an angiogenesis gene signature, that have failed to respond to, or have relapsed following standard therapy.

(i) Part 1 Specific Inclusion Criterion

1. Patients with histologically and/or cytologically confirmed advanced solid tumour for whom no standard effective therapy is available or felt likely to be of limited efficacy and in whom a rationale for use of an anti-angiogenic treatment approach exists. Note: *Previous use of anti-angiogenic therapy is allowed if tolerated*

(ii) Part 2 Specific Inclusion Criterion

2. Patients with advanced ovarian cancer, who are intolerant of or whose tumour is resistant to platinums and who have failed to respond to, or have relapsed following, standard therapy and whose tumour has a proangiogenic profile as assessed by the angiogenesis gene signature test. Note: *Previous use of anti-angiogenic therapy is allowed if tolerated.*

(iii) General Inclusion Criteria for all Patients

3. Adult patients defined by age ≥16 years at time of consent.
4. Evaluable disease, either measurable on imaging, or with informative tumour marker(s), as assessed by RECIST 1.1 or other relevant response assessment criteria for tumour type.
5. Recovery from previous treatment to baseline or CTCAE ≤ grade 1, as determined by CTCAE v4.03 criteria (Appendix B), of reversible toxicities related to prior treatment, with the exception of alopecia, lymphopenia, other non-clinically significant adverse events; recovery from previous radiotherapy other than residual cutaneous effects or stable < Grade 2 gastrointestinal toxicity; complete recovery from surgery other than stable < Grade 2 toxicity.
6. Eastern Collaborative Oncology Group (ECOG) Performance Status (PS) of 0 or 1 (Appendix A).
7. Laboratory values at Screening:
    - Absolute neutrophil count ≥1.5 x 10^9/L without colony stimulating factor support;
    - Platelets ≥100 x 10^9/L;
    - Haemoglobin ≥9 g/dL (not transfusion dependent);
    - Total bilirubin <1.5 times the ULN (unless due to Gilbert's syndrome where it should be

≤ 2.5 x ULN);

- AST (SGOT) ≤2.5 times the ULN; ALT (SGPT) ≤2.5 times the ULN; ≤5 x ULN for patients with advanced solid tumours with liver metastases; patients with confirmed bony metastases will be permitted on study with isolated elevations in ALP <5 times the ULN;
- Serum creatinine ≤ 1.5 x ULN or estimated glomerular filtration rate (GFR) of >50 mL/min based on the Cockcroft-Gault formula (Appendix D);
- Normal coagulation (elevated INR, prothrombin time or APTT ≤ 1.3 x ULN range acceptable);
- Urine protein ≤2+ (as measured by dipstick).

8. Negative urine or blood human chorionic gonadotropin (hCG) test during Screening and within 7 days of Cycle 1, Day 1 in women of childbearing potential (defined as women ≤ 50 years of age or history of amenorrhea for ≤ 12 months prior to study entry). Sexually active male and female patients of childbearing potential must agree to use an effective method of birth control e.g. barrier methods with spermicides, oral or parenteral contraceptives and/or intrauterine devices, during the entire duration of the study and for 6 months after final administration of ALM201. Note that sterility in female patients must be confirmed in the patients' medical records and can be defined as any of the following: surgical hysterectomy with bilateral oophorectomy, natural menopause with menses >1 year ago; radiation induced oophorectomy with last menses >1 year ago; chemotherapy induced menopause with 1 year interval since last menses.
9. Ability to give written, informed consent prior to any study-specific Screening procedures, with the understanding that the consent may be withdrawn by the patient at any time without prejudice.
10. Patient is capable of understanding the protocol requirements, is willing and able to comply with the study protocol procedures, and has signed the informed consent document.

3.2 Exclusion Criteria

1. History of inability to tolerate anti-angiogenic therapies e.g. increased blood pressure (BP), proteinuria, prior thromboembolic events.
2. Previous history of bowel obstruction, clinical evidence of gastro-intestinal obstruction, large burden of peritoneal disease or evidence of bowel involvement on computed tomography.
3. Patents has received:

   a) any chemotherapy regimens (including investigational agents) with delayed toxicity within 4 weeks (6 weeks for prior nitrosourea or mitomycin C) of Cycle 1, Day 1, or received chemotherapy regimens given continuously or on a weekly basis which have limited potential for delayed toxicity within 2 weeks of Cycle 1, Day 1.

   b) radiotherapy, immunotherapy or biological agents (includes investigational agents) within 4 weeks of Cycle 1, Day 1. Localised palliative radiotherapy is permitted for symptom control.
4. Documented, symptomatic or uncontrolled intracranial metastases or primary intracerebral tumours.

5. Cancer with leptomeningeal involvement.
6. On therapeutic anti-coagulation (aspirin dosing ≤100 mg per oral (PO) daily allowed).
7. Previous malignancy, except for non-basal-cell carcinoma of skin or carcinoma-in-situ of the uterine cervix, unless the tumour was treated with curative intent more than 2 years prior to study entry.
8. History of clinically significant cardiac condition, including uncontrolled hypertension (BP >140/90 mmHg, despite medical therapy); left ventricular systolic dysfunction (ejection fraction (<55 %) on echocardiography) with or without heart failure symptoms; history of an ischaemic cardiac event within 3 months of study entry (myocardial infarction, acute coronary syndrome); QT interval prolongation (QTcF, Fridericia's Correction of >450 ms on screening 12-lead ECG); clinically significant cardiac arrhythmia within 3 months of study entry. Note: *ventricular tachycardia, ventricular fibrillation, supraventricular tachycardia, atrial fibrillation without adequate heart rate control, atrial fibrillation with adequate heart rate control with or without medication or other treatment, are not an exclusion.*
9. Known human immunodeficiency virus positivity.
10. Active hepatitis B or C or other active liver disease (other than malignancy).
11. Any active, clinically significant, viral, bacterial, or systemic fungal infection within 4 weeks prior to Cycle 1, Day 1.
12. Any evidence of severe or uncontrolled systemic conditions or any other issues which make it undesirable for the patient to participate in the study or which could jeopardize compliance with the protocol.

3.3 Patient Withdrawal or Discontinuation

Patients may withdraw from the study at any time without stating a reason and without prejudice to further treatment. A Final Study Visit should be performed 30 +/-3 days after the last dose of ALM201 to enable follow up safety assessments and further tumour assessment where required.

The Investigator may withdraw a patient from the study and discontinue study treatment and assessments at any time. Example reasons for discontinuing a patient from this study are:

- Disease progression.
- The patient experiences a toxicity, including those necessitating a dose delay of >14 days (Section 4.2.1), where the re-introduction of ALM201 (including a dose reduction of ALM201), is not considered suitable. Exceptions may be considered where the toxicity is not considered to be ALM201 treatment-related.
- Other toxicities or events, unrelated to ALM201, that would, in the Investigator's opinion, prevent the patient from continuing on this trial.

- Protocol non-compliance. (All documentation concerning the patient must be as complete as possible. Withdrawals due to non-attendance of study visits must be followed-up by the Investigator to obtain the reason for where possible).

- Patient withdraws consent to participate in the study.

- Patient becomes pregnant (see section 7.4.3)

The Sponsor reserves the right to request the withdrawal of a patient due to protocol violation or other significant reason. Patients who experience a toxicity event which qualifies as a DLT, may continue to receive ALM201 if considered safe to do so and where continued treatment is considered by the Investigator to be in the patient's best interests. The decision to continue treatment will be reviewed by the CRC (Section 8.1) and will involve an adjustment (de-escalation) in dose or dose schedule.

3.4 Replacement of Non-evaluable Patients

In Part 1, patients will be replaced if they are not considered evaluable for DLT during Cycle 1. As a general rule during Part 1, patients will be considered evaluable for DLT during Cycle 1 if they receive at least 80% (i.e. 12 of 15 planned doses) of their intended dose, unless this is due to ALM201-related toxicity. However, this will be reviewed by the CRC on a case-by-case basis. For example, based on safety and PK data available, the CRC may agree that on a weekday dosing schedule, missing 1 dose per week on the 21-day dosing cycle is acceptable; whereas, missing 3 consecutive doses is not. Furthermore, the ability to evaluate a patient who misses more than 80% of their intended dose due to a toxicity considered to be related to ALM201, but which is not ultimately classed as a DLT, will be reviewed on a case-by-case basis. Patients who withdraw from the study or discontinue treatment after completion of the first treatment cycle will not be replaced.

In Part 2, patients may be replaced upon consideration of their reason for withdrawal e.g. not received at least 80% of their intended dose level, not evaluable for safety assessment (at least during Cycle 1), or not undergone at least one follow up tumour assessment. During Part 2, the CRC will continue to assess tolerability of ALM201 at the MTD, MFD or BAD(s) selected for further evaluation, and will advise on individual dose de-escalation steps where necessary (Section 8.1).

3.5 Procedures for Discontinuation

When a patient withdraws from the study, the reason for withdrawal should be sought where possible and recorded in the patient file and the Case Report Form (CRF). Every effort will be made to complete the Final Study Visit.

3.6 Study or Site Termination

If the Sponsor or their representatives, Investigator, or Competent Authority (CA) discover conditions during the study that indicate that the study or site involvement should be terminated, this action may be taken after appropriate consultation with the Sponsor and the Investigator. Conditions that may warrant termination of the study include, but are not limited to:

- The discovery of an unexpected, serious, unacceptable risk to patients enrolled in the study.

- The decision on the part of the Sponsor to suspend or discontinue testing, evaluation, or development of the study drug (note that this may be due to limited supply of investigational medicinal product (IMP) after patients have received 8 cycles of treatment).

Conditions that may warrant termination of a study site include, but are not limited to:

- Failure of an Investigator to comply with pertinent clinical trial regulations.
- Submission of knowingly false information from the research facility to the Sponsor, study monitor, or CA.

- Insufficient adherence to protocol requirements.

Study termination and follow-up will be performed in accordance with applicable local regulations.

4. INVESTIGATIONAL PLAN

4.1 Study Design

This is a Phase 1, open-label, dose escalation study of the safety, tolerability, and PK of ALM201. The study will commence by enrolling patients with advanced solid tumours in whom treatment with an anti-angiogenic agent is appropriate (Part 1). Eligible participants will be enrolled in sequential, dose escalating cohorts treated with ALM201, given as a SC injection while being monitored for safety and DLTs. Part 2 will commence once a dose level of interest has been selected for further evaluation in patients with advanced ovarian cancer.

All dose escalation decisions will be made by a CRC who shall convene to review all available AE, PK, PD and relevant patient data (Section 8.1). The CRC will be composed of the trial investigators, a patient representative, sponsor and CRO representatives, plus the study Medical Monitor. Additional experts may be invited to support the review of the data as required e.g. a pharmacokineticist. All data reviewed, CRC discussions and agreed dose escalation recommendations will be minuted.

Dose levels will not be weight-adjusted and the starting dose for the study will be 10 mg ALM201 given on Days 1-5, 8-12 and 15-19 every 21 days i.e. weekday dosing. Dose increments will not exceed 100% and will be guided by safety data observed during Cycle 1, as well as on-going assessment of safety beyond Cycle 1 in earlier cohorts, plus PK and PD data as available. Every new dose cohort in Part 1 will be evaluated for the occurrence of a DLT during Cycle 1 of treatment (Section 4.2.1).

The study will commence with an accelerated dose escalation schedule and enrol 1 patient into a cohort with follow up for AEs and DLT during Cycle 1. The CRC will review all patient safety data at the end of Cycle 1 for each cohort, and permit 100% dose escalation steps to the next dose where there are no safety concerns. Where the CRC suspects that drug-related events have occurred that could progress to DLT upon further dose escalation e.g. clinically significant NCI CTCAE Grade 2 events considered to be related to ALM201, they will confirm that future cohorts must enrol 3 patients in order to more thoroughly evaluate potential drug-related adverse events. The CRC may also advise that the current dose level under evaluation be expanded to 3 patients prior to further dose escalation, in order to help inform the next dose escalation step. Dose escalation of 3-patient cohorts will proceed according to the scheme presented in Section 4.2. Note that there will always be stagger of at least 1 week between dosing the first and subsequent patients in a new dose cohort. The CRC may request there be a further or prolonged stagger introduced depending on the nature of adverse events observed to date.

Patients will have scheduled site visits on every dosing day of the first cycle, then on Days 1, 8 and 15 of Cycles 2-4. From Cycle 5 onwards, they are only required to visit the clinic on Day 1 of each cycle. ALM201 administration can be given at home on all other days.

Safety assessments will include physical examination, vital signs, biochemistry and haematology laboratory screens, plus immunogenicity testing (see Schedule of Study Assessments). Adverse events will also be noted at every clinical visit and recorded at least every week. For all administrations in hospital, the patients must wait for at least 60 minutes from the time of the

ALM201 injection for observation and repeat vital signs. These precautions are in case of emergent evidence of immunogenicity in Cycle 1, and at other times after the weekend break in dosing.

Tumour assessment by imaging (CT scan or MRI scan as appropriate for tumour type) will be assessed in all patients at Screening and after every 2 cycles of treatment (i.e. every 6 weeks) during Cycles 1–8 (first 24 weeks), and then after every 4 cycles of treatment (i.e. every 12 weeks) from Cycle 9 onwards. Scans may be performed at other times as clinically indicated.

Tumour assessment by informative tumour markers where relevant for tumour type e.g. GCIG criteria for CA125 (Rustin, et al. 2011[xvi]), PSA, CEA or CA19-9, will be assessed in all patients at Screening and after every 2 cycles of treatment during treatment (i.e. every 6 weeks). Tumour markers may be performed at other times as clinically indicated.

A PK profile for ALM201 will be taken on Days 1, 3 and 18 of Cycle 1 and on Day 18 of Cycles 2, 4, 6 and 8. Pre-dose samples will also be taken on Cycles 2-8 on Day 1.

All patients will be asked to provide consent for access to archived tumour tissue where available, and where possible, fresh biopsies will be taken pre-dose and again during the study where the patient has a documented tumour response and/or at the point of disease progression to allow for potential biomarker and pharmacodynamic assessment. In Part 2, access to a tumour biopsy sample will be required to confirm each patient's eligibility for the study (see Inclusion Criterion 2). A fresh biopsy will be preferable for this purpose; however, where it is not possible to obtain this, confirmation of the angiogenesis signature may be performed on archived tumour tissue during Screening. In Part 2, there will also be additional biomarker/PD assessments in blood and ascites. In both parts of the study, any remaining samples obtained for biomarker/PD assessment will be retained for potential future analysis.

Patients will be withdrawn from the study at the point of receiving their last dose of ALM201. However, all patients will be asked to participate in a protocolled follow up assessment 4 weeks after their last dose for safety assessments. Furthermore, patients will continue to be followed up either in the study clinic or by telephone contact every 8 weeks (see Schedule of Study Assessments) for up to approximately 2 years to check their disease (survival) status.

For the purpose of this study, a full treatment course of ALM201 will considered to be 8 cycles. However, patients who respond to ALM201 treatment during the trial may be suitable to receive further cycles of treatment with the IMP where recommended by the investigator and subject to ALM201 availability. Such patients will continue to be followed up for safety and tumour evaluation (Schedule of Study Assessments).

4.2 DLT Evaluation and Dose Escalation Scheme

The CRC will agree on the next appropriate dose escalation step for each cohort primarily based on DLT evaluation during Cycle 1 of the current cohort. Only events occurring during the first cycle of treatment will be considered for DLT determination; however, there will be on-going evaluation of AEs in subsequent treatment cycles which will be discussed during the cohort review process. Clinically significant events thought to be potentially related to ALM201, or trends in adverse events

seen in subsequent dosing cycles will be taken into account when considering future dose escalation steps and dose administration schedules.

Upon occurrence of the first DLT in any cohort, additional patients will be added to that cohort so that up to a total of 6 can be evaluated. Once expansion to a 6-patient cohort has been recommended due to the identification of a DLT, escalation to the next 3-patient cohort will only occur when all patients in the expanded cohort have completed their first cycle of ALM201 and no more than 1 DLT has occurred. If 2 or more DLTs occur in an expanded cohort, DLT is established and the next lower dose level will be declared the MTD.

The highest dose where ≤1 DLT is seen in 3 or 6 patients will be termed the MTD. Note that intermediate dose levels may be explored below the dose level where ≥2 DLTs were seen, in order to identify the maximum dose which may be adequately tolerated. The CRC may also specify an appropriate recruitment stagger to be followed during cohort expansion for DLT evaluation depending on the nature of the DLT seen and the considered risk to patients.

In the case where an MTD is not established, the maximum feasible single dose which may be administered will be dictated by the formulation of ALM201 and the maximum volume for SC administration i.e. 3 x 1 mL injections, giving an upper limit of a 300 mg dose. Should this dose be reached without the need to de-escalate due to DLT, it will be termed the MFD.

Based on PD data evaluation in conjunction with on-going safety and PK data, the CRC may also identify a BAD for further exploration in Part 2 of the study.

The schematic below illustrates the dose escalation plan for the protocol based on safety data evaluation.

Figure 3: Dose Escalation Plan

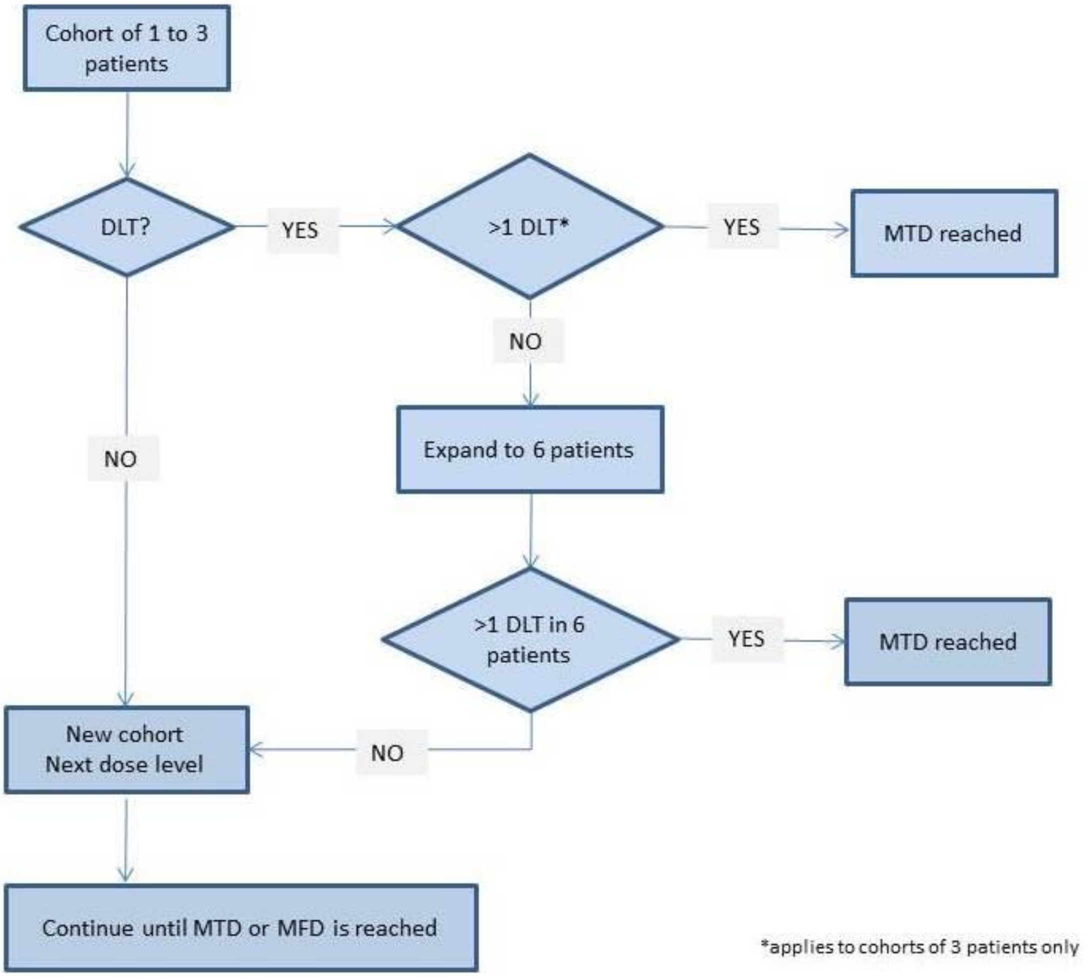

In the absence of DLT or suggestion of ALM201-related events which would lead to more cautious dose escalation, the following table presents a hypothesised dose escalation plan:

Table 6: Maximum Dose Escalation Steps Permitted

Cohort	Escalation Step	Dose level
**1 (Starting Dose)**	-	10 mg
**2**	(2X starting dose)	20 mg
**3**	(4X starting dose)	40 mg
**4**	(8X starting dose)	80 mg
**5**	(16X starting dose)	160 mg
**6 (MFD)**	(30X starting dose)	300 mg

The dose may be doubled in sequential cohorts where the CRC consider it appropriate to do so, based on on-going evaluation of DLTs and adverse event data. Dose escalation decisions will also take available PK and PD data into consideration.

In the case where a potentially significant toxicity occurs, or a trend in toxicities is seen, considered to be related to treatment with ALM201 which could potentially be a precursor to a clinically significant toxicity event; subsequent dose escalation steps will be more conservative and will not exceed 50% of the previous dose. This restriction may be reversed where there is no suggestion of potentially clinical significant toxicity in subsequent cohort(s). In the case where a single DLT event is observed, the next dose escalation step will not exceed 33% of the previous dose. However, where there are no further events seen in the next cohort; the CRC may allow future dose escalation steps of up to 50%.

Based on on-going safety, PK and PD data evaluation, the CRC may also recommend dose de-escalation steps or adjustments in the dose administration schedule of ALM201. Upon review of available safety, PK and PD data, the CRC may recommend that an alternative dose administration schedule of ALM201 be explored. Less intense dose administration schedules will be permitted as long as the dose under evaluation does not exceed the next permitted escalation step. Given the starting dose administration schedule is week-day dosing i.e. D1-5, D8-12, D15-19, on a 21 day cycle, a more dose intense dosing schedule is unlikely. However, should the CRC consider this reasonable to explore, the same dose escalation rules will apply and pharmacokinetic modelling, in conjunction with the safety data obtained to date, may be used to inform the appropriate dose level for a more dose intense dose schedule.

Note that the MTD or MFD need not be confirmed for the original dose administration schedule prior to the CRC recommending the investigation of an alternative dose administration schedule. Multiple dose escalation tracks may be followed if more than one dose level is considered relevant to explore as long as the dose escalation rules are followed e.g. the CRC may recommend that the alternative dose administration schedule replaces a schedule or is explored in addition to another schedule.

The CRC may also recommend that an MTD, MFD or BAD be assessed in a cohort of 6 patients in Part 1 where only 3 patients have received this dose level to date, prior to recommending the enrolment of an enrichment cohort to receive this dose level in Part 2.

4.2.1 Dose Limiting Toxicities

Safety evaluations will be conducted weekly during each treatment cycle, with DLT assessed during Cycle 1 only. All events and suspected DLTs will be graded according to the CTCAE, version 4.03.

A DLT is defined as a Grade 3 or 4 adverse event that, in the opinion of the CRC, is likely to be related to ALM201 and represents a clinically significant hazard to the subject. Qualifying DLT events must be considered to be clinically relevant e.g. in duration, apparent reversibility, required management, and upon consideration of the patient’s medical history and/or concomitant medications. DLT events must also be evaluated in terms of what is considered to be an appropriate next escalation step: in the case where the CRC agree that an escalation step of approximately 33% or lower is merited, the toxicity of concern should be declared a DLT.

Examples of exceptions that will be considered by the CRC are as follows:

- Grade 3 or 4 laboratory abnormalities, which resolve spontaneously or can be corrected with appropriate treatment (such as electrolytes) e.g. an event returns to baseline or to grade 1 or less prior to the next administration);

- Symptomatic adverse events, such as nausea, vomiting and diarrhea, if they can be reduced to less than Grade 3 with standard supportive measures, such as anti-emetics and anti-diarrhoeals within 72 hours.

In order to be evaluable for DLT assessment, a patient must have received at least 80% of their scheduled doses (e.g. 12 of the 15), unless this lack of compliance is due to ALM201-related toxicity. DLT events must therefore be considered in terms of inability to administer the planned Cycle 1 dose administration schedule, and in such cases a dose delay of more than 14 days due to a toxicity event considered related to ALM201 will be considered to be a DLT (Section 4.4.1).

If any event meets more than one category of DLT, it may be classified as one event for consideration of the next dose escalation step.

4.3 Expansion at MTD, MFD or BAD

Once an MTD, MFD and/or BAD has been established for a given dose and schedule in Part 1, Part 2 may commence. Part 2 will involve up to an additional 36 patients with advanced ovarian cancer. During Part 2 of the study there will be on-going evaluation of safety and the opportunity to obtain preliminary anti-tumour activity at each dose level selected in the ovarian cancer population. Any dose adjustments in Part 2 will only be dose de-escalations (based on a reduction of the unit dose given or a less frequent administration schedule), driven by each patient's tolerability of ALM201.

4.4 Dose Adjustments

4.4.1 Dose Delay

Patients may delay their next dose by up to 14 days on one occasion during the study to allow a toxicity (drug related or otherwise) to return to <Grade 2 or acceptable baseline levels. Any dose that is delayed should be given when the toxicity has adequately resolved within this treatment window utilizing the intended CRF pages for the original protocolled visit. If any dose cannot be given within 14 days of the scheduled date due to toxicity, and dose reduction is not considered suitable, the patient will be withdrawn from the study. Exceptions will be considered for events not deemed to be treatment-related (Section 3.3). In such exceptional cases, the sponsor will advise on how to complete the CRF on a case by case basis.

Clinically significant toxicity events considered related to ALM201 which lead to a dose delay of more than 14 days during Cycle 1, will be evaluated for DLT (Section 4.2.1).

4.4.2 Dose Reduction

The CRC may advise on a dose reduction step based on safety and on-going data evaluation from each cohort (Section 8.1). Intra-patient dose reduction of ALM201 is also permitted in this study. Investigators, in conjunction with approval by the CRC, may choose to administer a lower dose which has previously been assessed during the trial (or intermediate dose), to an individual patient. Note that this approval may be expedited outside the regular cohort review committee meetings. All intra-patient dose reductions must be justified in writing as a preferable option over patient withdrawal. In order to allow the evaluation of a possible DLT (Section 4.2.1), a dose delay of up to 14 days must be considered prior to any dose reduction.

Where 2 or more DLTs are confirmed in a particular cohort, the CRC may also recommend a dose de-escalation step to an intermediate dose, rather than to the previous dose given. Again, this decision will be based on thorough evaluation of all safety data and on-going supporting data available from the trial, taking into account the magnitude of the prior escalation step(s) and prior tolerability of ALM201.

4.4.3 Intra-Patient Dose Escalation

Intra-patient dose escalation will not be permitted in this study.

4.5 Duration of treatment and treatment beyond Cycle 8

The main study will permit a maximum of 8 three-weekly treatment cycles (or approximately 6 months of treatment). Patients who are seen to potentially be benefiting from treatment i.e. patients whose disease has not progressed and who have not been withdrawn from therapy due to toxicity, will be eligible to continue to receive additional cycles of ALM201 where this is recommended by their study physician, subject to availability of ALM201. Such patients will continue to be followed up for compliance, toxicity and continued response (Schedule of Study Assessments).

5. STUDY SCHEDULE

Patients will attend the clinic for Screening assessments up to 28 days before receiving the first dose of ALM201.

The study will commence with a dose schedule of ALM201 given as an SC injection on Days 1-5, 8-12 and 15-19 of a 21-day cycle. Patients will be required to visit the study sites on each dosing day during Cycle 1, on Days 1, 8 and 15 during Cycles 2-4, then on Day 1 only for each cycle thereafter. All other ALM201 administrations may be given at home.

A tolerance of +/-1 day will be permissible for all study visits, except the final study visit, which will have a tolerance of +/-3 days (this does not take into account any required dose delays). A tolerance of -1 day is permitted for all assessments relative to the study visit, unless specified otherwise (see footnotes to Schedule of Study Assessments).

Additional assessments may be conducted as clinically indicated.

5.1 Schedule of Study Assessments (i)

Below is a summary of study assessments described by visit for Cycles 1-8 based on the starting dose administration schedule. Please also refer to the Schedule of Study Assessments - Table (i) and their associated footnotes.

Cycle 1, Screening (up to 28 days prior to Cycle 1, Day 1)

~ Informed consent (including optional consent for fresh biopsy samples for biomarker/PD assessments and blood sample for biomarker germ-line deoxyribonucleic acid (DNA) testing)
~ Demographics
~ Medical history
~ Concomitant medication
~ Inclusion/exclusion checks
~ ECOG Performance Status
~ Full physical examination (including height and weight)
~ Vital signs
~ ECG
~ Echocardiogram
~ Clinical Chemistry
~ Haematology
~ Coagulation
~ Urinalysis
~ Serum pregnancy test (if applicable)
~ Serum tumour markers if appropriate (e.g. CA-125, PSA, CEA, CA19-9)
~ Tumour assessment by appropriate imaging
~ Biomarker/PD assessment – fresh biopsy (optional)
~ Biomarker/PD assessment – blood
~ Biomarker/PD assessment – ascites (if applicable in Part 2)
~ Biomarker testing – blood sample to obtain germ-line DNA

Cycle 1, Day 1

~ Full physical examination
~ Vital signs (pre-dose and approximately 1 hour after ALM201 administration)
~ ECG (pre-dose and approximately 30 minutes after ALM201 administration)
~ Clinical chemistry
~ Haematology
~ Coagulation
~ Urinalysis (include urine pregnancy test if applicable; no need to retest if within 7 days of last evaluation)
~ Tumour assessment (serum marker as appropriate for tumour type)
~ Immunogenicity
~ Adverse events
~ Concomitant medication
~ ALM201 administration
~ PK profile
~ PK urine (12 hour collection)

May also include:

~ Biomarker/PD assessment – fresh biopsy (optional; up to 2 post-dose biopsies permitted at time of documented tumour response and/or at the point of disease progression)
~ Biomarker/PD assessment – blood (up to 12 post-dose samples permitted over 8 cycles and Final Study Visit)
~ Biomarker/PD assessment – ascites (to obtain aliquot a point of drain following normal clinical practise)

Cycle 1, Day 2

~ Adverse events
~ Concomitant medication
~ PK profile (end of profile sample)
~ Vital signs (pre-dose and approximately 1 hour after ALM201 administration)
~ ALM201 administration

May also include:

~ Biomarker/PD assessment – fresh biopsy (optional; up to 2 post-dose biopsies permitted at time of documented tumour response and/or at the point of disease progression)
~ Biomarker/PD assessment – blood (up to 12 post-dose samples permitted over 8 cycles and Final Study Visit)
~ Biomarker/PD assessment – ascites (to obtain aliquot a point of drain following normal clinical practise)

Cycle 1, Day 3-5

~ Adverse events
~ Concomitant medication

- Vital signs (pre-dose and approximately 1 hour after ALM201 administration)
- ALM201 administration
- PK profile (Day 3 only)

May also include:

- Biomarker/PD assessment – fresh biopsy (optional; up to 2 post-dose biopsies permitted at time of documented tumour response and/or at the point of disease progression)
- Biomarker/PD assessment – blood (up to 12 post-dose samples permitted over 8 cycles and Final Study Visit)
- Biomarker/PD assessment – ascites (to obtain aliquot a point of drain following normal clinical practise)

Cycle 1, Day 8

- Symptom-directed physical examination
- Vital signs (pre-dose and approximately 1 hour after ALM201 administration)
- Clinical chemistry
- Haematology
- Coagulation
- Adverse events
- Concomitant medications
- ALM201 administration

May also include:

- Biomarker/PD assessment – fresh biopsy (optional; up to 2 post-dose biopsies permitted at time of documented tumour response and/or at the point of disease progression)
- Biomarker/PD assessment – blood (up to 12 post-dose samples permitted over 8 cycles and Final Study Visit)
- Biomarker/PD assessment – ascites (to obtain aliquot a point of drain following normal clinical practise)

Cycle 1, Day 9-12

- Adverse events
- Concomitant medication
- Vital signs (pre-dose and approximately 1 hour after ALM201 administration)
- ALM201 administration

May also include:

- Biomarker/PD assessment – fresh biopsy (optional; up to 2 post-dose biopsies permitted at time of documented tumour response and/or at the point of disease progression)
- Biomarker/PD assessment – blood (up to 12 post-dose samples permitted over 8 cycles and Final Study Visit)
- Biomarker/PD assessment – ascites (to obtain aliquot a point of drain following normal clinical practise)

Cycle 1, Day 15

- Symptom-directed physical examination
- Vital signs (pre-dose and approximately 1 hour after ALM201 administration)
- Clinical chemistry
- Haematology
- Coagulation
- Adverse events
- Concomitant medications
- ALM201 administration

May also include:
- Biomarker/PD assessment – fresh biopsy (optional; up to 2 post-dose biopsies permitted at time of documented tumour response and/or at the point of disease progression)
- Biomarker/PD assessment – blood (up to 12 post-dose samples permitted over 8 cycles and Final Study Visit)
- Biomarker/PD assessment – ascites (to obtain aliquot a point of drain following normal clinical practise)

Cycle 1, Day 16-19

- Adverse events
- Concomitant medication
- Vital signs (pre-dose and approximately 1 hour after ALM201 administration)
- ALM201 administration
- PK profile (Day 18 only)

May also include:
- Biomarker/PD assessment – fresh biopsy (optional; up to 2 post-dose biopsies permitted at time of documented tumour response and/or at the point of disease progression)
- Biomarker/PD assessment – blood (up to 12 post-dose samples permitted over 8 cycles and Final Study Visit)
- Biomarker/PD assessment – ascites (to obtain aliquot a point of drain following normal clinical practise)

Cycle 1, Day 22/Cycle 2, Day 1 (and Cycles 3 & 4, Day 1)

- ECOG PS
- Full physical examination (including weight)
- Vital signs (pre-dose and approximately 1 hour after ALM201 administration)
- ECG (pre-dose only)
- Clinical chemistry
- Haematology
- Coagulation
- Urinalysis (include urine pregnancy test if applicable)
- Tumour assessment (serum marker as appropriate for tumour type)
- Immunogenicity
- Adverse events
- Concomitant medication

- PK sample (pre-dose)
- ALM201 administration

May also include:

- Biomarker/PD assessment – fresh biopsy (optional; up to 2 post-dose biopsies permitted at time of documented tumour response and/or at the point of disease progression)
- Biomarker/PD assessment – blood (up to 12 post-dose samples permitted over 8 cycles and Final Study Visit)
- Biomarker/PD assessment – ascites (to obtain aliquot a point of drain following normal clinical practise)

Cycles 2-4, Days 2-5

- ALM201 administration

Cycles 2-4, Day 8

- Symptom-directed physical examination
- Vital signs (pre-dose and approximately 1 hour after ALM201 administration)
- Clinical chemistry
- Haematology
- Coagulation
- Adverse events
- Concomitant medications
- ALM201 administration

May also include:

- Biomarker/PD assessment – fresh biopsy (optional; up to 2 post-dose biopsies permitted at time of documented tumour response and/or at the point of disease progression)
- Biomarker/PD assessment – blood (up to 12 post-dose samples permitted over 8 cycles and Final Study Visit)
- Biomarker/PD assessment – ascites (to obtain aliquot a point of drain following normal clinical practise)

Cycles 2-4, Days 9-12

- ALM201 administration

Cycles 2-4, Day 15

- Symptom-directed physical examination
- Vital signs (pre-dose and approximately 1 hour after ALM201 administration)
- Clinical chemistry
- Haematology
- Coagulation
- Adverse events
- Concomitant medications
- ALM201 administration

May also include:

- ~ Biomarker/PD assessment – fresh biopsy (optional; up to 2 post-dose biopsies permitted at time of documented tumour response and/or at the point of disease progression)
- ~ Biomarker/PD assessment – blood (up to 12 post-dose samples permitted over 8 cycles and Final Study Visit)
- ~ Biomarker/PD assessment – ascites (to obtain aliquot a point of drain following normal clinical practise)

Cycles 2-4, Days 16-19

- ~ ALM201 administration
- ~ PK profile (Cycles 2 & 4, Day 18 only)

Cycles 2-4, Day 22 (Day 1 of next cycle)

- ~ ECOG PS
- ~ Full physical examination (including weight)
- ~ Vital signs (pre-dose and approximately 1 hour after ALM201 administration)
- ~ ECG (resting 12-lead) (pre-dose only)
- ~ Clinical chemistry
- ~ Haematology
- ~ Coagulation
- ~ Urinalysis
- ~ Tumour assessment (including serum tumour markers and imaging assessment at the end of Cycles 2, 4, 6 and 8 only, unless clinically indicated)
- ~ Immunogenicity (pre-dose)
- ~ Adverse events
- ~ Concomitant medication
- ~ PK sample (pre-dose)
- ~ ALM201 administration

May also include:

- ~ Biomarker/PD assessment – fresh biopsy (optional; up to 2 post-dose biopsies permitted at time of documented tumour response and/or at the point of disease progression)
- ~ Biomarker/PD assessment – blood (up to 12 post-dose samples permitted over 8 cycles and Final Study Visit)
- ~ Biomarker/PD assessment – ascites (to obtain aliquot a point of drain following normal clinical practise)

Cycles 5-8, Day 1

- ~ ECOG PS
- ~ Full physical examination (including weight)
- ~ Vital signs (pre-dose and approximately 1 hour after ALM201 administration)
- ~ ECG (resting 12-lead) (pre-dose only)
- ~ Clinical chemistry
- ~ Haematology
- ~ Coagulation

- Urinalysis
- Tumour assessment (including serum tumour markers and imaging assessment at the end of Cycles 2, 4, 6 and 8 only, unless clinically indicated)
- Immunogenicity
- Adverse events
- Concomitant medication
- PK sample (pre-dose)
- ALM201 administration

May also include:

- Biomarker/PD assessment – fresh biopsy (optional; up to 2 post-dose biopsies permitted at time of documented tumour response and/or at the point of disease progression)
- Biomarker/PD assessment – blood (up to 12 post-dose samples permitted over 8 cycles and Final Study Visit)
- Biomarker/PD assessment – ascites (to obtain aliquot a point of drain following normal clinical practise)

<u>Cycles 5-8, Days 2-5, 8-12 & 15-19</u>

- ALM201 administration
- PK profile (Cycles 6 & 8, Day 18 only)

<u>Cycles 5-8, Day 22 (Day 1 of next cycle)</u>

- ECOG PS
- Full physical examination (including weight)
- Vital signs (pre-dose and approximately 1 hour after ALM201 administration)
- ECG (resting 12-lead) (pre-dose only)
- Clinical chemistry
- Haematology
- Coagulation
- Urinalysis
- Tumour assessment (including serum tumour markers and imaging assessment at the end of Cycles 2, 4, 6 and 8 only, unless clinically indicated)
- Immunogenicity (pre-dose)
- Adverse events
- Concomitant medication
- PK sample (pre-dose; Cycles 5-7 only)
- ALM201 administration

May also include:

- Biomarker/PD assessment – fresh biopsy (optional; up to 2 post-dose biopsies permitted at time of documented tumour response and/or at the point of disease progression)
- Biomarker/PD assessment – blood (up to 12 post-dose samples permitted over 8 cycles and Final Study Visit)
- Biomarker/PD assessment – ascites (to obtain aliquot a point of drain following normal clinical practise)

Final Study Visit

- ~ ECOG PS
- ~ Symptom-directed physical examination (including weight)
- ~ Vital signs
- ~ Clinical chemistry
- ~ Urinalysis
- ~ Haematology
- ~ Tumour assessment (if required e.g. to confirm response)
- ~ Immunogenicity
- ~ Adverse events
- ~ Concomitant medication

May also include:

- ~ Biomarker/PD assessment – fresh biopsy (optional; up to 2 post-dose biopsies permitted at time of documented tumour response and/or at the point of disease progression)
- ~ Biomarker/PD assessment – blood (up to 12 post-dose samples permitted over 8 cycles and Final Study Visit)
- ~ Biomarker/PD assessment – ascites (to obtain aliquot a point of drain following normal clinical practise)

5.2 Schedule of Study Assessments (ii)

Where the CRC recommend an adjustment to the dose schedule, the following assessments will be performed as standard. Please also refer to the Schedule of Study Assessments - Table (ii) and associated footnotes.

Cycle 1, Screening (up to 28 days prior to Cycle 1, Day 1)

- ~ Informed consent (optional consent for fresh biopsy samples for biomarker/PD assessments and blood sample for biomarker germ-line DNA testing)
- ~ Demographics
- ~ Medical history
- ~ Concomitant medication
- ~ Inclusion/exclusion checks
- ~ ECOG Performance Status
- ~ Full physical examination (including height and weight)
- ~ Vital signs
- ~ ECG
- ~ Clinical Chemistry
- ~ Haematology
- ~ Coagulation
- ~ Urinalysis
- ~ Serum pregnancy test (if applicable)
- ~ Tumour assessment
- ~ Biomarker/PD assessment – fresh biopsy (optional)
- ~ Biomarker/PD assessment – blood

- ~ Biomarker/PD assessment – ascites (if applicable in Part 2)
- ~ Biomarker testing – blood sample to obtain germ-line DNA

<u>Cycle 1, Day 1</u>

- ~ Full physical examination
- ~ Vital signs (pre-dose and approximately 1 hour after ALM201 administration)
- ~ ECG (pre-dose and approximately 30 minutes after ALM201 administration)
- ~ Clinical chemistry
- ~ Haematology
- ~ Coagulation
- ~ Urinalysis (include urine pregnancy test if applicable; no need to retest if within 7 days of last evaluation)
- ~ Tumour assessment (serum marker is appropriate for tumour type)
- ~ Adverse events
- ~ Concomitant medication
- ~ ALM201 administration
- ~ PK profile

May also include:

- ~ Biomarker/PD assessment – fresh biopsy (optional; up to 2 post-dose biopsies permitted at time of documented tumour response and/or at the point of disease progression)
- ~ Biomarker/PD assessment – blood (up to 12 post-dose samples permitted over 8 cycles and Final Study Visit)
- ~ Biomarker/PD assessment – ascites (to obtain aliquot a point of drain following normal clinical practise)

<u>Cycle 1, Day 2</u>

- ~ Adverse events
- ~ Concomitant medication
- ~ PK profile (end of profile samples)
- ~ ALM201 administration

May also include:

- ~ Biomarker/PD assessment – fresh biopsy (optional; up to 2 post-dose biopsies permitted at time of documented tumour response and/or at the point of disease progression)
- ~ Biomarker/PD assessment – blood (up to 12 post-dose samples permitted over 8 cycles and Final Study Visit)
- ~ Biomarker/PD assessment – ascites (to obtain aliquot a point of drain following normal clinical practise)

<u>Cycle 1, interim weekly assessments</u>

- ~ Symptom-directed physical examination
- ~ Vital signs (pre-dose and approximately 1 hour after ALM201 administration, where applicable)

~ Clinical chemistry
~ Haematology
~ Coagulation
~ Adverse events
~ Concomitant medications
~ ALM201 administration

May also include:

~ Biomarker/PD assessment – fresh biopsy (optional; up to 2 post-dose biopsies permitted at time of documented tumour response and/or at the point of disease progression)
~ Biomarker/PD assessment – blood (up to 12 post-dose samples permitted over 8 cycles and Final Study Visit)
~ Biomarker/PD assessment – ascites (to obtain aliquot a point of drain following normal clinical practise)

Cycle 1: other dosing days

~ Adverse events
~ Concomitant medication
~ Vital signs (pre-dose and approximately 1 hour after ALM201 administration)
~ ALM201 administration
~ PK profile (Day 3 & 18 only)

May also include:

~ Biomarker/PD assessment – fresh biopsy (optional; up to 2 post-dose biopsies permitted at time of documented tumour response and/or at the point of disease progression)
~ Biomarker/PD assessment – blood (up to 12 post-dose samples permitted over 8 cycles and Final Study Visit)
~ Biomarker/PD assessment – ascites (to obtain aliquot a point of drain following normal clinical practise)

End of Cycle 1 (Day 1 of Cycle 2)

~ ECOG PS
~ Full physical examination (including weight)
~ Vital signs (pre-dose and approximately 1 hour after ALM201 administration)
~ ECG (pre-dose only)
~ Clinical chemistry
~ Haematology
~ Coagulation
~ Urinalysis (include urine pregnancy test if applicable)
~ Tumour assessment (including imaging assessment at the end of Cycles 2, 4, 6 and 8 only, unless clinically indicated)
~ Immunogenicity
~ Adverse events
~ Concomitant medication
~ PK sample (pre-dose)

- ALM201 administration

May also include:

- Biomarker/PD assessment – fresh biopsy (optional; up to 2 post-dose biopsies permitted at time of documented tumour response and/or at the point of disease progression)
- Biomarker/PD assessment – blood (up to 12 post-dose samples permitted over 8 cycles and Final Study Visit)
- Biomarker/PD assessment – ascites (to obtain aliquot a point of drain following normal clinical practise)

<u>Cycles 2-4, interim weekly assessments</u>

- Symptom-directed physical examination
- Vital signs (pre-dose and approximately 1 hour after ALM201 administration, where applicable)
- Clinical chemistry
- Haematology
- Coagulation
- Adverse events
- Concomitant medications
- ALM201 administration

May also include:

- Biomarker/PD assessment – fresh biopsy (optional; up to 2 post-dose biopsies permitted at time of documented tumour response and/or at the point of disease progression)
- Biomarker/PD assessment – blood (up to 12 post-dose samples permitted over 8 cycles and Final Study Visit)
- Biomarker/PD assessment – ascites (to obtain aliquot a point of drain following normal clinical practise)

<u>Cycle 2-4: other dosing days</u>

- ALM201 administration
- PK profile (Cycles 2 & 4, Day 18 only)

<u>End of Cycles 2-4 (Day 1 of next cycle)</u>

- ECOG PS
- Full physical examination (including weight)
- Vital signs (pre-dose and approximately 1 hour after ALM201 administration, where applicable)
- ECG (resting 12-lead) (pre-dose only, where applicable)
- Clinical chemistry
- Haematology
- Coagulation
- Urinalysis
- Tumour assessment (including serum tumour markers and imaging assessment at the end of Cycles 2, 4, 6 and 8 only, unless clinically indicated)

- ~ Immunogenicity
- ~ Adverse events
- ~ Concomitant medication
- ~ PK sample (pre-dose)
- ~ ALM201 administration

May also include:

- ~ Biomarker/PD assessment – fresh biopsy (optional; up to 2 post-dose biopsies permitted at time of documented tumour response and/or at the point of disease progression)
- ~ Biomarker/PD assessment – blood (up to 12 post-dose samples permitted over 8 cycles and Final Study Visit)
- ~ Biomarker/PD assessment – ascites (to obtain aliquot a point of drain following normal clinical practise)

Cycles 5-8, Day 1

- ~ ECOG PS
- ~ Full physical examination (including weight)
- ~ Vital signs (pre-dose and approximately 1 hour after ALM201 administration)
- ~ ECG (resting 12-lead) (pre-dose only)
- ~ Clinical chemistry
- ~ Haematology
- ~ Coagulation
- ~ Urinalysis
- ~ Tumour assessment (including serum tumour markers and imaging assessment at the end of Cycles 2, 4, 6 and 8 only, unless clinically indicated)
- ~ Immunogenicity
- ~ Adverse events
- ~ Concomitant medication
- ~ PK sample (pre-dose)
- ~ ALM201 administration

May also include:

- ~ Biomarker/PD assessment – fresh biopsy (optional; up to 2 post-dose biopsies permitted at time of documented tumour response and/or at the point of disease progression)
- ~ Biomarker/PD assessment – blood (up to 12 post-dose samples permitted over 8 cycles and Final Study Visit)
- ~ Biomarker/PD assessment – ascites (to obtain aliquot a point of drain following normal clinical practise)

Cycles 5-8, interim weekly assessments (by telephone)

- ~ Adverse events
- ~ Concomitant medication

Cycles 5-8, dosing days

- ~ ALM201 administration

- ~ PK profile (Cycles 6 & 8, Day 18 only)

<u>End of Cycles 5-8 (Day 1 of next cycle)</u>

- ~ ECOG PS
- ~ Full physical examination (including weight)
- ~ Vital signs (pre-dose and approximately 1 hour after ALM201 administration, where applicable)
- ~ ECG (resting 12-lead) (pre-dose only, where applicable)
- ~ Clinical chemistry
- ~ Haematology
- ~ Coagulation
- ~ Urinalysis
- ~ Tumour assessment (including serum tumour markers and imaging assessment at the end of Cycles 2, 4, 6 and 8 only, unless clinically indicated)
- ~ Immunogenicity
- ~ Adverse events
- ~ Concomitant medication
- ~ PK sample (pre-dose; Cycles 5-7 only)
- ~ ALM201 administration

May also include:

- ~ Biomarker/PD assessment – fresh biopsy (optional; up to 2 post-dose biopsies permitted at time of documented tumour response and/or at the point of disease progression)
- ~ Biomarker/PD assessment – blood (up to 12 post-dose samples permitted over 8 cycles and Final Study Visit)
- ~ Biomarker/PD assessment – ascites (to obtain aliquot a point of drain following normal clinical practise)

<u>Final Study Visit</u>

- ~ ECOG PS
- ~ Symptom-directed physical examination (including weight)
- ~ Vital signs
- ~ Clinical chemistry
- ~ Urinalysis
- ~ Haematology
- ~ Tumour assessment (if required e.g. to confirm response)
- ~ Adverse events
- ~ Concomitant medication

May also include:

- ~ Biomarker/PD assessment – fresh biopsy (optional; up to 2 post-dose biopsies permitted at time of documented tumour response and/or at the point of disease progression)
- ~ Biomarker/PD assessment – blood (up to 12 post-dose samples permitted over 8 cycles and Final Study Visit)

- Biomarker/PD assessment – ascites (to obtain aliquot a point of drain following normal clinical practise)

5.3 Schedule of Study Assessments (iii)

Below is a summary of study assessments described by visit for Cycle 9 onwards. Please also refer to the Schedule of Study Assessments - Table (iii) and their associated footnotes.

Cycle 9 onwards, Day 1

- Symptom-directed physical examination
- Vital signs (pre-dose and approximately 1 hour after ALM201 administration)
- ECG
- Clinical chemistry
- Haematology
- Coagulation
- Urinalysis (include urine pregnancy test if applicable; no need to retest if within 7 days of last evaluation)
- Tumour assessment (serum marker is appropriate for tumour type)
- Immunogenicity
- Adverse events
- Concomitant medication
- ALM201 administration

May also include:

- Biomarker/PD assessment – fresh biopsy (optional; may take biopsy at the point of disease progression)

Cycle 9 onwards, interim weekly assessments (by telephone)

- Adverse events
- Concomitant medication

Cycle 9 onwards, dosing days

- ALM201 administration

End of Cycle 9 onwards

- ECOG PS
- Full physical examination (including weight)
- Vital signs (pre-dose and approximately 1 hour after ALM201 administration, where applicable)
- ECG (resting 12-lead) (pre-dose only, where applicable)
- Clinical chemistry
- Haematology
- Coagulation
- Urinalysis

- ~ Tumour assessment (imaging assessment at the end of every 4 cycles, unless clinically indicated)
- ~ Immunogenicity
- ~ Adverse events
- ~ Concomitant medication
- ~ ALM201 administration (where applicable)

May also include:

- ~ Biomarker/PD assessment – fresh biopsy (optional; may take biopsy at the point of disease progression)

<u>Final Study Visit</u>

- ~ ECOG PS
- ~ Symptom-directed physical examination (including weight)
- ~ Vital signs
- ~ Clinical chemistry
- ~ Urinalysis
- ~ Haematology
- ~ Tumour assessment (if required e.g. to confirm response)
- ~ Immunogenicity
- ~ Adverse events
- ~ Concomitant medication

May also include:

- ~ Biomarker/PD assessment – fresh biopsy (optional; may take biopsy at the point of disease progression)

5.4 Long-term Follow Up

Those patients who have not progressed by the Final Study Visit will be contacted every 2 months to check their disease status and commencement of next anti-cancer treatment.

5.5 Volume of Blood Sampling

Based on the unit blood volumes given for each study assessment, it is anticipated that patients enrolled to the dose escalation portion of the study will have a maximum of 548 mL of blood drawn if they complete all 8 cycles of treatment and the final study visit, and have the maximum number of blood samples taken for PK, PD and biomarker assessment.

<u>Table 7: Volume of Blood Sampling</u>

DURATION ON TRIAL	VOLUME OF BLOOD DRAWN
Screening, Cycles 1–8 & Final Study Visit	548 mL<br>(assumes max no. of PK and PD/biomarker samples taken)
**Notes:**	
PK - assuming all 61 samples are taken	Maximum of 122 mL
PD – assuming all 13 samples are taken	Maximum of 195 mL

Biomarker – sample for germ-line DNA	10 mL

The volume of blood drawn for each patient will also be described in the Patient Information Sheet. Efforts will be made to reduce the number of PK samples to be taken upon evaluation of cohort data during the trial. The number of blood samples for biomarker/PD assessment will be limited to the optimal sample timing required for the selected assays and will not exceed 13 samples over the total duration of the study, with no more than 2 samples taken on any study day, or 4 in any treatment cycle.

5.6 Description of Study Assessments

5.6.1 Adverse Events

AEs occurring from the time the patient gives consent but prior to administration of ALM201 will be captured on the Medical History CRF. Baseline medical conditions and AEs, other than the primary disease under evaluation, that worsen in severity or frequency during the study, should be recorded and reported as adverse events.

AEs will be graded according to the NCI CTCAE v4.03 for cancer clinical trials (Appendix B). For events not addressed in the CTCAE v4.03, the alternative severity classifications provided in Section 7.4.1 will apply.

AEs should continue to be captured for a further 30 +/-3 days from the end of the last injection of ALM201. On-going serious adverse events (SAEs) will be followed up until resolution.

Symptoms and signs of exacerbation or worsening of the patient's primary disease will be captured as AEs. For the purpose of data capture, disease progression as evaluated by the relevant response criteria, will not be considered to be an adverse event and will be captured in the Tumour Assessment CRF modules.

5.6.2 Laboratory safety measurements

Blood and urine samples for determination of clinical chemistry, haematology, coagulation and urinalysis parameters will be taken at the times given in the Schedule of Study Assessments and as clinically indicated. The date and time of collection will be recorded in the source data and on the CRF.

Clinical chemistry, haematology, coagulation analysis and urinalysis will be performed at each site's local laboratory or other local laboratories as appropriate. The approximate blood volumes for clinical chemistry and haematology (including coagulation) testing are 3.5 mL and 4 mL, respectively.

Copies of laboratory accreditation certificates and reference ranges will be provided prior to the analysis of the first patient sample.

The laboratory variables to be measured are described in Appendix C.

5.6.3 Resting 12-lead electrocardiogram (ECG)

For timing of individual measurements refer to Schedule of Study Assessments.

All 12-lead ECGs should be recorded while the patient is in the supine position. The Investigator or designated physician will review the paper copies of each of the timed 12-lead ECGs on each of the study days when they are collected.

ECGs will be recorded at 25 mm/sec. All efforts should be made to ensure that an identical ECG machine is used to collect traces for individual patients. If any clinically significant findings are observed on the ECG, the Investigator will record it as part of the medical history prior to start of dosing and as an adverse event post-dose, where the finding represent a clinically significant change from baseline.

5.6.4 Echocardiogram

An echocardiography assessment will be performed at Screening with follow up assessments as clinically indicated.

5.6.5 Vital signs

For the timing of individual measurements, refer to the Schedule of Study Assessments. The date and time of collection and measurement will be recorded on the appropriate CRF.

Measurements of heart rate, body temperature, blood pressure and respiratory rate will be made after the patient has been resting supine for a minimum of 5 minutes. For all administrations in hospital, the patients must wait for at least 60 minutes from the time of the ALM201 injection for observation and repeat vital signs.

5.6.6 Pharmacokinetic assessments (blood and urine)

A 12 hour urine collection will take place at Cycle 1, Day 1 for ALM201 urine PK analysis.

Blood samples will be drawn for the determination of ALM201 concentration-time profiles in plasma. Each patient will have up to 3 PK profiles drawn during Cycle 1, plus further profile sampling on Day 18 of Cycles 2, 4, 6 and 8. PK profile sampling will not exceed up to 12 samples each (with only the Cycle 1, Day 1 profile requiring sampling over a 2 day period). The maximum number of PK samples to be collected during any Cycle 1 dose schedule (up to Cycle 2, Day 1) will not exceed 40.

Nominal sampling time points for the PK profiles are dose dependent as follows:

Cycle 1, Day 1:

All doses (except 300mg): Predose, then 15 mins (+/- 5 mins), 45 mins (+/- 5 mins), 1.5 hr (+/- 10 mins), 2 hr (+/- 10 mins), 3 hr (+/- 10 mins), 4 hr (+/- 10 mins), 5 hr (+/- 10 mins), 6 hr (+/- 10 mins), 22 hr (+/- 1hr).

Doses of 300 mg: Predose, then 15 mins (+/- 5 mins), 45 mins (+/- 5 mins), 1.5 hr (+/- 10 mins), 2 hr (+/- 10 mins), 3 hr (+/- 10 mins), 4 hr (+/- 10 mins), 5 hr (+/- 10 mins), 6 hr (+/- 10 mins), 7 hr (+/- 10 mins), 8 hr (+/- 10 mins), 22 hr (+/- 1hr).

Cycle 1, Day 3 & 18:

All doses (except 300mg): Predose, then 30mins (+/- 5 mins), 60 mins (+/- 5 mins), 1.5 hr (+/- 10 mins), 2 hr (+/- 10 mins), 3.5 hr (+/- 10 mins), 5 hr (+/- 10 mins).

Doses of 300 mg: Predose, then 30 mins (+/- 5 mins), 60 mins (+/- 5 mins), 1.5 hr (+/- 10 mins), 2 hr (+/- 10 mins), 4.5 hr (+/- 10 mins), 7 hr (+/- 1 hr).

Cycles 2, 4, 6 & 8, Day 18:
All doses (except 300mg): Predose, then 30mins (+/- 5 mins), 60 mins (+/- 5 mins), 1.5 hr (+/- 10 mins), 2 hr (+/- 10 mins), 3.5 hr (+/- 10 mins), 5 hr (+/- 10 mins).

Doses of 300 mg: Predose, then 30 mins (+/- 5 mins), 60 mins (+/- 5 mins), 1.5 hr (+/- 10 mins), 2 hr (+/- 10 mins), 4.5 hr (+/- 10 mins), 7 hr (+/- 1 hr).

A single pre-dose sample will also be taken on Cycles 2-8 on Day 1.

	All doses (except 300 mg)				300 mg Doses			
	Cycle 1		Cycles 2 - 8	Cycles 2, 4, 6 & 8	Cycle 1		Cycles 2 - 8	Cycles 2, 4, 6 & 8
	D1	D3 & D18	Day 1	D18	D1	D3 & D18	Day 1	D18
Pre-dose	x	x	x	x	x	x	x	x
15 mins (+/- 5 mins)	x				x			
30 mins (+/- 5 mins)		x		x		x		x
45 mins (+/- 5 mins)	x				x			
60 mins (+/- 5 mins)		x		x		x		x
1.5 hrs (+/- 10 mins)	x	x		x	x	x		x
2 hrs(+/- 10 mins)	x	x		x	x	x		x
3 hrs(+/- 10 mins)	x				x			
3.5 hrs(+/- 10 mins)		x		x				
4 hrs (+/- 10 mins)	x				x			
4.5 hrs (+/- 10 mins)						x		x
5 hrs (+/- 10 mins)	x	x		x	x			
6 hrs (+/- 10 mins)	x			x	x			
7 hrs (+/- 10 mins)					x	x		x
8 hrs (+/- 10 mins)					x			
22 hrs (+/- 1 hr)	x			x	x			

Note that the ability to take the latter PK samples during the first day of a PK profile will be dependent on the local site operational procedures.

During the study the recommended sampling times may be adjusted following analysis of the data from previous cohorts. However, this is the maximum number of samples that will be taken for these assessments. Where the CRC recommend a revised dose schedule, the associated PK sampling schedule may also be adjusted as appropriate.

Nominal PK blood sampling times should be adhered to as closely as possible. It is essential that the actual time and date of collection of each blood sample be recorded in the patient's records and in the CRF.

The volume of blood to be collected per sample should be 2 mL to provide approximately 1 mL of plasma.

Note that where biopsy samples are collected for PD assessment (see Section 5.6.9.2), a specimen will be prepared and retained specifically for assessment of ALM201 in the tissue sample if required.

This measurement will be performed by a central laboratory. Full instructions for blood sample preparation, aliquoting of samples, sample storage and shipping details are provided in the Laboratory Manual for this study.

5.6.7 Tumour assessment

Tumour assessment will follow RECIST 1.1 (Eisenhauer, et al. 2009[i]) taking into account radiological assessment (CT or MRI) after every 2 cycles and any other informative tumour markers which may be assessed every cycle as appropriate.

For patients with prostate cancer, tumour assessment will follow the PCWG2 recommendations (Scher et al. 2008[xvii]) and will include:

- assessment of measurable and any selected non-measurable lesions by CT scan of chest abdomen and pelvis at Screening and on the completion of every 2 cycles; and

- assessment of PSA levels in serum at baseline and every 3 weeks (or at the end of each cycle).

Patients requiring assessment of bone lesions will have this performed as part of the CT or MRI assessment and will not require additional radiological bone scan assessment. Other clinically indicated assessments e.g. whole body MRI may be assessed as requested by investigator.

Note also for patients with prostate cancer, although CT scan is preferred radiographic measurement, MRI may be used in the event CT is not available. For each patient, radiographic measurement used at Screening (i.e., CT or MRI) must be used serially throughout the duration of study participation.

Additional scans may be performed to confirm a CR or PR or PD as per RECIST 1.1 (Eisenhauer, et al. 2009[i]), PCWG2, or other relevant guidelines. Patients on study after 8 cycles will have tumour assessment every 4 cycles i.e. every 12 weeks and at any additional time points as clinically indicated. Patients who do not have disease progression at the point of completing the study will be followed up every 8 weeks wherever possible to check their status e.g. by clinic visits or telephone contact.

All imaging procedures will be performed according to standard local imaging protocols to ensure consistency across study assessments.

5.6.8 Immunogenicity Assessment

Blood samples will be obtained on Day 1 of every cycle (pre-dose) for immunogenicity assessment. Approximately 4 mL of blood will be collected at each time point for preparation of serum samples.

Full instructions for sample preparation, handling procedures, aliquoting of samples, storage and shipping of these serum samples will be provided in the Laboratory Manual for this study.

5.6.9 Pharmacodynamic assessments

Biopsy, blood and ascites samples will be obtained to test for appropriate biomarkers and for biological activity of ALM201. Any biomarker/PD samples which remain untested may be stored for potential future analysis where a rationale for a specific biomarker assessment in that tumour type has been identified.

Full instructions for sample preparation, handling procedures, aliquoting of samples, storage and shipping for biopsy, blood or ascites samples will be provided in the Laboratory Manual for this study. In both parts of the study, any remaining samples obtained for biomarker/PD assessment will be retained for potential future analysis.

5.6.9.1. Archived biopsy

Patients with available archived tumour tissue will be asked to provide consent for access to some of this tissue (formalin-fixed paraffin-embedded (FFPE) sections) for evaluation of biomarker/PD parameters. In Part 2, where access to a tumour biopsy sample is required to confirm each patient's eligibility for the study (see Inclusion Criterion 2), the archived tumour biopsy material will be required to test for the angiogenesis signature during Screening where a fresh biopsy cannot be taken (Section 5.6.9.2).

5.6.9.2. Fresh biopsy

All patients will be asked to provide consent for access to archived tumour tissue where available. Where possible, fresh biopsies will be taken pre-dose and again during the study where the patient has a documented tumour response and/or at the point of disease progression i.e. up to 2 post-treatment biopsies. In Part 2, access to a tumour biopsy sample will be required to confirm each patient's eligibility for the study (see Inclusion Criterion 2). A fresh biopsy will be preferable for this purpose; however, where it is not possible to obtain this, confirmation of the angiogenesis signature may be performed on archived tumour tissue during Screening (Section 5.6.9.1).

Biomarker/PD testing in archival and/or fresh tumour biopsy material may include CD44, FKBPL, CD31, pFAK, pHer2, ITGA5, CA-125, ER, PR, and other relevant or exploratory biomarkers as appropriate for tumour type.

Biopsy samples taken during the trial may also have a sub-specimen prepared and stored for future evaluation of ALM201 in the tissue sample if required (Section 5.6.6).

5.6.9.3. Blood

Assessment of biomarker/PD activity in blood samples will be conducted in all patients, and will require taking a single Screening sample plus up to a further 12 samples for collection of plasma, PBMC or CTCs between Cycle 1-8 and the Final Study Visit; with no more than 2 samples taken on any study day, and 4 in any treatment cycle. Time-points may vary depending on method of analysis. It is essential that the actual time and date of collection of each sample be recorded in the patient's CRF.

One 15 mL blood sample will be taken for each time-point. It is intended that a plasma sample and PBMC or CTCs preparation be generated from each sample. Biomarker/PD assessments in blood may include assays for RASSF1 methylation, and other relevant or exploratory biomarkers appropriate for each tumour type.

5.6.9.4. Ascites

Assessment of PD activity in ascites will be conducted in relevant patients and will require taking a single Screening sample plus up to a further 5 samples may be conducted in relevant patients, and will involve taking up to up to 6 samples in total between Screening and over 8 cycles, with no more than one sample collection in 1 cycle. Actual time-points may within each cycle. It is essential that the actual time and date of collection of each sample be recorded in the patient's CRF.

The volume of ascites required will not be specified and an aliquot (or aliquots) will be prepared as collected. Biomarker/PD assessments in ascites may include stem cell count, and other relevant or exploratory biomarkers appropriate for ovarian cancer or other tumour types.

5.6.10 Germ-line DNA

A 10 mL blood sample will be taken at Screening (or alternative study time point) for collection of germ-line DNA in order to allow the comparison of germ-line and tumour cell DNA in this analysis.

6. STUDY MEDICATION AND ADMINISTRATION

6.1 Study Medication

The Quality Control Standards and requirements for ALM201 study medication are described in separate release protocols/Certificate of Analysis.

ALM201 is formulated as an aqueous solution containing 80mM sodium carbonate, 20mM Tris and 25mM sodium chloride, pH 6.5.

Table 8: Composition of ALM201 for Injection, 100 mg/mL

**Active component:**	One vial contains 100 mg ALM201
**Excipients:**	As listed above
**Vehicle:**	Aqueous solution of 80 mM sodium carbonate, 20 mM Tris, 25 mM sodium chloride
**Stability:**	Stability testing of ALM201 is on-going. Please refer to the IMP label for the Expiry Date associated with the current shelf-life of the product.
**Storage and handling:**	Stored in pharmacy at -20°C.<br>Once thawed and dispensed, ALM201 is stored at 2-8°C in a standard, domestic refrigerator.<br>Instructions for storage and handling will be provided in the Pharmacy Manual provided to sites.

6.2 Selection of Doses in the Study, Injection Duration and Duration of Treatment

The starting dose of ALM201 for the first cohort of patients will be 10 mg per dose given on Days 1-5, 8-12 and 15-19 of a 21-day treatment cycle (Section 1.5). In the absence of DLT, the dose of ALM201 will be escalated for future cohorts in recommended increments according to the protocol rules for dose escalation (Section 4.2).

Those patients who show an improvement or stable disease during treatment in the absence of DLT may receive up to 8 continuous cycles of therapy which is considered to be a complete treatment course for the purposes of this clinical trial. Patients may be permitted to receive further consecutive cycles of ALM201 where their cancer has not progressed on study and their investigator recommends this course of action, subject to availability of ALM201.

6.3 Packaging and Labelling

Clinical trial supplies will be provided as single-use glass vials of ALM201 for SC administration.

All vials and secondary packaging will be labelled for the purpose of the clinical trial in accordance with applicable regulatory requirements.

6.4 Preparation of ALM201 for Administration

Each vial of drug product contains 100 mg/mL ALM201. Where the dose level to be administered requires less than 1 mL in administration volume, the required volume will be diluted in saline to ensure that a constant volume of 1 mL is given in any single administration site. Instructions for the preparation and dilution of ALM201 for administration will be provided in the Pharmacy Manual given to sites.

6.5 ALM201 Administration

ALM201 administration will follow local guidelines for SC injections. The maximum volume for any single dose of ALM201 will not exceed 3.0 mL, with no more than 1.0 mL given in any single injection site.

The site for each SC injection may vary according to what is most comfortable for the patient, but one of the following sites is recommended:

- the lower abdomen, about an inch away from the belly button;
- the front of the thigh, about half way down and right in the middle;
- the back of the upper arm, into the fleshy tissue.

The site for each injection will be recorded in the CRF.

6.6 Storage in Pharmacy

The vials of drug product should be stored at -20°C. ALM201 must be stored in a safe and secure place with no access for unauthorized personnel whilst in Pharmacy.

6.7 Replacement of Medication Vials

Sufficient doses of medication will be supplied. In case the vial is broken or unusable, the vial should be replaced. Although the Sponsor need not be notified immediately in these cases, documentation of the use and/or loss of any vial must be recorded by the pharmacist on the medication accountability form.

6.8 Accountability

The Investigator is obliged to keep sufficient documentation of the delivery, use and destruction or return of unused, used or partially used IMP. The documentation must include dates, quantities, patient numbers, batch numbers or other identification number. The Investigator may assign some or all of the Investigator's duties for drug accountability to an appropriate pharmacist. Roles and responsibilities of site staff will be recorded in the Trial Master File (TMF).

All dispensing records both for on-site and home administration must be accurately maintained. The used vials will be counted as part of the drug accountability checks. The Investigator should maintain records that document adequately that the patients were administered or the doses specified in the protocol and reconcile all ALM201 IMP received for the trial. The local study monitor will be responsible for checking the drug accountability records maintained by the site during the monitoring visits.

The medication provided for this study is for use only as directed in the protocol. It is the Investigator/Institution's responsibility to establish a system for handling study drug so as to ensure that:

- deliveries of ALM201 are correctly received by a responsible person;
- such deliveries are recorded;
- study treatments are handled and stored safely and properly as stated on the label;
- study drug is only dispensed to study patients in accordance with the protocol; and
- any unused study drug is destroyed locally or returned for destruction in liaison with the study monitor.

Throughout the study, it must be possible to reconcile delivery records with records of usage and any destroyed/returned stock. Records of usage should include the identification of the patient to whom the study treatment was dispensed and the quantity and date of dispensing. This record is in addition to any drug accountability information recorded on the CRF. Any discrepancies must be accounted for on the appropriate forms. Certificates of delivery and return must be signed by the responsible pharmacist, and copies retained in the Pharmacy File.

The return or destruction of unused drug will be conducted after written approval by the Sponsor, with appropriate documentation and drug accountability procedures completed following destruction.

6.9 Treatment Allocation

In order to ensure that the appropriate numbers of patients are enrolled to each cohort and that enrolment to the study is appropriately controlled; on identifying a potential study patient, the Investigator is required to complete a patient registration request form confirming patient eligibility and requesting a place on a study cohort. Each patient registration form will be acknowledged in writing and where appropriate, sites will also receive a confirmation of enrolment form confirming the enrolment of the patient and the cohort and dose that the patient is assigned to. Confirmation of enrolment will also include the earliest possible date for the Cycle 1, Day 1 dose for each patient following the assigned enrolment stagger for that cohort. Patients must not be enrolled until this confirmation is received. There will be regular communication with study sites during the trial, in addition to the cohort review meetings to ensure Investigators are aware of enrolment status on the trial and suitable times for patient enrolment.

6.10 Blinding and Procedures for Un-Blinding the Study

This is an open-label study and there are no blinding or un-blinding procedures.

6.11 Permitted and Restricted Concomitant Medications/Treatments

All prescription, non-prescription, or over-the-counter medications including herbal remedies, dietary and nutritional supplements and complementary and alternative therapies given to, or taken by the patient at study entry (including Screening) and during the study must be clearly documented on the CRF.

Any medication considered necessary for the patient's safety and well-being may be given at the discretion of the Investigator(s).

For treatment of DLT or any other clinically significant events, any available standard therapy may be used as required. In the case of anemia, transfusions with packed red blood cells (pRBC) can be administered.

Prohibited treatments are summarized below:

- other antineoplastic agents;
- concurrent radiation treatment will be permitted during this study for symptom control;
- other IMPs.

6.12 Pre-medication guidelines

Pre-medication guidelines may be introduced or recommended where clinically significant injection site reactions or other study-related events e.g. histamine release, are seen on study. These guidelines will be reviewed on an on-going basis by the CRC (Section 8.1). The CRC may also recommend adjustments to the administration procedure or schedule based adverse event data available during the study.

6.13 Supportive care guidelines

Local irritation at the injection site may be treated according to local treatment guidelines. The CRC may recommend additional supportive care procedures based on on-going evaluation of adverse events during the trial (Section 8.1).

7. ADVERSE EVENTS AND REPORTING REQUIREMENTS

7.1 Assessment of Safety

All patients who receive treatment with ALM201 will be considered evaluable for safety. All AEs will be collected and recorded from the time of the patient's first dose up to and including the 30 day follow up (Final Study Visit). If the Investigator detects a SAE in a study patient after the end of the period of observation and considers the event possibly related to prior study treatment or procedures, they should contact the sponsor to determine how the adverse event should be documented and reported. Events occurring between the time of consent and first dose of ALM201 should be considered medical history and documented accordingly.

7.2 Adverse Event Definition

An AE is defined as any new untoward medical occurrence or worsening of a pre-existing medical condition in a patient or clinical investigation subject administered a medicinal product and which does not necessarily have a causal relationship with this treatment. An AE can therefore be any unfavorable and unintended sign (including a clinically significant abnormal laboratory finding, for example), symptom, or disease temporally associated with the use of a medicinal (investigational) product, whether or not considered related to the IMP.

During clinical trials, adverse events can be spontaneously reported or elicited during open-ended questioning, examination, or evaluation of a patient. To prevent reporting bias, patients should not be questioned regarding the specific occurrence of one or more adverse events.

Adverse events include: (a) worsening (change in nature, severity, or frequency) of conditions present at the start of the study, (b) intercurrent illness, (c) drug interactions, (d) experiences related or possibly related to concomitant medications, (e) clinically significant abnormal laboratory values or shifts from baseline, and (f) clinically significant abnormalities in physical examination, vital signs, weight, or electrocardiogram.

Progression of the disease under study will not be captured as an AE.

Surgical procedures or other therapeutic interventions themselves are not AEs, but the condition for which the surgery/intervention is required is an AE and should be documented accordingly.

Planned surgical measures and the condition(s) leading to these measures are not AEs, if the condition(s) was (were) known before the period of observation and did not worsen during study. In the latter case the condition should be reported as medical history.

7.3 Importance of Adverse Event Reporting

Timely and complete reporting of safety information assists Almac, Investigators and the CRC in identifying any untoward medical occurrence, thereby allowing: (1) safety of study patients; (2) a greater understanding of the overall safety profile of the investigational drug; (3) recognition of dose-related investigational drug toxicity; (4) appropriate modification of study protocols; (5) improvements in study design or procedures; and (6) adherence to regulatory requirements.

7.4 Evaluating Adverse Events

All identified AEs must be recorded and described on the appropriate AE page of the CRF, except for those events occurring prior to the first dose of ALM201, which should be recorded on the Medical History CRF. If known, the diagnosis of the underlying illness or disorder should be recorded, rather than its individual symptoms. The following information should be captured for all AEs: date of onset and resolution, severity of the event (see definitions in Section 7.4.1), assessment whether the event was serious or non-serious (see definitions in Section 7.4.2), Investigator's opinion of the relationship to investigational drug (see definitions in Section 7.4.6), treatment required for the AE, action taken with IMP, and information regarding resolution/outcome.

7.4.1 Severity

All AEs (including SAEs) are to be accurately recorded on the AE page of the patient's CRF. Each event will be graded for severity using the classifications of CTCAE v4.03. For events not addressed in the CTCAE v4.03, classifications the following grading will apply:

- **Mild (Grade 1)** - Mild; asymptomatic or mild symptoms; clinical or diagnostic observations only; intervention not indicated.
- **Moderate (Grade 2)** - Moderate; minimal, local or noninvasive intervention indicated; limiting age-appropriate instrumental Activity of Daily Living (ADL).
- **Severe (Grade 3)** - Severe or medically significant but not immediately life-threatening; hospitalization or prolongation of hospitalization indicated; disabling; limiting self-care ADL.
- **Life-threatening (Grade 4)** - Life-threatening consequences; urgent intervention indicated.
- **Death (Grade 5)** related to AE.

7.4.2 Seriousness

A **serious adverse event** is any untoward medical occurrence that at any dose (including overdose):

- Results in death.
- Is life-threatening:
  - "Life-threatening" means that the subject was at immediate risk of death at the time of the serious adverse event; it does not refer to a serious adverse event that hypothetically might have caused death if it were more severe.
- Requires unplanned hospitalization or prolongation of existing hospitalization due to complication:

  - For example, adverse event requires hospital in-patient admission, or prolongation of hospital stay. Note that visits to a hospital by ambulance or to the emergency room without admission will not be regarded as hospitalization unless the event fulfills any other of the serious criteria.

- Results in persistent or significant disability or incapacity:
  - Persistent or significant disability or incapacity" means a permanent or significant and substantial disruption of a person's ability to carry out normal life functions.

- Is a congenital anomaly or birth defect.

- Is an important medical event:
  - Medical and scientific judgment should be exercised in deciding whether expedited reporting is appropriate in situations where none of the outcomes listed above occurred. Important medical events that may not be immediately life-threatening or result in death or hospitalization but may jeopardize the subject or may require intervention to prevent one of the other outcomes listed in the definition above should also usually be considered serious. Examples of such events include allergic bronchospasm requiring intensive treatment in an emergency room or at home, blood dyscrasias, or convulsions that do not result in inpatient hospitalization, or the development of drug dependency or drug abuse.

7.4.3 Pregnancy

In principle, pregnancy and the lactation period, or fathering a child, are exclusion criteria for clinical studies involving investigational drugs. In the event of a pregnancy occurring during the course of this study, Almac must be notified immediately. If the pregnancy involves a patient enrolled to the trial, the patient should be immediately withdrawn from study. The pregnant patient or the patient's partner should followed-up during the entire course of the pregnancy and postpartum period.

Parental and neonatal outcomes must be recorded even if they are completely normal and without AEs. Off-spring should be followed up for at least 8 weeks after delivery. Longer observation periods may be determined by the sponsor if an adverse outcome of the pregnancy was observed.

Pregnancies occurring during the study up to 90 days after final dose of IMP must also be reported to the Drug Safety Department (fax: +1 919.468.2288 or e-mail: dsafety@ockham.com) within one working day of becoming aware of them using a Clinical Trial Pregnancy Reporting Form. A pregnancy is not a SAE unless the outcome of the pregnancy meets serious criteria as defined in Section 7.4.2. When a "pregnancy is detected without an adverse outcome", the Investigator should complete the Pregnancy Form and send this to the sponsor's safety department as per the above. It should be clearly stated that no AE was observed. If the outcome of the pregnancy meets the criteria of a serious adverse event (i.e. congenital anomaly, stillbirth, neonatal death), then it should be reported as described in Section 7.6.

7.4.4 Misuse and Overdose

Drug misuse and drug overdose should be reported in the same format and within the same timelines as a serious adverse event, even if they may not result in an adverse outcome.

Overdose is defined as any dose administration where >10% over the correct dose amount is administered whether or not associated with an adverse event.

For monitoring purposes, any case of overdose must be reported on an overdose form.

When an “overdose” or “drug misuse” of the investigational product occurs without an adverse event the Investigator should complete the Overdose Form and send this to the sponsor’s safety department (fax: +1 919.468.2288 or e-mail: dsafety@ockham.com). It should be clearly stated that no adverse event was observed has occurred.

If the pharmacy discovers that an overdose has or may have been administered they should contact the Investigator and study coordinator.

7.4.5 Investigational Product Complaints

Pharmaceutical technical complaints associated with the investigational product must be reported to the sponsor immediately. The same reporting timelines as for serious adverse events apply.

7.4.6 Relationship

All AEs (including SAEs) will be assessed for the relationship of the adverse event to the study drug using the following definitions:

- **Not/Unlikely Related**
  The AE is not related if exposure to the investigational product has not occurred, *OR* the occurrence of the AE is not reasonably related in time, *OR* the AE is considered unlikely to be related to use of the investigational product because there are no facts (evidence) or arguments to suggest a causal relationship AND there is a possible alternative explanation.

- **Possibly Related**
  The administration of the investigational product and AE are considered reasonably related in time AND there are facts (evidence) or arguments to suggest a causal relationship. This does not exclude that the AE could be explained by causes other than exposure to the investigational product.*

- **Probably Related**
  Exposure to the investigational product and AE are reasonably related in time *AND* the investigational product is more likely than other causes to be responsible for the AE, *OR* is the most likely cause of the AE.

- **Definitely Related**

There is a reasonable temporal sequence between exposure to the investigational product and the AE, *OR* the event follows a known or expected response pattern to the investigational product; *AND* is confirmed by improvement on stopping/ reducing the dosage of the investigational product. It may also be confirmed by reappearance upon repeated exposure where this is medically and ethically acceptable.

The relationship of the study treatment to an AE will be determined by the Investigator and subsequently reviewed by the Medical Monitor.

For reporting and data analysis purposes, AEs reported with a causality assessment of "Definitely", "Probably" and "Possibly" are to be considered as "having a reasonable causal relationship" to study drug. In case of disagreement between the Investigator and the Sponsor's Medical Monitor the more conservative assessment will determine the reportability of the case.

*For consideration of DLTs and subsequent dose escalation decisions, the likely causality of clinically significant AEs as defined in Section 4.2.1 must be carefully considered.

7.5 Evaluating Dose Limiting Toxicities (DLTs)/Serious Adverse Events (SAEs)

DLTs, SAEs classified as DLTs and SAEs will be noted on the AE CRF and on a SAE form. This form will classify the event as a DLT only; SAE only; or DLT and SAE.

7.5.1 Unexpected adverse events

The Sponsor will assess all serious adverse events whether they are expected or unexpected. An unexpected adverse event is any adverse drug event, the outcome, specificity or severity of which is not consistent with those noted in the current IB.

7.6 Reporting DLTs/SAEs

Adverse events classified as DLTs and/or SAEs using the definitions above must be recorded on the Adverse Event CRF.

The Principal Investigator (or designee) will notify Drug Safety within 24 hours of identifying a DLT or SAE, whether related or unrelated to investigational drug. DLTs should be recorded on the AE CRF, denoting that they are being considered as DLTs. SAEs should be reported by completing the SAE form and e-mailing (or faxing if e-mail is not possible) this and any available supporting documentation as per the details below.

It is the responsibility of the Investigator or delegate to inform the Drug Safety department of all SAEs.

SAE FACSIMILE TRANSMISSION: +1.919.468.2288

SAE E-MAIL CONTACT: dsafety@ockham.com

MEDICAL MONITOR TELEPHONE CONTACT: +44(0)7791934182

7.6.1 Reporting SAEs to the IRB or Ethics Committee and Regulatory Authorities

The Investigator must comply with the applicable regulatory requirements related to the reporting of SAEs to the required Independent Ethics Committees (IEC). The CRO will provide Investigators with the final related unexpected SAE reports. The CRO will send the Principal Investigator a Council for International Organisations of Medical Sciences (CIOMS) form for them to report to their IEC. Other SAEs (i.e. expected or unrelated SAEs) should be reported per the relevant institution's procedures.

Until such time that an AE is included in the IB, it should be considered unexpected, regardless of whether the AE has been the subject of a previous Safety Update. All expectedness assessments will be made by the Sponsor or designee.

All events qualifying as Suspected Unexpected Serious Adverse Reactions (SUSARs) will be reported to the relevant regulatory authorities and Investigators by the CRO, who will in turn notify their IEC. SUSARs are required to be reported within 7 calendar days for life threatening events and those resulting in death, or 15 calendar days for all others. These timeframes begin with the first notification of the SUSAR to the CRO or Almac from the Investigator.

7.6.2 Follow-up information on an SAE

Collection of complete information concerning SAEs is extremely important. Thus, follow-up information which becomes available as the SAE evolves, as well as supporting documentation (e.g. hospital discharge summaries and autopsy reports), should be collected subsequently, if not available at the time of the initial report, and immediately sent using the same procedure as the initial SAE report. The original SAE form must be kept on file at the study site. The sponsor will also review SAE reports for missing information and send queries to the site for resolution as appropriate.

Appropriate diagnostic tests should be performed and therapeutic measures, if indicated, should be instituted. Appropriate consultation and follow-up evaluations should be carried out by the Principal Investigator (or designee). An SAE is followed until it is considered resolved; returns to baseline; is chronically ongoing or explained by the Principal Investigator.

8 DATA EVALUATION: CRITERIA FOR EVALUATION OF OBJECTIVES

8.1 Cohort Review Committee (CRC)

The CRC will comprise of the Principal Investigators (or their representatives), the Sponsor's Medical Monitor and a patient representative and invited experts (such as the pharmacokineticist). The Committee will meet by telephone (or in person if possible) to review safety, PK and PD (where available) from the individual cohorts prior to dose-escalation to a subsequent cohort and at other times as needed. Appropriate representatives of the Sponsor and the coordinating CRO will also attend and minute the meeting. Decisions of the CRC will be documented and sent to the sites.

The CRC will:

- review the safety data from the first cycle of each cohort during Part 1 of the study and make dose-escalation (or de-escalation) decisions. AEs occurring in Cycle 2 and beyond which meet the definition of a DLT or are considered clinically significant may also be considered in making dose escalation decisions;
- consider other AEs, or possible trends in AEs, during the dose escalation phase which may inform dose escalation;
- confirm study may progress to next defined 100% dose escalation step or justify the rationale for reduced escalation step based on available safety, PK and PD data;
- confirm the MTD, MFD and/or a BAD;
- review safety data for additional patients recruited in Part 2 (at least every 2 months);
- recommend alternative dose administrations schedules during the study conduct;
- advise on (and approve where appropriate) required pre-medication;
- advise on individual patient dose adjustments;
- advise on any adjustment to study assessment time points e.g. PK sampling, based on data available during the trial and protocolled limit on blood volumes and number of samples permissible in a given period.

8.2 End of Study and Study Completion

End of study is defined as the last patient visit or study assessment. Study completion occurs upon final database lock and production of the main study report. In the case where a patient is (or patients are) continuing to receive ALM201 after 8 cycles of treatment under this protocol, "End of Study" and "Study Completion" may be defined at a point when all patients have had the opportunity to complete 8 cycles of IMP for the purposes of locking the database and preparing the CSR. Additional data from the patient(s) continuing on treatment for up another 8 cycles will be presented as an Addendum to the CSR upon cessation of treatment. A further addendum will be generated to summarise long-term outcome follow up of patients following completion of study treatmentafter withdrawal from the study.

8.3 Statistical Considerations

Detailed statistical analysis information will be provided separately in the Statistical Analysis Plan (SAP). The SAP will detail all data handling rules, including the management of missing values and the handling of data for withdrawn patients. The SAP will also outline protocol violation criteria along with any specific analysis population definitions. Any deviations to the planned analyses specified or populations defined within the SAP will be justified in writing and presented within the final CSR.

The clinical database lock will occur after all data are reconciled (i.e. "cleaned") for all patients who participate in both Part 1 and Part 2 of the study. A single CSR will be generated for this study (Section 8.2). The SAP will be finalized and signed before the database lock.

In all tables, listings and figures the dose-escalating cohorts will be reported from the lowest to the highest dose. Where appropriate, safety parameters for those patients in Part 1 who received what becomes identified as the MTD, MFD or a BAD to be explored in Part 2, and those receiving the same dose in Part 2, may be combined and summarized, as well as being summarized separately. A clear distinction will be made in the tables to differentiate between the cohorts receiving MTD, MFD or BAD during Part 1, Part 2 or both parts combined. The SAP will describe all tables, listing and figures to be prepared, such as data summaries for patients who have/have not received prior anti-angiogenic therapy/ies in Part 2.

8.4 Demographic and Other Baseline Characteristics

Demographic characteristics will be listed and summarized, including any previous therapy details. Other baseline characteristics will only be listed.

8.5 Statistical Methods for Safety Parameters

All safety and tolerability assessments will be based on the safety analysis set, which is defined as all patients who have received at least one dose of study medication.

No formal statistical analysis will be performed on safety data.

Vital signs, resting 12-lead ECGs, clinical chemistry, haematology, coagulation, and urinalysis data will be listed by dose group and time-point.

The number and percent of patients experiencing one or more AEs will be summarized by dose level group, relationship to study drug, and severity. AEs will be coded using MedDRA terminology. AEs that have missing onset dates will be considered to be treatment-emergent, unless the stop date is known to be prior to the first administration of the study medication.

SAEs and DLTs will also be presented separately.

8.6 Statistical Methods for Pharmacokinetic Parameters

PK parameters will be estimated for each patient using a fully validated version of WinNonlin® Pro (Version 5.2.1 Pharsight Corporation, Mountain View, USA. 2008) or suitable alternative. The

following parameters will be derived, where appropriate, from the individual plasma concentration versus time profiles of ALM201.

Table 9: Definition of PK Parameters

PARAMETER	DEFINITION
C_{inf}	The observed concentration at the end of the administration.
C_{max}	The maximum observed concentration.
t_{max}	The time at which C_{max} was apparent.
AUC_{0-t}	The area under the concentration versus time curve from time zero to the sampling time at the last quantifiable concentration (C_t) at t_{last} (the time of the last quantifiable concentration) calculated by the linear trapezoidal rule.
λ_z	The apparent terminal rate constant, estimated using the negative slope of the least square regression analysis of the log concentration versus time data for the terminal linear portion of the curve.
$t_{½}$	The apparent terminal half-life, calculated from $Log_e 2 / \lambda_{z.}$
$AUC_{0-\infty}$	The area under the concentration-time curve estimated from time zero to infinity as the sum of the two areas: AUC_{0-t} and AUC_{extrap}, where AUC_{extrap} is calculated as $C_t / \lambda_{z.}$
CL	The systemic clearance calculated as: Dose/ $AUC_{0-\infty.}$
V_{ss}	The apparent volume of distribution at steady state calculated as: Dose/AUC x (AUMC/ $AUC_{0-\infty}$ - T/2) where T is the duration of intravenous injection.

Additional PK parameters may be calculated as appropriate.

8.7 Evaluation of Tumour Response

Tumour assessment will follow RECIST 1.1 (Eisenhauer, et al. 2009[i]) or other relevant guidelines e.g. PCWG2 recommendations for prostate cancer (Scher et al. 2008[xvii]). In Part 2, response rate for each enrichment cohort will be assessed and an exact 95% CI calculated.

8.8 Estimated Sample Size

It is anticipated that up to 84 patients with advanced cancer for whom a rationale for treatment with an anti-angiogenic agent exists may be enrolled in this study. The study will follow a dose escalation design, and the number of patients treated will therefore be dependent upon the number of dose levels investigated, the size of each cohort in Part 1, and the number of enrichment cohorts of interest in Part 2.

The study will commence with an accelerated dose escalation design (Simon, 1997[xv]), then switch to a conventional algorithm (3+3 patients per dose level) in order to identify the MTD, escalating on 0/3 or 1/6 DLTs, and de-escalating if 2 or more patients with DLTs are encountered. The MTD will

be the highest dose level at which 0 of 3 or 1 of 6 patients experience a DLT, with the next higher dose having at least 2 of 3 or 2 of 6 patients experiencing a DLT.

The following table shows the operating characteristics of this 3+3 design. Under this design, there is a 71% chance of escalation if the true but unknown rate of DLT is 20% and less than 50% chance of escalation if the true but unknown rate of DLT is higher than 30%.

Table 10: Operating characteristics of a 3+3 cohort design

True but Unknown Rate of DLT (%)	Probability of Escalation (%)
20	71
30	49
40	31
50	17
60	8

To ensure that the toxicity at the MTD, MFD or a selected BAD is acceptable in tumour types of interest, up to 3 enrichment cohorts of 12 patients will be treated at the selected dose in Part 2. The estimation of toxicity rates will be based on these patients. A 90% confidence interval for the rate of any toxicity among patients treated at the MTD, MFD or BAD will cover, at most, 50 percentage points.

9 QUALITY ASSURANCE

9.1 Data Recording

All CRF data will be collected using an electronic Case Report Form (eCRF) within a fully validated and CFR 21 Part 11 compliant Electronic Data Capture (EDC) system. All data will be entered into the CRF by the Site Staff. These data will then be source data verified and reviewed by the study monitor before data cleaning by Data Management is performed. All queries will be raised and resolved within the EDC system. During entry programmatic checking of the data will be performed and once saved into the database more complex programmatic checks will also be performed. During the conduct of the study all system users will have real time access to the data, the level of access to the data and study privileges will be determined by their user role.

After all queries have been resolved, the SAP approved and signed, and any summary/analysis populations approved, the database will be locked and the data released for summary and analysis. All summary and analysis of the data will be performed using SAS® version 9.1 or later and/or WinNonLin® Pro version 5.2.1 (or later version where appropriate).

9.2 Study Monitoring

The assigned study monitor will review the progress of the study on a regular basis to ensure adequate and accurate data collections. Monitoring site visits to review CRFs, patient case notes, administrative documentation including the Investigator Site File and frequent telephone/e-mail communications with site will be performed throughout the study.

At each study monitoring visit the Investigator will make available all records pertaining to the study. To allow sufficient time to assemble documentation for the study monitor, monitoring visits will be confirmed in advance of planned visits.

9.3 Clinical Study Audit

The Sponsor, Sponsor representative or external regulatory agency may at any time during or after completion of the study conduct a Good Clinical Practice (GCP) audit. Prior notice will be given to each site selected for audit in advance of a planned audit.

9.4 Clinical Study Report

The results of the study will be presented in an integrated CSR study report according to International Conference on Harmonisation of Technical Requirements for Medicinal Products for Human Use (ICH) guidelines.

9.5 Data Retention and Availability

The Investigator is required to maintain copies of all essential study documentation, including the Site Study File, a disc containing all CRF data (including the full audit trail and all data queries), signed informed consent forms, and records for the receipt and disposition of study medications, for a period of at least five years after study completion, as specified by ICH GCP and longer if required by local or regulatory authorities.

During the study, the Investigator must make study data accessible to the study monitors, the Sponsor (or a third party auditor assigned by the Sponsor), and relevant Investigational Review Board (IRB)/IECs and regulatory agencies. A file for each patient must be maintained that includes the signed informed consent form and all source documentation related to that patient. The Investigator must ensure the availability of source documents from which the information in the CRF was derived.

10 ETHICS REVIEW/INFORMED CONSENT

The final study protocol and patient informed consent form will be approved by the appropriate IEC for each investigational site. Approval will be received in writing before initiation of the study.

Changes to the protocol during the trial will be documented as amendments. Depending on the contents of the amendment and local legal requirements, the amendment will be submitted for approval to the relevant IECs and to the relevant competent authorities prior to implementation. Exceptions are cases of changes made to protect patient safety, which will be implemented immediately.

If an amendment substantially alters the trial design, increases the potential risk to the patients, affects the treatment of the patient or might otherwise influence the willingness of the patient to participate in the trial, then the information sheet must be revised and submitted to the relevant IEC and, where necessary, to the relevant competent authorities, for review and approval. When a patient is currently undergoing trial procedures and is affected by the amendment, then the patient must be asked to consent again using the new information sheet.

10.1 Ethical Conduct of the Study

The study will be conducted in accordance with ICH GCP, the Declaration of Helsinki, the European Union (EU) Clinical Trials Directive 2001/20/EC, the GCP Directive 2005/28/EC, and the requirements of local IRB/IECs.

10.2 Informed Consent

The principles of informed consent in the Declaration of Helsinki and GCP Guidelines will be implemented before any protocol-specific procedures or interventions are carried out.

All patients will be informed that participation is voluntary and that they can cease participation at any time without necessarily giving a reason and without any penalty or loss of benefits to which they are entitled.

With the help of the information sheet the patient will be informed about the IMP and anticipated effects, and the reason, design and implication of the trial. The patient must give consent to participate prior to enrolment in the trial. This consent must be given in writing. The Investigator who conducts the informed consent discussion must also sign. The Investigator may delegate this responsibility to a suitably qualified member of the study team e.g. sub-investigator, if permitted by local regulations. This delegation of responsibility must be recorded in the study file. By giving signed consent, the patient will confirm that their participation is voluntary and that they will follow the instructions of the Investigator and answer the questions asked. Signatures must be personally dated.

The signed and dated consent form will be kept by the Investigator. Prior to participation in the trial, the patient should receive a copy of the signed and dated written informed consent form.

The consent form and information sheet must include all elements required by law, local regulations, GCP and ICH guidelines including consent to allow the Sponsor, Sponsor representative or external

regulatory auditor to review the patient's medical records. This gives permission to examine, analyze, verify and reproduce any records and reports that are important to the evaluation of the trial.

Any party with direct access must take all reasonable precautions within the constraints of the applicable regulatory requirement(s) to maintain the confidentiality of the patient's identities and Sponsor's proprietary information. It is the study monitor's responsibility to verify that each patient has consented, in writing, to direct access.

10.3 Patient participation card

A study participation card will be provided to each patient on the trial. The card will indicate that they are participating in a clinical trial, and give the name and contact details of the sponsor and the Investigator/study site. This will include the local 'helpline' number for patients, their carers or other health care professionals to contact at any time of day or night. The patient will be asked to retain this card while they are participating in the trial and show it to any other medical practitioners they consult during this time. They will be advised to contact the Investigator/study site if there are any questions.

10.4 Insurance

Insurance for the patients participating in this trial will be arranged by Almac, as Sponsor of the clinical trial, in accordance with the regulatory requirements of the countries involved. A copy of the country-specific insurance certificates will be held in the TMF and in the Investigator site file.

11 PUBLICATION POLICY

The original CRFs and all data generated during the clinical study using the given protocol will become the property of the Sponsor.

Any proposed publication or presentation (including a manuscript, abstract or poster) for submission to a journal or scientific meeting should be sent to the Sponsor for review at least one month prior to submission. No single centre or groups of centres may publish individually. Publications arising from this clinical study will include as a minimum all Investigators and Sponsor representatives as authors. The Sponsor's comments on the proposed publication shall be considered in good faith by the authors. The Sponsor may delay such submission by a maximum of ninety (90) days if it reasonably believes that publication of results may compromise its intellectual property rights, or else require that such information or data are removed from the proposed publication. Publication of the results will not include confidential information without the permission of the Sponsor. Approval of such requests to submit for publication will not be unreasonably withheld.

The Sponsor may announce quality assured summary data in order to comply with Financial Regulatory Authorities, whilst ensuring, so far as possible, that such announcements will not compromise the Investigators ability to publish the data in appropriate scientific forums.

APPENDIX A: ECOG PERFORMANCE STATUS

Eastern Co-operative Oncology Group (ECOG) Performance Status

Grade	ECOG
0	Fully active, able to carry on all pre-disease performance without restriction.
1	Restricted in physically strenuous activity but ambulatory and able to carry out work of a light or sedentary nature, e.g. light house work, office work.
2	Ambulatory and capable of all self-care but unable to carry out any work activities. Up and about more than 50% of waking hours.
3	Capable of only limited self-care, confined to bed or chair more than 50% of waking hours.
4	Completely disabled. Cannot carry on any self-care. Totally confined to bed or chair.
5	Dead.

Oken MM et al, 1982[xviii]

APPENDIX B: NCI CTCAE v4.03

A complete copy of the NCI CTCAE v4.03 will be held in each Site Study File.

Please use link below to access most current version of the NCI CTCAE.
http://ctep.cancer.gov/protocolDevelopment/electronic_applications/ctc.htm#ctc_40 .

APPENDIX C: LOCAL LABORATORY PARAMETERS

Clinical chemistry	*Haematology, including coagulation screen*
Calcium	Red cell count
Total protein	Hemoglobin
Albumin	Hematocrit
Total bilirubin	Absolute reticulocyte count
Alanine transaminase (ALT, SGPT)	Platelet count
Aspartate transaminase (AST, SGOT)	White blood cells
Alkaline phosphatase	Leucocyte differential count (% & absolute)
Glucose (random)	International normalized ratio or prothrombin time
Sodium	Activated partial thromboplastin time
Potassium	
Bicarbonate	
Chloride	
Magnesium	
Urea	
Creatinine	
Phosphate	
Uric acid	
Pregnancy test as required (Screening)	

Urinalysis
Glucose
Protein
Bilirubin
Ketones
Blood
pH
Specific gravity &<br>Microscopic examination when indicated
Pregnancy test as required

APPENDIX D: Cockcroft-Gault Formula

Creatinine clearance should be calculated using the Cockroft-Gault Formula, which is given below:

$$\textbf{Males:} \quad \frac{1.25 \times (140 - \text{age}) \times \text{weight (kg)}}{\text{Serum creatinine } (\mu\text{mol/l})}$$

$$\textbf{Females:} \quad \frac{1.05 \times (140 - \text{age}) \times \text{weight (kg)}}{\text{Serum creatinine } (\mu\text{mol/l})}$$

Cockroft DGM, 1976[xix]

REFERENCES

[i] Eisenhauer EA, Therasse P, Bogaerts J, et al. (2009) New response evaluation criteria in solid tumours: Revised RECIST guideline (version 1.1). European Journal of Cancer 228-247. (http://www.eortc.be/recist/documents/RECISTGuidelines.pdf)

[ii] Rustin GJS, Vergote I, Eisenhauer E, et al. (2011) Definitions for Response and Progression in Ovarian Cancer Clinical Trials Incorporating RECIST 1.1 and CA 125 Agreed by the Gynecological Cancer Intergroup (GCIG). Int J Gynecol Cancer 21: 419-423

[iii] Valentine A, O'Rourke M, Yakkundi A, et al. (2011) FKBPL and peptide derivatives: novel biological agents that inhibit angiogenesis by a CD44-dependent mechanism. Clin Cancer Res 17 (5): 1044-56.

[iv] Folkman J. (2007) Angiogenesis: an organizing principle for drug discovery? Nature Reviews (6): 273-286

[v] Oklu, R, Walker TG, Wicky, S & Hesketh R. (2010) Angiogenesis and Current Antiangiogenic Strategies for the Treatment of Cancer J Vasc Interv Radiol 21:1791–1805

[vi] Carmeliet P, Jain RK. Molecular mechanisms and clinical applications of angiogenesis. Nature. (2011) May 19;473(7347):298-307

[vii] Siemann DW, Bibby MC, Dark GG, et al. (2005) Differentiation and definition of vascular-targeted therapies. Clin Cancer Res. Jan 15;11(2 Pt 1):416-20.

[viii] Denekamp J. (1982) Endothelial cell proliferation as a novel approach to targeting tumour therapy. . Br J Cancer. 45:136–139

[ix] Hinnen P and Eskens F A L M. (2007)Vascular disrupting agents in clinical development. Br J Cancer. Apr 23, 96(8): 1159–1165.

[x] Eskander RN and Tewari KS (2014) Incorporation of anti-angiogenesis therapy in the management of advanced ovarian carcinoma – Mechanistics, review of phase III randomized clinical trials, and regulatory implications Gynecologic Oncology 132: 496-505

[xi] Burger RA (2011) Antiangiogenic agents should be integrated into the standard treatment for patients with ovarian cancer Annals off oncology 22: Suppl 8 viii65-viii68

[xii] Hall M, Gourley C, McNeish I, et al. (2013) Targeted anti-vascular therapies for ovarian cancer: current evidence British J Cancer 108: 250-258

[xiii] Gourley C, et al (2014) Proceedings of ASCO 2014: Abstract No. 5502

[xiv] U.S. Food and Drug Administration (FDA). (2005) Guidance for industry: Estimating the maximum safe starting dose in initial clinical trials for therapeutics in adult healthy volunteers. Washington, DC.

[xv] Simon R, Freidlin B, Rubinstein L, et al. (1997) Accelerated Titration Designs for Phase I Clinical Trials in Oncology. Journal of the National Cancer Institute 89(15): 1138-1147.

[xvi] Rustin GJS, Vergote I, Eisenhauer E, (2011). Definitions for Response and Progression in Ovarian Cancer Clinical Trials Incorporating RECIST 1.1 and CA 125 Agreed by the Gynecological Cancer Intergroup (GCIG). Int J Gynecol Cancer 21: 419-423

[xvii] Scher HI, Halabi S, Tannock I, et al. (2008) Design and end points of clinical trials for patients with progressive prostate cancer and castrate levels of testosterone: recommendations of the Prostate Cancer Clinical Trials Working Group. J Clin Oncol. Mar 1;26(7):1148-59.

[xviii] Oken MM, Creech RH, Tormey DC, et al. (1982) Toxicity and response criteria of the Eastern Cooperative Oncology Group. Am. J. Clin. Oncol. 5: 649-655.

[xix] Cockroft DGM. (1976) Prediction of creatinine clearance from serum creatinine. Nephron. 16:31-41.
